# Highly sensitive characterization of non-human glycan structures of monoclonal antibody drugs utilizing tandem mass spectrometry

**DOI:** 10.1038/s41598-022-19488-8

**Published:** 2022-09-06

**Authors:** Yi-Min She, Shaojun Dai, Roger Y. Tam

**Affiliations:** 1 Centre for Biologics Evaluation, Biologic and Radiopharmaceutical Drugs Directorate, Health Canada, Ottawa, Canada; 2 Development Center of Plant Germplasm Resources, College of Life Sciences, Shanghai Normal University, Shanghai, China

**Keywords:** Mass spectrometry, Glycomics, Glycoproteins

## Abstract

Glycosylation is an important attribute of monoclonal antibodies (mAbs) for assessing manufacturing quality. Analysis of non-human glycans containing terminal galactose-α1,3-galactose and *N*-glycolylneuraminic acid is essential due to the potential immunogenicity and insufficient efficacy caused by mAb expression in non-human mammalian cells. Using parallel sequencing of isobaric glycopeptides and isomeric glycans that were separated by reversed-phase and porous graphitic carbon LC, we report a highly sensitive LC MS/MS method for the comprehensive characterization of low-abundance non-human glycans and their closely related structural isomers. We demonstrate that the straightforward use of high-abundance diagnostic ions and complementary fragments under the positive ionization low-energy collision-induced dissociation is a universal approach to rapidly discriminate branch-linkage structures of biantennary glycans. Our findings reveal the structural diversity of non-human glycans and sulfation of α-galactosylated glycans, providing both an analytical method and candidate structures that could potentially be used in the crucial quality control of therapeutic mAb products.

## Introduction

Ensuring product quality of monoclonal antibody (mAb)-based therapeutics for treatments of various diseases is critical to obtain approval from regulatory agencies^1–3^. Quality control of mAb manufacturing production requires step-by-step in-process procedures through monitoring intact protein structures, product-related substances and process-induced impurities to ensure lot-to-lot consistency within acceptable variations. Glycosylation is an important post-translational modification that can affect the function and elicit potential immune responses of mAb drugs, and thus is a critical quality attribute of mAbs that needs to be carefully monitored^4^. Most therapeutic mAbs are glycosylated proteins of immunoglobulin G1 (IgG1), and to a lesser extent IgG2 and IgG4, typically consisting of a conserved glycosylation site in the fragment crystallizable (Fc) region^5^. Fc glycans play a pivotal role on the effector functions^6–12^. Additional glycosylation sites localizing at the variable region of antigen binding (Fab) can also occur, and Fab glycans have a profound impact on antibody antigenicity and neutralization^13,14^. Occurrence of heterogeneous glycans in recombinant mAbs is widely influenced by protein expression systems, cell lines, culture conditions and manufacturing processes^15,16^.

Protein expression in non-human mammalian cells is widely used for large-scale production of recombinant mAb drugs to achieve high protein yields^17^. However, these recombinant proteins expressed in Chinese hamster ovary (CHO), non-secreting mouse myeloma (NS0) and hybridoma (Sp2/0) cells have been shown to incorporate non-human glycans consisting of terminal residues of galactose-α1,3-galactose (α-Gal) and *N*-glycolylneuraminic acid (Neu5Gc)^18–21^, which are potentially immunogenic in human. Previous studies indicated that the distribution of α-Gal glycosylation of cetuximab is predominantly specific to the Fab region^22,23^, while Neu5Gc epitopes are present on both Fc and Fab domains^19,24^. By comparison, the abundances of α-Gal and Neu5Gc-containing Fc glycoforms of mAbs derived from CHO cells are too low to be recognized by anti-α-Gal IgE and anti-Neu5Gc antibodies^25^. α-Gal structures on the cetuximab Fab region have been shown to associate with causing anaphylaxis due to IgE hypersensitivity^26^, and Neu5Gc epitopes might enhance chronic inflammatory diseases via binding to endogenous anti-Neu5Gc antibodies^27^. Due to the distinguishable functions between these non-human and conventional human-type epitopes, the differentiation of glycan analogues with similar structures is of high importance. A thorough evaluation of glycan variations would therefore properly delineate and characterize non-human glycan structures and postglycosylational modifications to ensure the safety and efficiency of mAb drugs. Monoclonal antibody–based pharmaceuticals primarily comprise biantennary *N*-glycan structures, which have been characterized using liquid chromatography and tandem mass spectrometry (LC MS/MS); however, identification and quantification of closely related non-human structural isomers including terminal α1,3-Gal and Neu5Gc-linked glycans from human-type glycans represent major challenges of mAb glycomics^21,28–30^. While hydrophilic interaction liquid chromatography (HILIC) is commonly used for separating glycans from proteolytic glycopeptides and released glycans^30^, the method is restricted by low solubility of anionic and large-sized glycans in organic solvents and the poor separation of linkage-specific isomers. The traditional use of glycan chemical derivatization and extensive purification required for HILIC MS/MS analyses is time-consuming, and the additional drawbacks involved incomplete derivatization, undesired side reactions and possible degradation further limit its detection of low-abundance glycans. Increasing applications have been extended to analyses of linkage and branch-specific isomers of sialylated glycans using porous graphitic carbon (PGC) chromatography^31–34^. Progress has been achieved in the structural characterization of underivatized glycan isomers by PGC LC MS/MS^32^. We have recently demonstrated that specific diagnostic ions and complementary fragments resulting from the low-energy collision-induced dissociation (CID) at the positive ionization can be used to discriminate α2,3 and α2,6 linkage sialoglycan isomers and sulfated glycans, providing an alternative method to those by means of low-abundance cross-ring cleavages induced by high-energy or negative ionization MS/MS^30–32,35^. This method provides high sensitivity for detecting low-abundance glycans and easy data interpretation of structural isomers, as well as a simple procedure that does not require chemical derivatization^36–39^. Furthermore, our studies showed that the straightforward glycoproteomic and glycomic approaches to analyze glycopeptides and underivatized glycans using mild conditions are able to preserve the structural integrity of anionic glycans^32,40,41^, and avoid the loss of acid-labile groups under the harsh acidic or alkaline conditions. Although glycan sequencing was established for the identification of sialic acid-linkage isomers and regioisomers of sulfoglycans^32^, the previous method did not report branch-specific glycan isomers of non-human glycan epitopes that are typical glycoforms present in therapeutic mAbs, nor distinguish them from similar conventional human-type glycans.

To achieve a thorough characterization of biologically relevant glycans with non-human glycan epitopes in mAbs, herein we conducted a parallel sequencing analysis of the *N*-glycopeptides and released glycans from mAb drugs produced in CHO cells (bevacizumab, rituximab, trastuzumab, adalimumab) and murine myeloma cell lines (infliximab, cetuximab, golimumab, palivizumab). An in-house glycan library is utilized for the glycoproteomic identification and in-depth characterization of isobaric glycopeptides by reversed-phase (RP) LC MS/MS. Comparative analyses of the released glycans by endoglycosidase PNGase F were conducted by PGC LC MS/MS, and non-human glycan isomers were subsequently identified by MS/MS sequencing using high abundance diagnostic ions and complementary fragment counterparts, exoglycosidase digestion and LC elution order of glycans. Through such analyses, our results identified low abundance non-human glycans of mAbs and importantly, delineated them from conventional isobaric and isomeric human-type glycan structures. We envision the straightforward glycan sequencing methodology and candidate structures can be utilized to evaluate the presence of non-human glycans in glycoprotein-based biological products.

## Results and discussion

### Identification of nonhuman *N*-glycans and the unusual *N*-glycan sulfation involving α-Gal

To first identify non-human glycans in various mAbs, we performed RP LC MS/MS analyses of tryptic digests of eight mAbs which revealed large sets of *N-*glycopeptides (Tables S1–S5), localizing in two distinct peak regions (Fig. S1). Consistent with previous literature^42^, the short Fc glycopeptides at residues EEQYNSTYR eluted faster than the long Fab glycopeptides at residues MNSLQSNDTAIYYCAR. MS/MS measurements determined a tremendous structural diversity of high-mannose, hybrid and biantennary complex *N*-glycans in the Fc domain^43–45^. Triantennary *N*-glycans of Fab glycopeptides were exclusively detected on cetuximab (Table S4). In addition, we also observed some low-intensity glycopeptides and conducted a retrospective examination on the structural features.

The presence of high abundance diagnostic ions at *m/z* 657.28 ([Neu5Ac-Gal-GlcNAc + H]^+^), *m/z* 673.26 ([Neu5Gc-Gal-GlcNAc + H]^+^), *m/z* 528.19 ([Gal-Gal-GlcNAc + H]^+^) and the complementary fragment ions, resulting from the facile loss of glycan branching chains, provides reliable information to differentiate such *N*-glycan structures containing terminal Neu5Ac, Neu5Gc and α-Gal residues (Fig. S2). It is worth noting that the characteristic α-Gal-containing glycan ion has a much higher intensity than that of a non-α-Gal fragment ([Gal-GlcNAc-Man + H]^+^) of glycans, and is further distinguished by the presence of the minor peak at *m/z* 690.20 ([Gal-Gal-GlcNAc-Man + H]^+^) that is not possible in non-α-Gal-glycans. Using these observations, we determined that anionic glycoforms in the CHO cell-derived mAbs were mainly mono-Neu5Ac-sialylated *N*-glycans (Fig. S3, Tables S1–S2), whereas α-Gal, mono- and di-Neu5Gc-sialylated *N-*glycans were dominant in the murine myeloma cell-derived mAbs (Fig. S4, Tables S3–S5).

**Figure 1:**
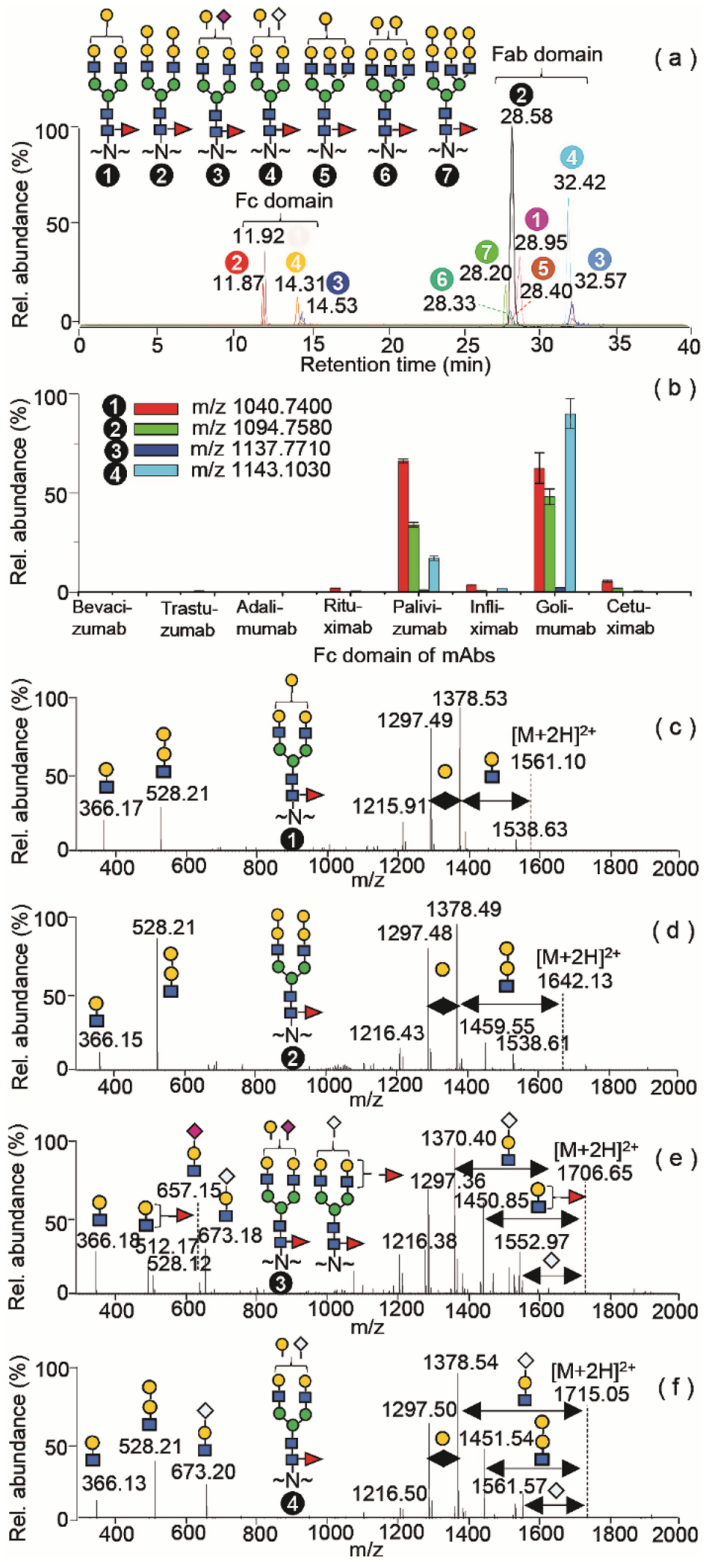
α-Gal containing *N*-glycopeptides of mAbs. (**a**) Overlaid EICs of the biantennary Fc and triantennary Fab *N*-glycopeptides of cetuximab; (**b**) Relative abundances of the Fc *N*-glycopeptides of eight mAbs examined in triplet measurements, plotted as the calculated values of peak areas from the mean and standard deviation. (**c**–**f**) MS/MS spectrum of the individual glycopeptide containing an α-Gal composition illustrated by compound numbers within the black circles, respectively, and the precursor ions are shown in the figure (**b**). The compound 3 is a representative structure derived from a mixture of isobaric glycans containing Neu5Ac and Neu5Gc.

The α-Gal-containing *N*-glycans were all fucosylated in the mAb glycopeptides (Tables S3–S4), and most of them have been known to exist as glycans with α1,6 core-fucosylation^21,28,46^. Fig. 1a,b show several types of glycopeptides containing α-Gal *N*-glycans (Table S6), in which the relatively high abundance glycoforms contain single and double α-Gal residues on the biantennary *N*-glycan structures of Fc domains and the Fab region of cetuximab. The aforementioned diagnostic α-Gal-containing glycan ions accompanied by the high-intensity complimentary fragments in the MS/MS spectra were utilized to readily identify biantennary non-human glycans containing branched terminal α-Gal and Neu5Gc (Fig. 1c–f). Analysis of the MS/MS spectrum of the triply charged ion at *m/z* 1138.10 (Fig. 1e) showed a more complex fragmentation pattern; the simultaneous presence of two pairs of the fragments at *m/z* 528.12 ([α-Gal-Gal-GlcNAc + H]^+^) and *m/z* 657.15 ([Neu5Ac-Gal-GlcNAc + H]^+^), *m/z* 512.17 ([Fuc-Gal-GlcNAc) + H]^+^) and *m/z* 673.18 ([Neu5Gc-Gal-GlcNAc + H]^+^) indicates a glycan mixture.

**Figure 2:**
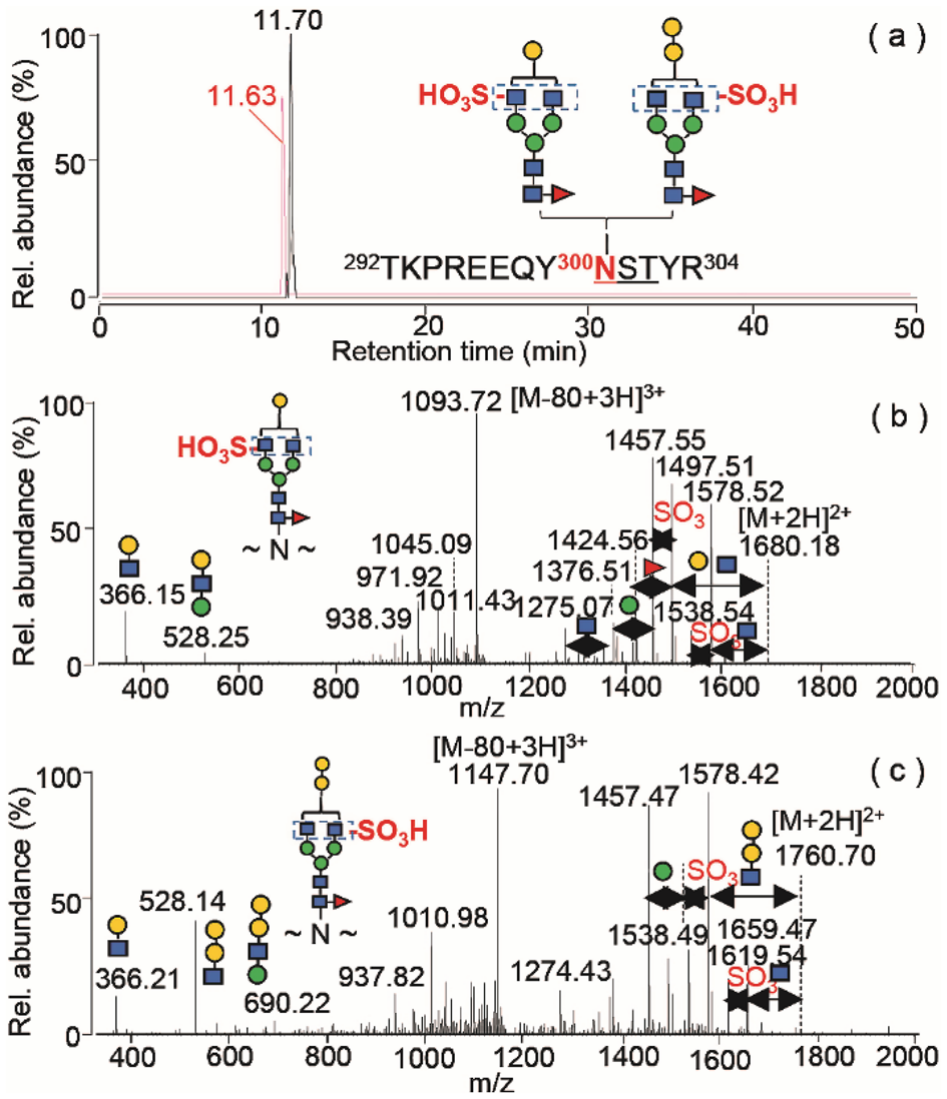
Sulfated N-glycopeptides of tryptic palivizumab. (**a**) EICs of the triply charged ions at *m/z* 1120.1225 (black line) and *m/z* 1174.1401 (red line); (**b**) MS/MS spectrum of the G1F containing N-glycopeptide of *m/z* 1120.1225; (**c**) MS/MS spectrum of the [G1F + α-Gal] containing glycopeptide of *m/z* 1174.1401.

Notably, our database search identified the sulfation of α1,3-Gal-containing Fc *N*-glycans in palivizumab, golimumab, infliximab and cetuximab. Figure 2 shows the MS/MS spectra of sulfated palivizumab *N*-glycopeptides at residues TRPREEQYNSTYR at the triply charged ions of *m/z* 1120.1225 and *m/z* 1174.1401. Two glycopeptides containing either a conventional single Gal-β1,4-GlcNAc or a non-human Gal-α1,3-Gal-β1,4-GlcNAc extension at the termini of *N*-glycans were co-eluted as an integrated peak at ~ 11.70 min (Fig. 2a). Both glycopeptides displayed the base peak fragments of the triply charged ion [M-80 Da + 3H]^3+^, and the doubly charged fragment ions followed by the consecutive losses of GlcNAc and neutral molecules of 80 Da (Fig. 2b,c). Accurate mass measurements of the glycopeptides indicates the 80 Da molecule is a sulfate (SO_3_^2−^, 79.9658 u) rather than phosphate group (HPO_3_^−^, 79.9663 u), resulting in mass errors of 0 ppm and −3 ppm, respectively. We propose that the digalactosylated structure (Fig. 2c) contains an α-Gal-Gal-GlcNAc-Man branch, as indicated by the presence of the two diagnostic ions at *m/z* 528.14 ([α-Gal-Gal-GlcNAc + H]^+^) and *m/z* 690.22 ([α-Gal-Gal-GlcNAc-Man + H]^+^), and the intensity of the former fragment is higher than that of the *m/z* 366.21 ([Hex-GlcNAc + H]^+^) peak. In contrast, the monogalactosylated species (Fig. 2b) shows a relatively higher fragment at *m/z* 366.15 compared to the ion at *m/z* 528.25 (with no additional fragment at *m/z* 690.22), which can be attributed to the terminal branch fragmentations of Gal-GlcNAc and Gal-GlcNAc-Man, respectively. The possible human-type digalactosylated glycan G2F, possessing the identical mass to that of the digalactosylated glycan containing a terminal Gal-α1,3-Gal residue in Fig. 2c, is excluded, as the predicted fragmentation would lead to a high-intensity branch-specific peak at *m/z* 366.15 ([Gal-GlcNAc + H]^+^) as in Fig. 2b, which is not observed in Fig. 2c. We thus conclude that sulfation occurs on an *N*-glycan comprising the non-human α-Gal epitope.

Overall, our analyses at the glycoproteomic level revealed the co-existence of non-human glycans in the Fc and Fab domains of mAb products, with varying abundance. Interestingly, we observed negatively charged sulfation at α-Gal containing *N*-glycans of the recombinant mAbs expressed from the murine myeloma cells. While sulfation commonly occurs in hybrid and complex-type *N*-glycans of therapeutic proteins at the C-3 of Gal, C-6 of GlcNAc and C-4 of *N*-acetylgalactosamine (GalNAc) residues of terminal Gal-GlcNAc and GalNAc-GlcNAc sequons^40,41,47^, the finding of sulfated α-Gal-glycans in mAb drugs suggests a previously unrecognized quality attribute that may require monitoring, and further understanding of its biological properties.

### Differentiation of isobaric Neu5Gc and α-Gal-containing glycopeptides

**Figure 3:**
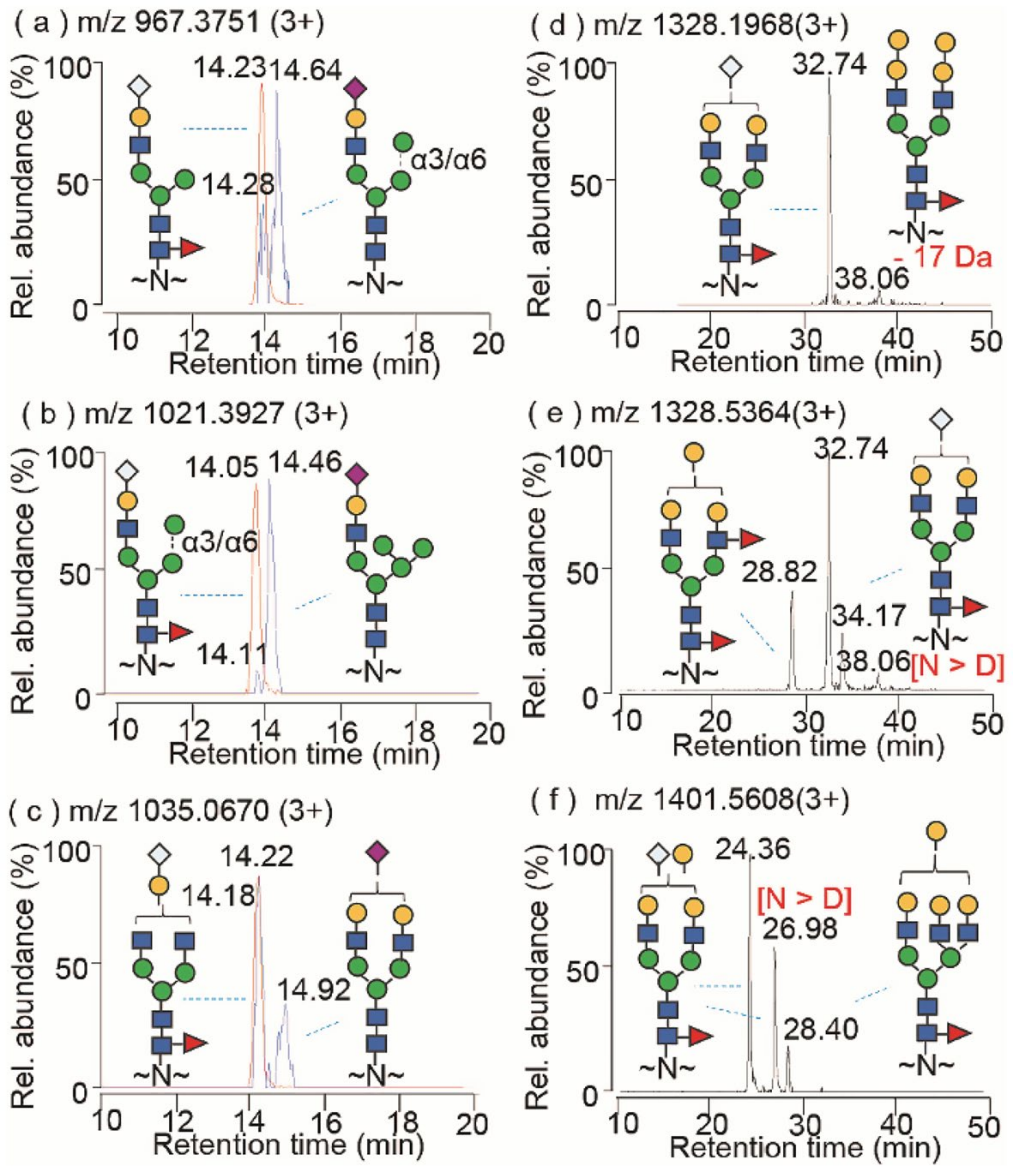
EICs of isobaric *N*-glycopeptides varying in glycan structures and peptide modifications. (**a**–**c**) Fc sialoglycopeptides of trastuzumab derived from CHO cells (blue lines) and infliximab derived from murine myeloma cells (red lines); (**d**–**f**) Cetuximab α-Gal containing Fab *N*-glycopeptides. Modifications include the identified deamidation of Asn to Asp (N > D) and the neutral loss of a 17 Da molecule of the peptides.

A thorough examination of LC MS/MS data showed that RP LC is capable of resolving isobaric structures of Fc and Fab glycopeptides varying in glycan compositions, but not glycosidic linkage (i.e. positional and branching) isomers. Human vs non-human isobaric sialoglycopeptides in the Fc region with the identical masses of disaccharide residues, i.e. Neu5Ac-Gal (C_17_H_27_O_13_N; 453.1483 u) vs Neu5Gc + Fuc (C_17_H_27_O_13_N; 453.1483 u)^48^, were easily discriminated by the characteristic ions at *m/z* 657.26 ([Neu5Ac-Gal-GlcNAc + H]^+^) versus *m/z* 673.29 ([Neu5Gc-Gal-GlcNAc + H]^+^) in MS/MS spectra (Fig. S5). The RP LC chromatograms of glycopeptides often exhibited distinct peaks with close retention time (RT) between the isobaric glycoforms, in which the compositions containing Neu5Gc + Fuc eluted slightly earlier than the Neu5Ac-Gal structures (Fig. 3a–c, Fig. S5).

Similar analyses conducted on the isobaric biantennary and triantennary *N*-glycopeptides from the Fab region of cetuximab delineated terminal α-Gal from Neu5Gc residues (Fig. 3d–f, Figs. S6–S7). Based on the characteristic glycan ions and complementary fragments, the distinct glycan structures of separated glycopeptides by RP LC MS/MS remain discernable. A representative EIC of cetuximab in Fig. 3e shows four chromatographic peaks at 28.82 min, 32.74 min, 34.17 min and 38.06 min, corresponding to the glycopeptides of triply charged ions at *m/z* 1328.5364, *m/z* 1328.1968, *m/z* 1328.5249 and *m/z* 1328.1960, respectively. The presence of abundant diagnostic ions at *m/z* 512.23 ([Gal-GlcNAc-Fuc + H]^+^) and *m/z* 528.23 ([Gal-Gal-GlcNAc + H]^+^) in the 28.82 min peaks, and *m/z* 673.26 ([Neu5Gc-Gal-GlcNAc + H]^+^) in the 32.74 min peak (along with the complementary Y fragments at *m/z* 1737.72 ([MH-511]^+^), *m/z* 1729.71 ([MH-527]^+^) and *m/z* 1656.22 ([MH-672]^+^) respectively) with the neutral losses from the glycopeptide precursor ions (Fig. S6d–e), identified these two chromatographic peaks as different glycopeptides which share the Fab peptide sequence but vary with α-Gal + Fuc or Neu5Gc at the glycan branch side chains^32^. In contrast, the MS/MS spectrum of lower intensity glycopeptide of *m/z* 1328.5249 at 34.17 min resulted in a similar fragmentation pattern to that of the former glycopeptide of *m/z* 1328.1968 at 32.74 min, which defines the identical Neu5Gc-sialyl *N*-glycan structure and 1 Da mass difference at the Fab peptide region with the alteration of asparagine to aspartic acid in the native or deamidated form (+ 0.9840 Da) (Table S4). Intriguingly, the lowest intensity glycopeptide of *m/z* 1328.1960 at 38.06 min has a high abundance characteristic α-Gal-containing fragment ion at *m/z* 528.17 ([α-Gal-Gal-GlcNAc + H]^+^) and the complementary fragment at *m/z* 1728.87 ([MH-527]^+^) (Fig. S6g), but no Neu5Gc-branched diagnostic ion at *m/z* 673.26, suggesting the biantennary *N*-glycan structure comprising solely two α-Gal branched chains.

Unexpectedly, the Fab glycopeptide of cetuximab at the triply charged ion of *m/z* 1401.5608 exhibited three isobaric glycoforms (Fig. 3f and Fig. S7a). MS/MS analyses of the glycopeptides revealed different glycan compositions (Fig. S7b,c), and only the low intensity glycopeptide at *m/z* 1401.5633 at 28.40 min was matched to the predicted triantennary glycan structure (Gal_4_-GlcNAc_5_-Man_3_-Fuc) (Table S4). The fragmentation of the abundant glycopeptides at *m/z* 1401.2227 and *m/z* 1401.5500 at 24.36 min and 26.98 min displayed similar MS/MS fragmentation patterns, with sequential losses of monosaccharides, corresponding to the glycan composition containing Hex_6_-HexNAc_4_-Neu5Gc-Fuc. The structure was assigned to a biantennary glycan with two functional groups of terminal Neu5Gc and α-Gal on G2F based on the diagnostic ions at *m/z* 528.18 ([α-Gal-Gal-GlcNAc + H]^+^) and *m/z* 673.23 ([Neu5Gc-Gal-GlcNAc + H]^+^) and the complementary Y fragments (Fig. S7b). The remaining mass differences of 57.0276 Da and 58.0095 Da between the measured values of 4201.6525 Da and 4202.6344 Da of the two glycopeptides, and the predicted cysteine-carbamidomethylated glycopeptide (4144.6249 Da) suggest additional S-carbamidomethylation of methionine (+ 57.0215 Da) in the peptide sequence (MNSLQSNDTAIYYCAR) from iodoacetamide treatments, and the deamidation of asparagine residue (+ 0.9840 Da) of the latter peptide (Table S4). Both glycopeptides yielded the high abundance fragments following the losses of 105 Da from the glycopeptide precursor ion, corresponding to 2-(methylthio)acetamide (C_3_H_7_NOS, 105.0248 Da)^40^. Therefore, glycopeptide sequencing using high abundance diagnostic ions and complementary fragments simplified data interpretation to discriminate the subtle structural difference from either glycan compositions or peptide modifications of glycopeptide analogues.

### Structural diversity of α-Gal containing glycan isomers

To better identify the structure of glycan isomers, mAb glycoproteins were digested by PNGase F and the released glycans were analyzed by PGC LC MS/MS. As demonstrated by our previous studies^32^, the structural isomers of native glycans can be well-resolved using positive ionization low-energy CID. Glycomic screening of eight mAbs revealed the most abundant glycans of G0F, G1F and G2F (Fig. S8 and Tables S7–S8). Pairs of agalactosylated, monogalactosylated and digalactosylated glycans are reasonably recognized as structural anomers in which the α-anomer has higher abundance and longer RT than the β-anomer^32^. The chromatographic profile shows that the relative intensities of the neutral glycans are comparable to those glycopeptides obtained by RP LC MS/MS (Fig. S1).

Subsequent structure assignment of low abundance glycans was accomplished based on the elution order of glycan isomers, abundant MS/MS fragments and exoglycosidase sequencing^32^. Indeed, LC retention time of biantennary glycans represents the most critical element for structural elucidation of glycan isomers due to its direct relevance to the isomeric separation by PGC^28^. Extensive studies have shown that the complex glycan with an elongated branch chain at the α6 antenna of the *N*-acetyl chitobiose (GlcNAc_2_)-containing-trimannosyl core elutes faster than the isomeric glycan at the α3 antenna on PGC^28,35,49–51^.

To gain further insight into delineating differences in the structure-based MS/MS fragmentation of biantennary glycans, we examined the PGC LC MS/MS fragmentation patterns of G1 and G1F glycans from glycan standards and known structural isomers of fetuin and mAbs (Figs. S9–S10). Branch-specific fragmentation of the GlcNAc-β1,2-Man glycosidic linkage of the α3 antenna consistently yielded higher intensity B and Y fragments than those of the corresponding linkage on the α6 antenna. Structural modeling of the glycans using Glycam shows potential stabilizing intramolecular hydrogen bonds between the C3 hydroxyl of the α6 mannose residue of the trimannosyl core with the cyclic oxygen atom of the adjacent non-reducing GlcNAc residue, with the α6 antenna having a slightly shorter distance compared to the α3 antenna (2.40 vs 2.51 Å); an additional potential hydrogen bond is observed between the C2 NHAc group of the core GlcNAc residue with the non-reducing GlcNAc residue of the α6 antenna, an interaction that is absent in the α3 antenna (Fig. S11a,b). Such structural features are also visible between the β1,2-GlcNAc linkage branch of α6 antenna and the trimannosyl core in triantennary and tetraantennary glycans (Fig. S11c,d). These data are consistent with the fragmentation patterns of the other glycans reported previously^28^.

Using this method, along with retrospective analyses on glycan isomers, we confirmed that (1) biantennary complex glycan isomers containing a reducing end *N*-acetyl chitobiose core were separated on an elution order of the glycan with an elongated branch chain on the α6 antenna followed by that on the α3 antenna by PGC; (2) the presence of high abundance diagnostic ions, accompanied by the complementary MS/MS fragments with the neutral loss from precursor ions, is often indicative of the branching side chain of a glycan; (3) MS/MS fragmentation of a biantennary complex glycan yields a higher intensity fragment with the neutral loss of branch at the α3 antenna than that at the α6 antenna; (4) the loss of water from complementary Y fragments to form [Y-18]^+^ ions result from either the reducing end of the glycan β-anomer, or the branch-specific single fragment at the intact α6 antenna of glycan α-anomers, corroborating our recent report^32^. We thus establish the acceptable criteria for sequencing biantennary glycan isomers which constitute the base for subsequent characterization of isomeric structures of non-human glycans in mAb drugs (Table S9).

**Figure 4:**
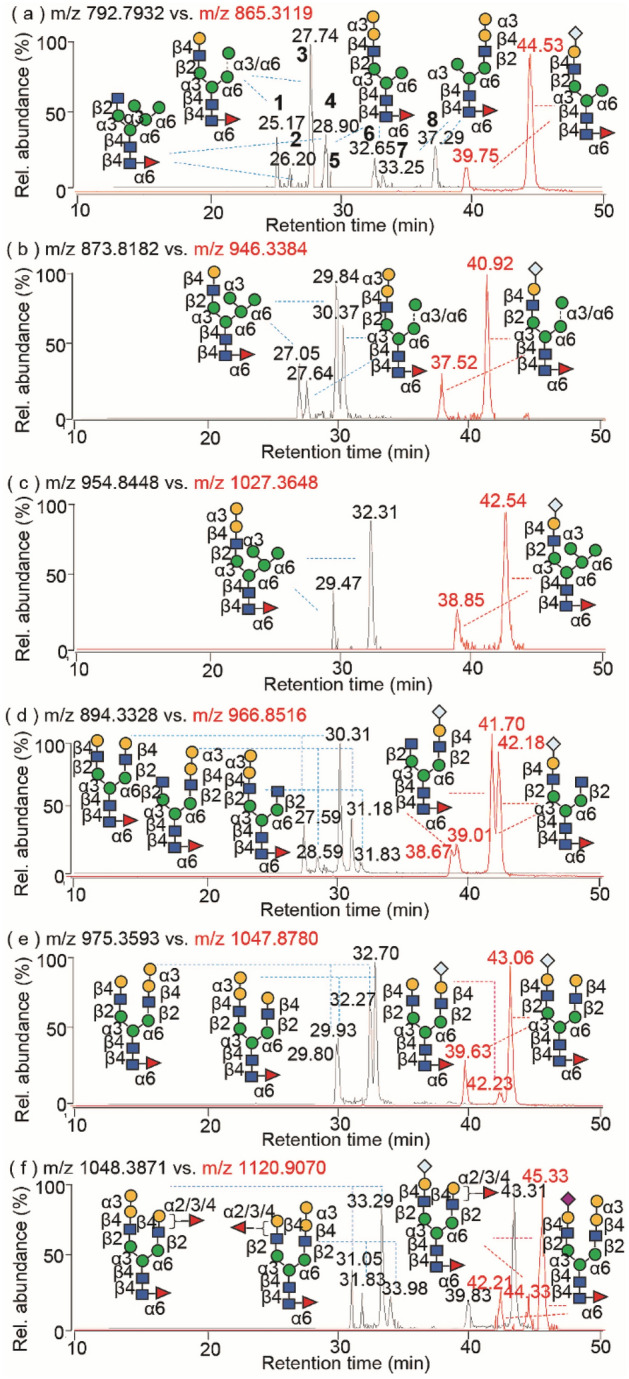
Structural comparison of the non-human glycan isomers between terminal residues of α-Gal and Neu5Gc. (**a**–**f**) EICs of the typical glycans containing structurally consistent α-Gal (black lines) and Neu5Gc (red lines) at the region-specific locations released from mAbs.

With the ability to better separate released glycans using PGC compared to glycopeptides using RP LC, and importantly without the need for further chemical derivatization, we delineated several isomeric glycoforms, including low abundance non-human glycans from human-compatible glycans (Fig. 4). A parallel comparison of the EICs of proteolytic glycopeptides and the released glycans from hybrid and pseudohybrid glycans containing potential α3-Gal linkages at *m/z* 792.7932 revealed up to eight isomers in the murine myeloma cell-derived mAbs (Fig. 4a and Fig. S12). In light of similar MS/MS fragmentation patterns, the pairs of glycans **1** and **3**, **2** and **4**, **5** and **6**, **7** and **8** were identified as β- and α-anomers, respectively, of each pair of glycans (Fig. S13). Only glycans **1** and **3** showed high abundance of the diagnostic ion at *m/z* 366.1 ([Gal-GlcNAc + H]^+^) and its high-intensity complementary Y fragment at *m/z* 1219.33 ([MH-365]^+^), confirming a Gal-GlcNAc-branched chain at the cored α1,3 antenna of a Man_4_-based hybrid glycan. Exoglycosidase sequencing with α1-2,3 mannosidase revealed compounds **1** and **3** have an unbranched mannose residue localizing at the extended α1,3 arm from the core α1,6 antenna (Fig. S14). In contrast, the MS/MS spectra of glycan isomers of **2** and **4** display the high-intensity Y fragments at *m/z* 1381.42 with the neutral loss of GlcNAc from the precursor ion at *m/z* 1584.58, suggesting a GlcNAc branch at the cored α1,3 antenna of Man_5_-based hybrid glycans. It is also interesting to note that the relatively high abundance isomers of **5** and **6**, **7** and **8** were extensively detected in murine myeloma cell-derived mAbs (Fig. S12f–j). The occurrence of the most abundance diagnostic ion at *m/z* 528.18 ([Gal-Gal-GlcNAc + H]^+^) and its high-intensity complementary fragment at *m/z* 1057.37 ([MH-527]^+^) indicates a terminal α-Gal residue in the glycan, which was verified by exoglycosidase sequencing by α1-3,6 galactosidase (Fig. S14). Treatments with α1-2,3 mannosidase revealed that the isomers **7** and **8** can be cleaved at the unbranched monomannose residue, and the structure is assigned to a Gal-α1,3-Gal-GlcNAc linkage to the α1,6 antenna (Figs. S12, S14). Correspondingly, isomers **5** and **6** were reasonably assigned to the anomeric structures of the glycan containing an α-Gal branch at the α1,3 antenna. Similar structures of Man_4-5_-based α-Gal-containing glycans in Fig. 4b,c were identified by MS/MS fragmentations (Fig. S15). Therefore, analyses of glycan fragmentations unambiguously identified the low-abundance non-human glycans **5–8** containing the α-Gal epitope from the relatively high-abundance conventional human-type glycans **1–4**.

Next, extended analyses of positional glycan isomers containing α-Gal residues in bi- and tri-antennary glycans were performed (Fig. 4d,e, Figs. S16–S18). Three pairs of biantennary glycan isomers including human-compatible G2F, and non-human α-Gal-containing G1(3)F and G1(6)F were identified (Fig. S16). Consistent with other complex G1 and G1F glycan isomers as mentioned above, α-Gal residues on the α6-antenna eluted earlier relative to its presence on the α3-antenna, and was further confirmed by the higher intensity of the diagnostic ion following cleavage of the β1,2 glycosidic linkage between the GlcNAc and mannose residue on the α3-antenna (Fig. S16). Using similar analyses, we further discovered several glycan isomers containing mono-, di- and tri-α-Gal linkages of biantennary G2F and triantennary G3F in the mAbs (Figs. S17–S18).

### Structural consistency between mono-Neu5Gc-sialylated and mono-α-galactosylated glycans at region-specific locations

Apart from α-Gal, terminal Neu5Gc is also produced in non-human cells. Further analyses of the released mAb glycans show the mono-Neu5Gc-sialylated glycans are the predominantly anionic glycoforms, although di-Neu5Gc linkages were also present in the biantennary glycans of mAbs produced in murine myeloma cells, consistent with previous reports^24^. The separation of afucosylated and fucosylated sialoglycans by PGC revealed several Neu5Gc-linkage branching isomers in the released underivatized glycan structures (Figs. S19, S20). MS/MS analyses identified several glycans with the typical Man_3-5_-based hybrid structures, which contain the Neu5Gc-sialylated epitope (Fig. 4a–c, Fig. S19a–c, e–g). As expected, the branch-specific isomers of Neu5Gc-sialylated biantennary glycans were reasonably present on the truncated branching complex glycan structures of G1S1, G1FS1 and G2FS1 (Fig. S9d, h, i), which can be differentiated by the diagnostic ions at *m/z* 673.23 ([Neu5Gc-Gal-GlcNAc + H]^+^) and *m/z* 366.14 ([Gal-GlcNAc + H]^+^) and the complementary fragment Y/[Y-18] ions from the neutral losses of branching side chains (Fig. S20c–h). The relative abundances of diagnostic ions are dependent on branching locations in which the cleavage of glycosidic linkage at the α3 antenna is more easily cleaved than the α6 antenna. The Neu5Gc-branched complex glycan at the α3 antenna elutes after the α6 antenna isomer, providing supporting evidence to the aforementioned acceptable criteria of MS/MS glycan sequencing. Interestingly, a side-by-side comparison of the observed glycan structures between mono-Neu5Gc-sialylated and mono-α-galactosylated glycans expressed in mAbs produced in murine myeloma cells showed a high structural consistency at the branch region-specific locations in murine myeloma cell-derived mAbs, as observed in the EICs of the released glycans (Fig. 4). Although the relative abundance of each individual non-human glycan is low, our ability to distinguish their antenna-specific location enables grouping of non-human epitopes based on their location and N-glycan type to determine their overall relative distribution (Table S10, Fig. S21). The specific location of carbohydrate residues in N-glycans can be important in receptor binding affinity and bioactivity^52–54^, and whether this remains true for non-human glycan epitopes requires further investigation. Hierarchical clustering analysis further shows the relative similarities in the presence of Neu5Gc *vs* α-Gal epitopes in different types of glycans (i.e. complex vs. (pseudo)hybrid vs. antenna fucosylated glycans) (Fig. S21).

### Heterogeneous termini of α-galactosylated and sialylated glycans

**Figure 5:**
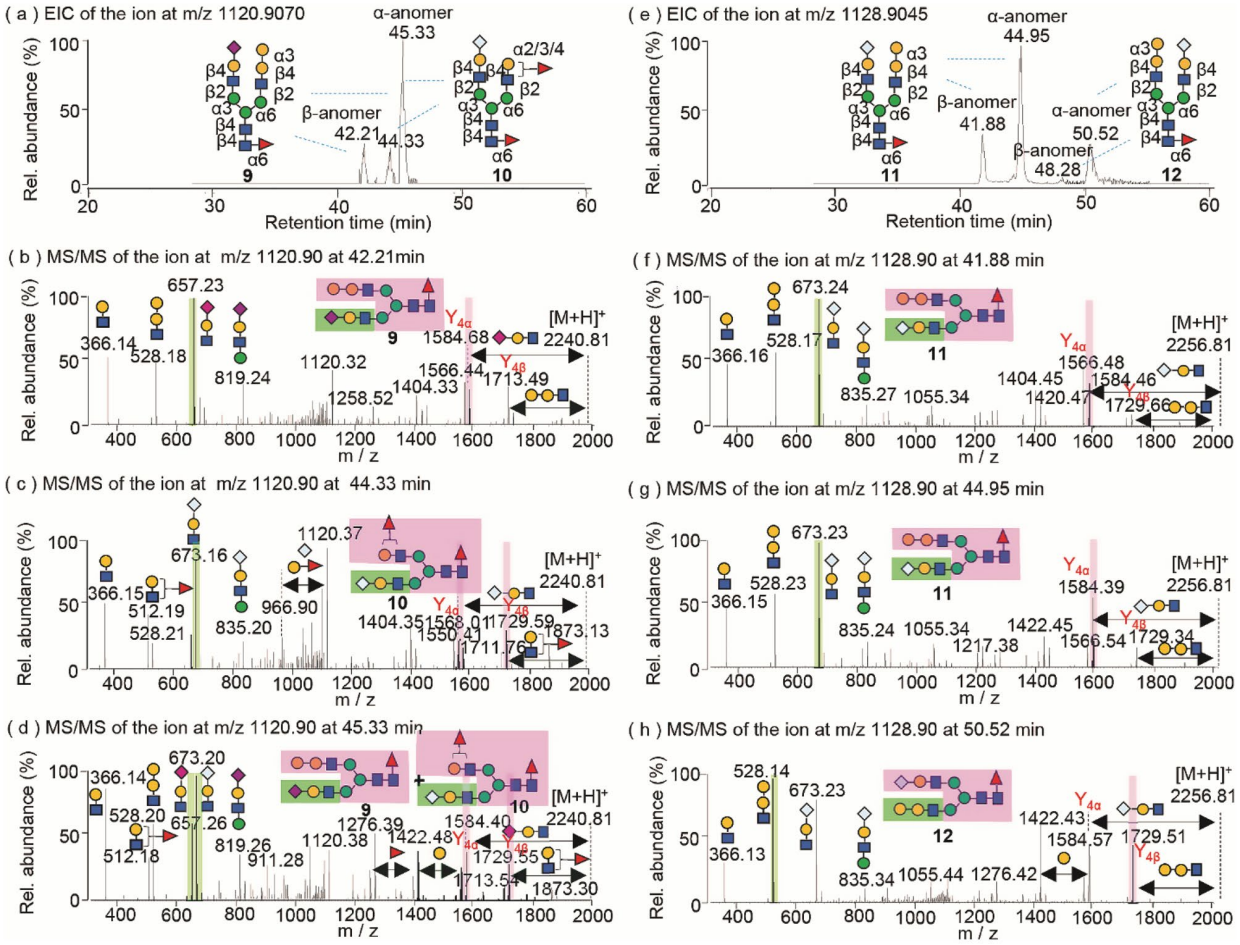
Cetuximab sialoglycan isomers containing di-terminal α-Gal and Neu5Gc. (**a**, **e**) EICs of the doubly charged ions of *m/z* 1120.9070 and *m/z* 1128.9045; (**b**–**d**, **f**–**h**) MS/MS spectra of the glycan ions of *m/z* 1120.90 and *m/z* 1128.90, respectively.

In addition to singly Neu5Gc-branched sialoglycans, the biantennary sialoglycans bearing heterogeneous termini of Neu5Ac, Neu5Gc and α-Gal at the non-reducing ends were also remarkably detected (Fig. 5). The EIC of the sialoglycan at *m/z* 1120.906 shows three well-separated peaks at 42.21 min, 44.33 min and 45.33 min (Fig. 5a), in which subtle differences of the glycan structures were identified by MS/MS (Fig. 5b–d). Pairs of the diagnostic ions containing terminal α-Gal and sialic acids were observed as the most common feature of the branch-specific glycan isomers. Similarly, the information obtained from these diagnostic ions and the high abundance complementary fragments can be used for the structural interpretation of branching linkage isomers^32^.

Based on the higher relative abundance of the characteristic ions at *m/z* 657.23 ([Neu5Ac-Gal-GlcNAc + H]^+^) vs *m/z* 528.18 ([Gal-Gal-GlcNAc + H]^+^, and the complementary Y/[Y-18] fragments at *m/z* 1584.68 ([MH-656]^+^) and *m/z* 1566.44 ([MH-656 −18]^+^) vs *m/z* 1713.49 ([MH-527]^+^) (Fig. 5b), respectively, we assigned the glycan isomer **9** at 42.21 min to contain a terminal Neu5Ac branch at the α3-antenna and α-Gal branch at the α6-antenna of G2F. The presence of the abundant Y_4α_/[Y_4α_-18] fragment pair, derived from the consequential losses of Neu5Ac-Gal-GlcNAc and water from the precursor ion, indicates the glycan structure is a β-anomer configuration. In the MS/MS spectrum of isomer **10** at 44.33 min (Fig. 5c), it is worth noting that the fragment ion at *m/z* 528.21 ([Gal-GlcNAc-Man + H]^+^) appeared at the low intensity without its complementary Y fragment ion at *m/z* 1713.60 ([MH-527]^+^), which excludes the possibility of a terminal α-Gal structure. Instead, the occurrence of the abundant fragment ion at *m/z* 512.19 ([Fuc-Gal-GlcNAc + H]^+^), together with its complementary ion at *m/z* 1729.59 ([MH-511]^+^) is unique, and implies the antenna-specific fucosylation at the branching Gal-GlcNAc of isomer **10**. Similarly to isomer **9**, the higher relative abundance of the characteristic ion at *m/z* 673.16 ([Neu5Gc-Gal-GlcNAc + H]^+^) and the complementary fragment ions of Y_4α_/[Y_4α_-18] at m/z 1568.01 ([MH-672]^+^) and *m/z* 1550.41 ([Y_4α_-H_2_O]^+^) defined the branched Neu5Gc-Gal-GlcNAc at the α3-antenna and subsequently the antenna-fucosylated Gal-GlcNAc at the α6-antenna of isomeric glycan **10**. In comparison, the chromatographic peak of the glycans at 45.33 min appears at a relatively high intensity when compared to isomers **9** and **10** (Fig. 5a). Interestingly, four abundant diagnostic fragment ions at *m/z* 512.18 ([Fuc-Gal-GlcNAc + H]^+^), *m/z* 528.20 ([Gal-Gal-GlcNAc + H]^+^), *m/z* 657.23 ([Neu5Ac-Gal-GlcNAc + H]^+^) and *m/z* 673.20 ([Neu5Gc-Gal-GlcNAc + H]^+^) were simultaneously detected together with their complementary fragments in the MS/MS spectrum (Fig. 5d), indicating the heterogeneous termini of antenna fucose, α-Gal, Neu5Ac and Neu5Gc residues in the glycan(s). Parallel comparisons of MS/MS spectra among Fig. 5b–d suggest that the mixture of α-anomeric glycan isomer **9** and an antenna-fucosyl regioisomer of glycan **10** co-contribute to the fragmentation pattern of glycans at 45.33 min.

Structural analyses were continued on the glycan isomers consisting of bi-terminal Neu5Gc and α-Gal. PGC LC MS/MS analyses revealed three major peaks in the EIC of the ion at *m/z* 1128.9045 (Fig. 5e), and similar MS/MS fragmentation patterns of the glycan isomers involving two abundant diagnostic fragments at *m/z* 528.17 ([Gal-Gal-GlcNAc + H]^+^) and *m/z* 673.24 ([Neu5Gc-Gal-GlcNAc + H]^+^) (Fig. 5f–h) with varying relative intensities. Using the established glycan sequencing rules, we can easily identify two distinct structures of glycan isomers **11** and **12**. The MS/MS spectra of glycans at 41.88 min and 44.95 min displayed higher diagnostic ion at *m/z* 673.24 ([Neu5Gc-Gal-GlcNAc + H]^+^) and the complementary fragment at *m/z* 1584.46 ([MH-672]^+^) than those of the other branch cleavage at *m/z* 528.17 ([Gal-Gal-GlcNAc + H]^+^) and *m/z* 1729.66 ([MH-527]^+^) (Fig. 5f,g), indicating the β- and α-anomers of glycan isomer **11** that comprise a Neu5Gc branch at the 3-antenna and the α-Gal branch at the 6-antenna. On the contrary, the fragmentation of glycan at 50.52 min yielded slightly lower intensities of the fragments of *m/z* 673.23 ([Neu5Gc-Gal-GlcNAc + H]^+^) and *m/z* 1584.57 ([MH-672]^+^) than those fragments of m/z 528.14 ([Gal-Gal-GlcNAc + H]^+^) and *m/z* 1729.51 ([MH-527]^+^) (Fig. 5h), suggesting an opposite location of the α-Gal and Neu5Gc-substitutions of isomer **12**. Glycopeptide sequencing of mAbs showed that these glycans containing di-termini of Neu5Ac, Neu5Gc, α-Gal and antenna fucose are localized in both Fc and Fab regions of the murine cell-derived mAbs (Table S4).

## Conclusions

In summary, through the comprehensive characterization of a wide variety of glycan isomers via parallel measurements of glycopeptides and released underivatized glycans using RP and PGC LC MS/MS, we demonstrated the suitability of a highly sensitive glycan sequencing method for a rapid differentiation of low abundance non-human glycan isomers from human-compatible glycan counterparts. These methods are capable of accurately determining the site-specific location and structures of mAb glycan isomers, while the highly sensitive sequencing method of glycans is readily available to achieve the throughput, accuracy and reproducibility in analyzing large glycomic datasets. A quantitative comparison of structurally consistent glycan components is feasible to evaluate the distribution of non-human glycan isomers by implementing the relative peak areas of EICs.

In this work, we have also identified sulfation of α-Gal-containing glycans, confirmed the structural consistency of α-galactosylated and Neu5Gc-sialylated biantennary glycans, and a wide distribution of homogenous and heterogeneous non-human epitopes containing mono- and di-termini of Neu5Gc, α-Gal and possible antenna α2/3/4-fucose of mAbs in murine myeloma cells. The structural and functional characterization of glycan attributes are particularly important for regulating the quality of mAb biosimiliars. Our findings increase understanding of the structural diversity and the cellular distributions of non-human glycans of recombinant mAbs. The results provide glycan targets and candidate structures for high-throughput analysis of non-human glycomics, and thus could assist in the quality control of future biotherapeutics production.

## Methods

### Materials

Acetonitrile (ACN), ammonium bicarbonate (NH_4_HCO_3_), dithiothreitol (DTT), iodoacetamide, formic acid (FA) and bovine fetuin (Catalog no. F2379) were obtained from Sigma-Aldrich. PNGase F, α1-2,3 mannosidase, α1-3,6 galactosidase and α2-3,6,8 neuraminidase were purchased from New England Biolabs. LacZ β-galactosidase was purchase from Roche Diagnostics. mAbs were obtained from pharmaceutical companies.

Micro PGC column materials were obtained from Mandel Scientific Corporation, and capillary Hypercarb PGC materials (particle size 3 µm, pore size 250 Å) were obtained from Thermo Fisher Scientific. Fused silica capillary tubing was purchased from Polymicro Technologies Inc. Capillary PGC columns were packed in-house as reported previously^32^.

### Glycoproteomic analyses of mAb glycopeptides using RP C18 column

100 µg of mAb were reduced with 50 mM DTT at 60 °C and alkylated with 100 mM iodoacetamide for 1 h, followed by dialysis against 10 mM NH_4_HCO_3_ and speedvac dry using a refrigerated CentriVap concentrator (Labconco). The proteins were digested at 37 °C overnight using trypsin (Promega) (enzyme-to-protein, w/w, 1/100) in 25 mM NH_4_HCO_3_. Resulting peptides were dried, dissolved in 0.2% FA and analyzed by RP LC MS/MS on an Orbitrap Fusion Lumos coupled with an Easy-nLC 1200 system (Thermo Fisher Scientific)^41^.

### Glycomic analyses of released mAb glycans using PGC LC MS/MS

Alkylated proteins were loaded into tube gels followed by in-gel digestion using 1 µL of PNGase F in 25 mM NH_4_HCO_3_ at 37 °C overnight. The released glycans were extracted using ACN/water (v/v, 1:1) and ACN. A portion of glycans was further cleaved with α1-2,3 mannosidase or α1-3,6 galactosidase in 50 mM sodium acetate containing 5 mM calcium chloride (pH 5.5) at 37 °C overnight. The products were purified using an in-house made PGC microcolumn.

The dried glycans were dissolved in 0.5% FA, and analyzed by nanospray PGC LC MS/MS on an Orbitrap Fusion mass spectrometer coupled with a nanoAcquity ultra performance LC or Acquity UPLC M-class system (Waters)^32^. The glycans were trapped onto a PGC column (150 µm × 2 cm) and separated with an analytical PGC column (100 µm × 20 cm) at 600 nL min^−1^ using a 60-min gradient of 10–25% of ACN in 0.1% FA. The Orbitrap MS was acquired at a resolution at 120,000 followed by the ion-trap fragmentation of multiply charged ions using CID at the normalized collision energy of 25%.

### Construction of a mAb *N*-glycan library for glycopeptide identification

The in-house mAb *N*-glycan library was constructed based on the common structures of *N*-glycans derived from the glycan biosynthesis pathway (Fig. S22). Biantennary and triantennary *N*-glycans were selected from literatures and LC MS/MS analyses of mAb drugs. Bisected *N*-acetylglucosamine (GlcNAc) glycans were incorporated for mAbs derived from human cells, although non-human mammalian cells are known to lack the gene encoding GNT-III for production of GlcNAc-bisecting *N*-glycans. Possible modifications were considered including phosphorylation of high-mannose *N*-glycans, sulfation of complex *N*-glycans and *O*-acetylation of sialic acids^32^. Protein sequences were collected from the Drugbank (https://www.drugbank.ca). Tryptic glycopeptides were identified by database search using Byonic software (Protein Metrics Inc.) and verified by manual inspection^41^. MS/MS sequencing by diagnostic ions and complementary fragments was also utilized to elucidate unknown glycopeptides for the structural identification and in-depth characterization of isobaric glycopeptides separated by RP LC MS/MS.

### Elucidation of structural glycan isomers

Isomeric glycans were assigned according to the knowledge of the glycan biosynthetic pathway, diagnostic ions, accurate masses and MS/MS fragment patterns of glycans^32,55^. Glycan structures were further validated by LC elution order of glycan isomers and exoglycosidase sequencing. To simplify data interpretation, the glycan structures in Figs. 4 and 5 were numbed in the order of their presences, and those numbers are inconsistent with the labels of glycans in the library (Fig. S22).

### Synthesis of G1 standard glycans

Sialoglycoprotein (SGP) was isolated from commercial egg yolk^56^. Briefly, egg yolk isolated from 20 eggs was washed for 3 times with 400 mL of diethyl ether followed by vigorous stirring and filtration. SGP was extracted from the resulting solid using 400 mL of 40% acetone twice with stirring overnight. The supernatant was filtered, washed again with 200 mL of 40% acetone, evaporated and concentrated to afford an off-white solid. The solid was resuspended in water, and passed through a Buchner funnel containing 15 g of activated carbon, and washed with 2.5% acetonitrile. SGP was eluted with 25% acetonitrile containing 0.1% TFA. The eluted sialoglycans were subsequently lyophilized. Glycans were released from SGP using PNGase F and the resulting sialoglycans were desialylated using α2-3,6,8 neuraminidase. 20 mg of SGP solid was dissolved in 1.8 mL of water, followed by addition of 0.2 mL of 50 mM sodium acetate (NaOAc) containing 5 mM CaCl_2_ (pH 5.5). PNGase F (5 µL) and neuraminidase (2 µL) were then added and the mixture was incubated overnight at 37 °C. The reaction progress was monitored by high pH anionic chromatography-pulsed amperometric detection (HPAEC-PAD, Dionex IC-3000) using a Dionex CarboPac PA200 IC column (3 mm × 250 mm, 5.5 μm particle size) and a Gold Standard PAD waveform with a AgCl electrode. A LC gradient of 20%/0%/80% over 10 min to 60%/20%/20% of MP-A/MP-B/MP-C (MP, mobile phase; MP-A: 200 mM NaOH; MP-B: 150 mM NaOAc in 200 mM NaOH; MP-C: H_2_O) was used to separate glycans. G1(3) and G2 glycans eluted at 9.5 and 9.9 min, respectively. The glycans were further purified using HPLC equipped with a HyperCarb PGC column (Thermo Fisher, 10 mm × 150 mm, 5 µm particle size) and a LC gradient of 6.0% to 16.0% of MP-C: MP-D (MP-C: 95% acetonitrile in 4.9% water containing 0.1% TFA; MP-D: 0.1% TFA) at 5 mL min^−1^ over 41 min. The β- and α-anomers of G1(3) glycans eluted at 27.7 min and 33.6 min, respectively, while the G2 anomers eluted at 30.3 min and 35.9 min. Glycan compositions purified from each peak were confirmed by HPAEC-PAD analysis with commercially available G1 and G2 standards, and identified by LC MS/MS. To prepare G1(6) glycan, the purified G2 glycans (1.1 mg) were degalactosylated by dissolving in 200 µL of water, 30 µL of 0.5 M KCl and 30 µL of 50 mM NaOAc (pH 6.0). 30 µL of LacZ β-galactosidase was then added, and the solution was incubated at 30 °C. The reaction was completed in 42 h, as analyzed by HPAEC-PAD, resulting in 25% of G0 (completely degalactosylated) and 61% of a mixture of G1(6) and G1(3)). These products were then purified by HPLC using a HyperCarb PGC column (Thermo Fisher, 4.6 mm × 150 mm, 5 µm particle size) and a LC gradient of 4.0% to 13.7% MP A: MP B (MP A: 95% acetonitrile in 49% water containing 0.1% TFA; MP B: 0.1% TFA) at 2 mL min^−1^ over 41 min. The β- and α-anomers of G0 glycans eluted at 26.3 min and 33.0 min, respectively, while G1(6) anomers eluted at 30.0 min and 36.3 min and G1(3) anomers eluted at 31.0 and 37.5 min. Glycans were monitored at 214 nm using a UV detector, collected using a fraction collector and lyophilized. Purified glycans were confirmed by HPAEC-PAD analysis with commercially available G0 and G1 standards, and subsequent measurements by LC MS/MS.

## Supplementary Information

Supplementary Information.

## Data Availability

The datasets used and/or analyzed during the current study available from the corresponding authors on reasonable request.
