# Supplementary material for: Highly sensitive characterization of non-human glycan structures of monoclonal antibody drugs utilizing tandem mass spectrometry
Source: Sci Rep. 2022 Sep 6;12:15109. doi: 10.1038/s41598-022-19488-8 (PMC9448817; doi:10.1038/s41598-022-19488-8)
Supplement: Supplementary file 1 — Supplementary Information. [file 41598_2022_19488_MOESM1_ESM.pdf]

Supplementary Information for

Highly sensitive characterization of non-human glycan structures of monoclonal antibody drugs utilizing tandem mass spectrometry

Yi-Min She[1*], Shaojun Dai[2] and Roger Y. Tam[1*]

[1]Centre for Biologics Evaluation, Biologic and Radiopharmaceutical Drugs Directorate, Health Canada, Ottawa, Ontario K1A 0K9, Canada. [2]Development Center of Plant Germplasm Resources, College of Life Sciences, Shanghai Normal University, Shanghai 200234, China.

*Corresponding author: yi-min.she@hc-sc.gc.ca or roger.tam@hc-sc.gc.ca

Table of Contents

Figure S1 RP LC MS/MS analyses of tryptic glycopeptides of cetuximab…..…………………………...……Page S3

Figure S2 MS/MS identification of the typical sialoglycans and α-Gal containing glycans………………...…Page S4

Figure S3 EICs of sialoglycopeptides from a tryptic digest of trastuzumab ……..……..……….…….………Page S5

Figure S4 EICs of N-glycopeptides containing Man_{3-6}-based hybrid N-glycans of Infliximab…….........……Page S6

Figure S5 RP LC MS/MS analyses of the isobaric hybrid sialoglycopeptides……….………….…….….……Page S7

Figure S6 RP LC MS/MS analyses of isobaric biantennary α-Gal containing glycopeptides………..……..Page S8

Figure S7 RP LC MS/MS analyses of isobaric triantennary α-Gal containing glycopeptides………..……. Page S9

Figure S8 PGC LC chromatograms of the released native glycans from eight mAb drugs……..……..……...Page S10

Figure S9 PGC LC MS/MS analyses of the glycan standards…….……………………………………....…Page S11

Figure S10 PGC LC MS /MS analyses of known glycan isomers form fetuin and mAbs………………..….…Page S12

Figure S11 Predicted topological structures of glycan isomers……………………..…………….……………..Page S13

Figure S12 Comparison of EICs of glycopeptides and the glycans of mAbs …………..……………….…..…....Page S14

Figure S13 Identification of the isomers containing Man_3-based α-Gal glycans from mAbs ……..…………..Page S15

Figure S14 Exoglycosidase sequencing of glycan isomers containing Man_3-based α-Gal ..…………..…...……Page S16

Figure S15 Identification of the glycan isomers containing Man_4-based α-Gal ………….……….…..….…..Page S17

Figure S16 PGC LC MS/MS analyses of the biantennary α-Gal containing glycans ……………..…...….……Page S18

Figure S17 LC MS/MS analyses of the isobaric triantennary α-Gal containing glycopeptides ……..................Page S19

Figure S18 Identification of alpha-Gal containing glycan isomers by PGC LC MS/MS…….………….........Page S20

Figure S19 EICs of typical sialoglycans containing Neu5Gc released from the mAbs……………….….…...Page S21

Figure S20 Identification of Neu5Gc containing glycan isomers by PGC LC MS/MS.……..…………….…….Page S22

Figure S21 Distribution of non-human glycans in the mAbs derived from murine myeloma cells……….……Page S23

Figure S22 N-glycan library of monoclonal antibodies……………..….............……………………................Page S24

Table S1 Reversed-phase LC MS/MS identification of trastuzumab glycopeptides….……………….………..Page S27

Table S2 Reversed-phase LC MS/MS identification of rituximab glycopeptides……………….……........Page S30

Table S3 Reversed-phase LC MS/MS identification of infliximab glycopeptides ……………............…...Page S32

Table S4 Reversed-phase LC MS/MS identification of cetuximab glycopeptides ……….……........……Page S36

Table S5 Reversed-phase LC MS/MS identification of golimumab glycopeptides ………...…………......Page S40

Table S6 Peak areas of α-Gal containing N-glycopeptides …………………………………...…..…..…...Page S46

Table S7 PGC LC MS/MS analyses of golimumab glycans ……………………………………....…….Page S47

Table S8 PGC LC MS/MS analyses of cetuximab glycans………………………………..….……………...Page S50

Table S9 Predicted masses of mAb glycopeptides and glycans………………………….……………..….…….Page S54

Table S10 Distribution of isomeric non-human N-glycans of mAb drugs…………..……..………….……...Page S57

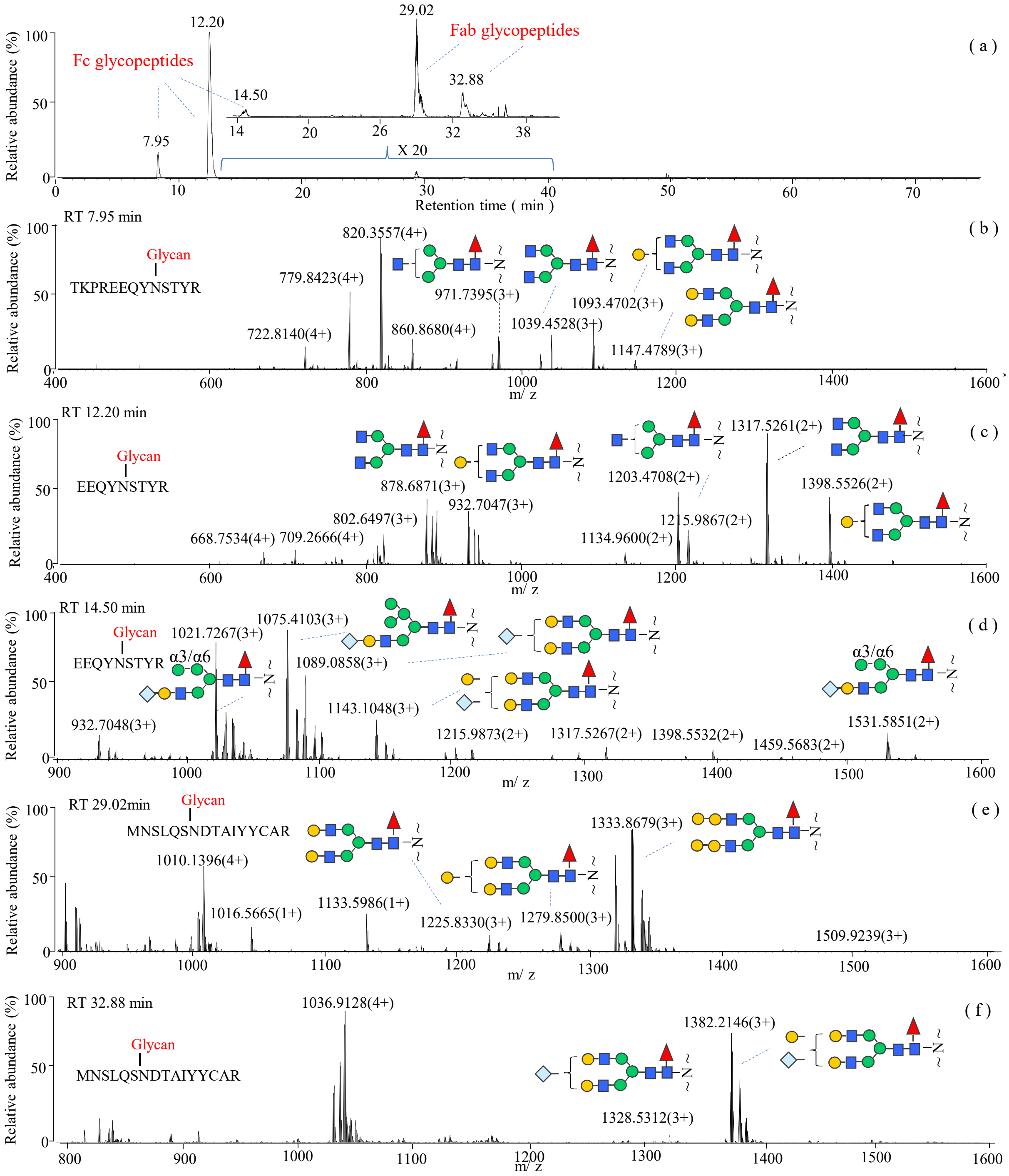

Figure S1. Reversed-phase LC MS/MS analyses of tryptic glycopeptides of cetuximab. Separated glycopeptides, based on the hydrophobic interaction of peptide sequences, are shown in the distinct regions. (a) Extracted ion chromatogram of the glycopeptides. (b-d) MS spectra of Fc glycopeptides at the retention times of 7.95 min, 12.20 min and 14.50 min, respectively. (e-f) MS spectra of Fab glycopeptides at 29.02 min and 32.88 min, respectively. Similar chromatographic profiles of Fc glycopeptides are observed in the tryptic digests of all mAbs examined.

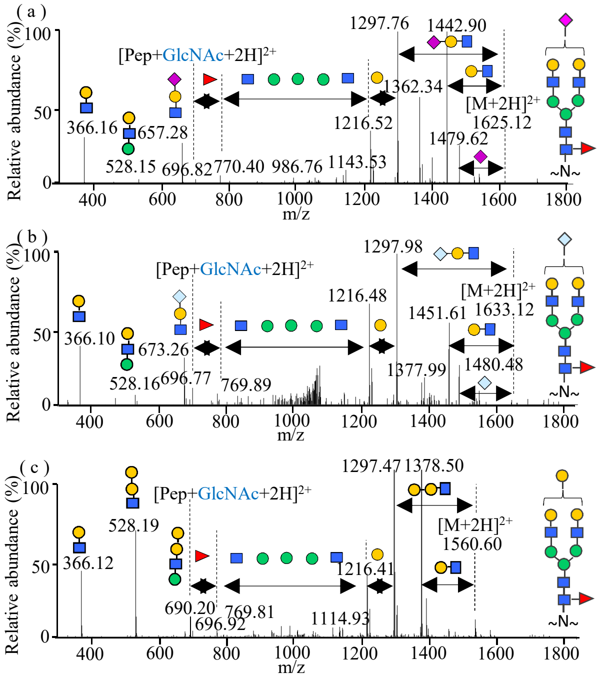

Figure S2. Identification of the typical sialoglycopeptides and the α-Gal containing glycopeptide by tandem mass spectrometry. MS/MS spectra of the triply charged ions of glycopeptides at residues EEQYNSTYR generated from a tryptic digest of cetuximab: (a) the precursor ion of *m/z* 1083.7537; (b) the precursor ion of *m/z* 1089.0885; (c) the precursor ion of *m/z* 1040.7393. The glycan structures containing terminal α-Gal, Neu5Ac and Neu5Gc are identified by the diagnostic ions and the complementary fragment ions with the losses of the branching side chains, respectively.

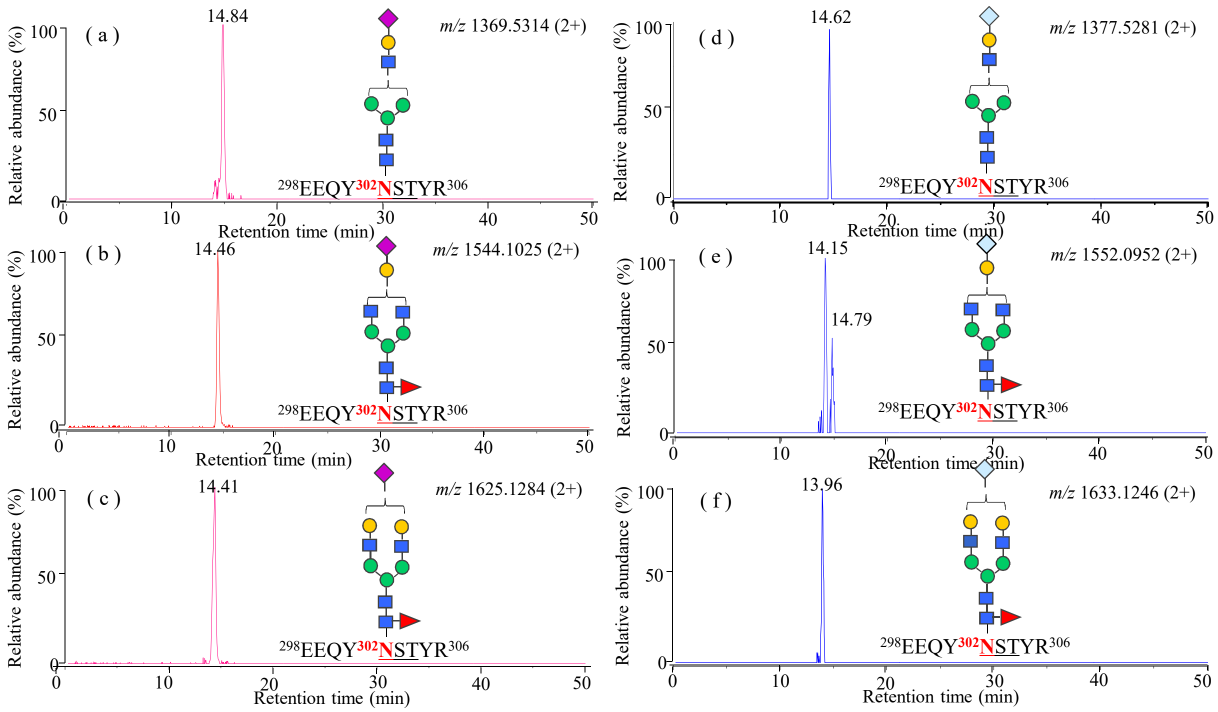

Figure S3. Extracted ion chromatograms of sialoglycopeptides from a tryptic digest of trastuzumab derived from CHO cells by reversed-phase LC MS/MS. (a-c) Neu5Ac-containing glycopeptides. (d-f) Neu5Gc-containing glycopeptides.

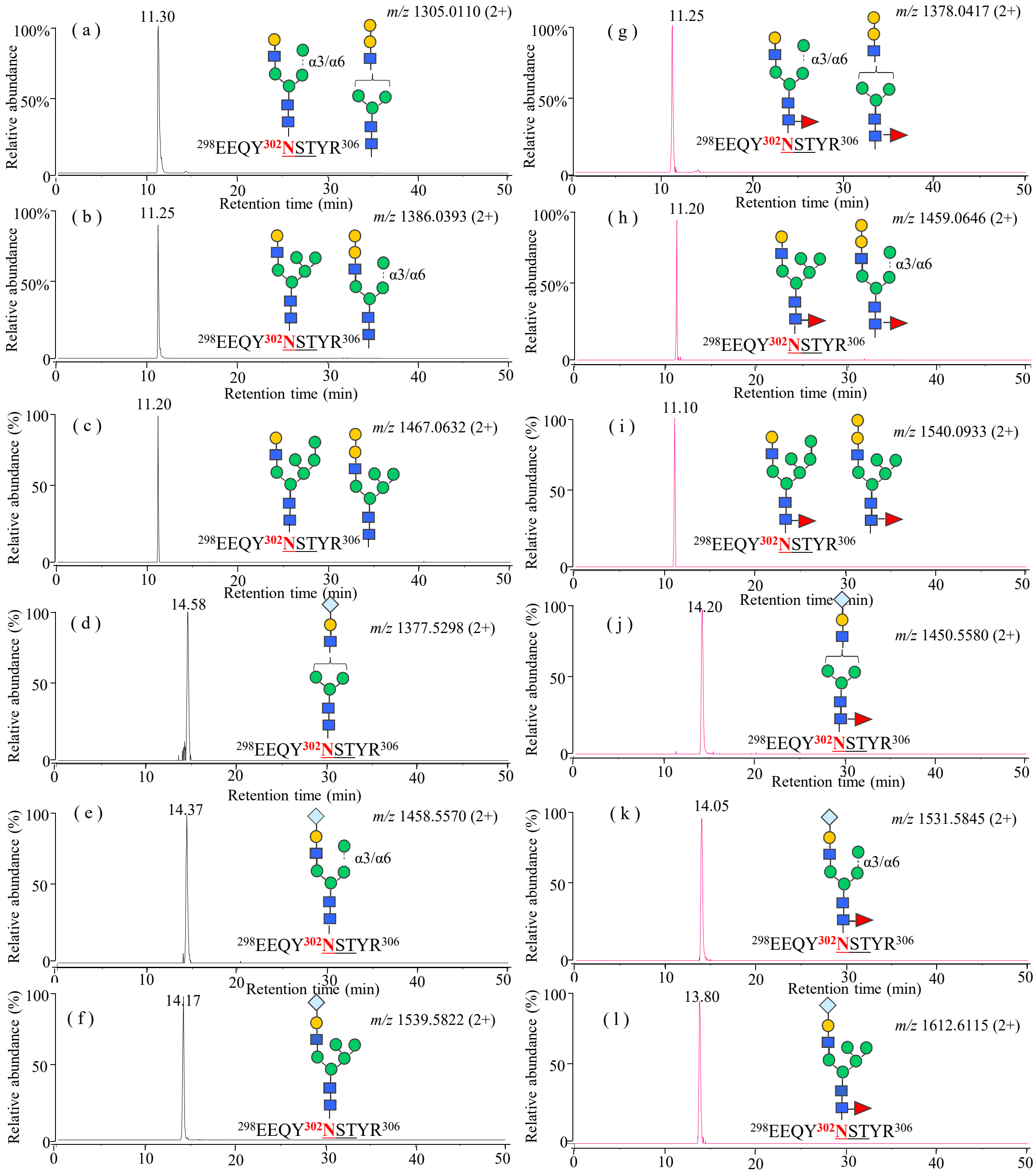

Figure S4. Extracted ion chromatograms of N-glycopeptides containing $Man_{3\text{-}6}$-based hybrid N-glycans or their isobaric α-Gal containing N-glycans in a tryptic digest of infliximab derived from murine myeloma cells. (a-f) afucosylated glycopeptides; (g-l) fucosylated glycopeptides. The presence of a single peak of glycopeptides indicates that the structural glycan isomers are not distinguishable by reversed-phase LC MS/MS.

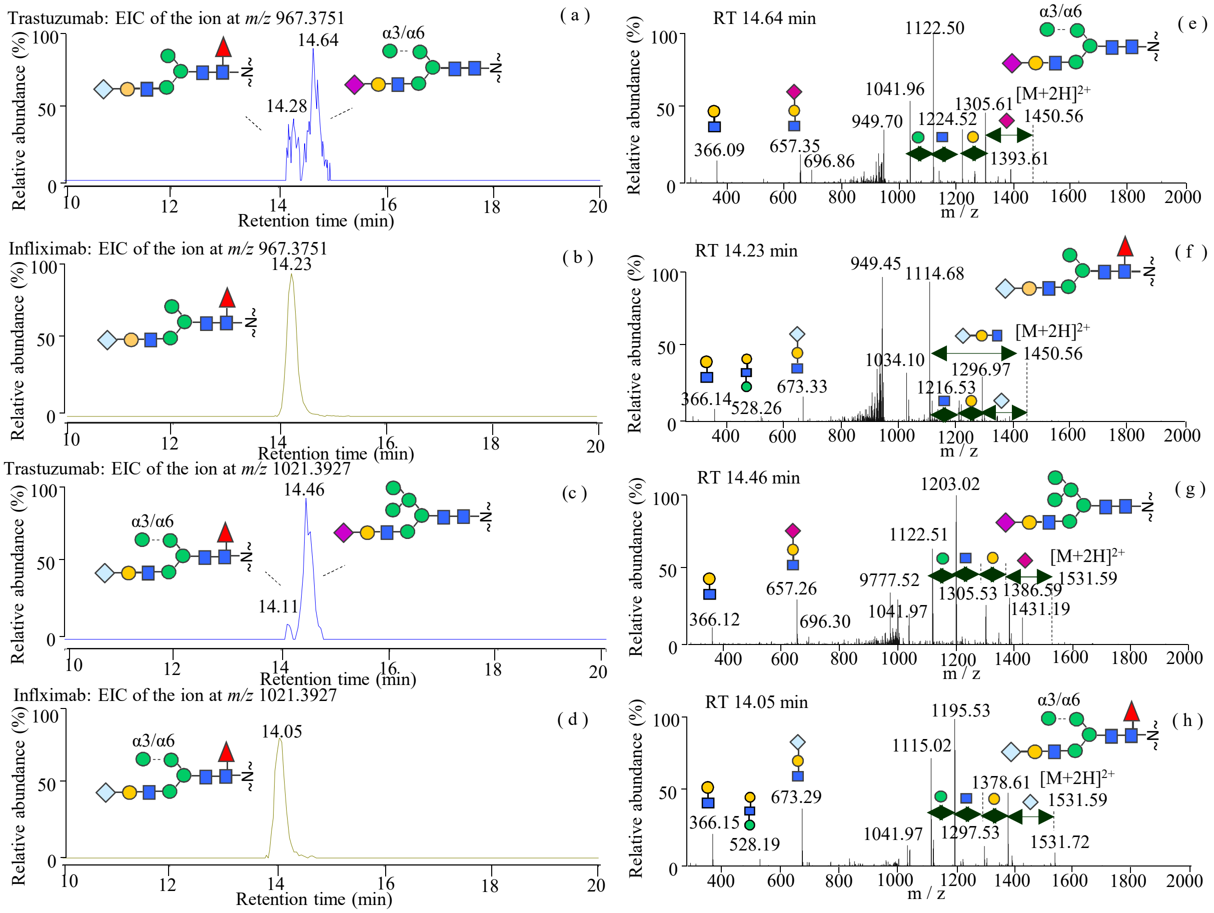

Figure S5. Reversed-phase LC MS/MS analyses of the isobaric hybrid sialoglycopeptides at residues EEQYNSTYR from tryptic digests of trastuzumab derived from CHO cells and infliximab derived from murine myeloma cells. (a-d) The extracted ion chromatograms (EICs) of the triply-charged ions at *m/z* 967.3751 and *m/z* 1021.3927. (e-h) MS/MS spectra of the corresponding glycopeptide ions at different retention times (RTs).

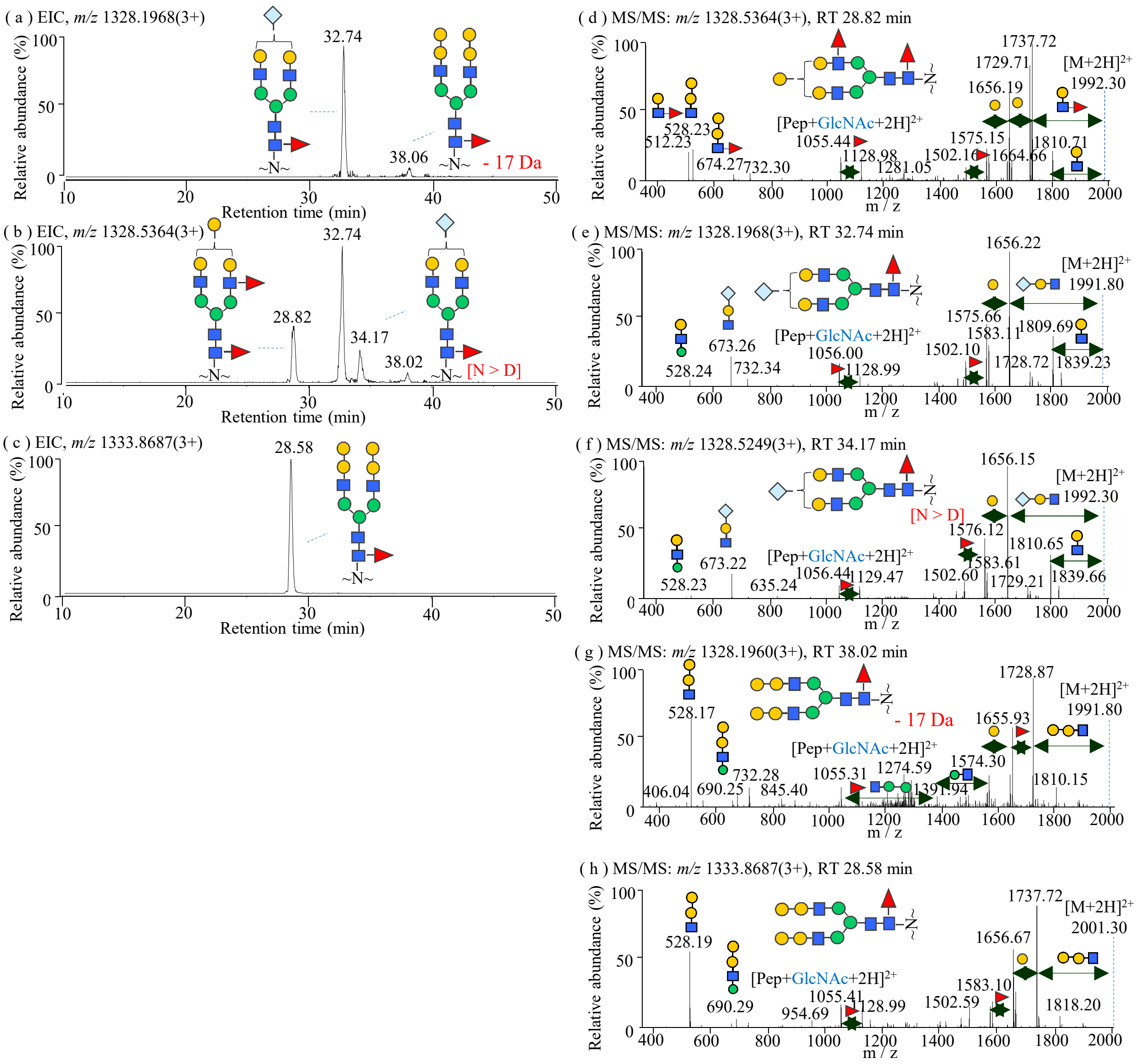

Figure S6. Reversed-phase LC MS/MS analyses of the isobaric biantennary α-Gal containing Fab glycopeptides from a tryptic digest of cetuximab. (a-c) The extracted ion chromatograms (EICs) of the triply-charged ions at *m/z* 1328.1968, *m/z* 1328.5364 and *m/z* 1333.8687. (d-h) MS/MS spectra of the corresponding glycopeptide ions at different retention times (RTs). The glycopeptide modifications were identified to have either the deamidation of an Asn to Asp (N > D) resulting in an increase of 1 Da in mass or the neutral loss of 17 Da molecule in the peptide regions.

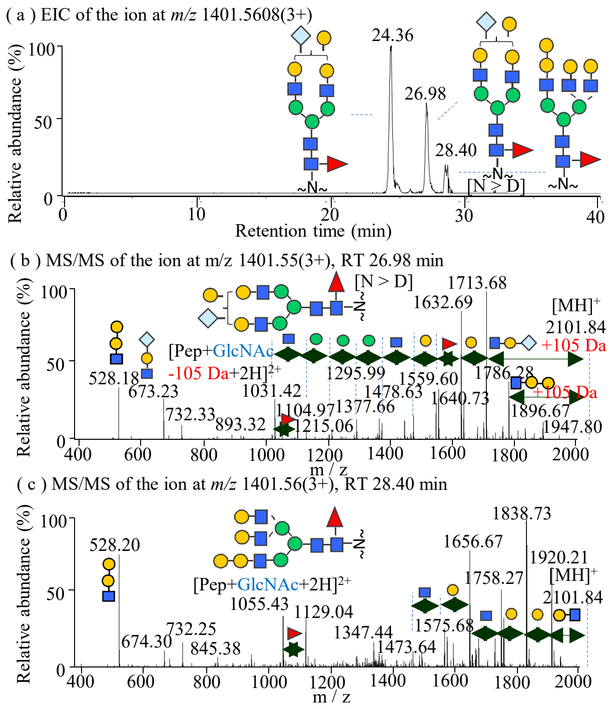

Figure S7. Reversed-phase LC MS/MS analyses of the isobaric triantennary α-Gal containing Fab glycopeptides from a tryptic digest of cetuximab. (a) Extracted ion chromatograms (EICs) of the triply-charged ions at m/z 1401.5608. (b-c) MS/MS spectra of the glycopeptide ion of m/z 1401.5608 at different retention times (RTs).

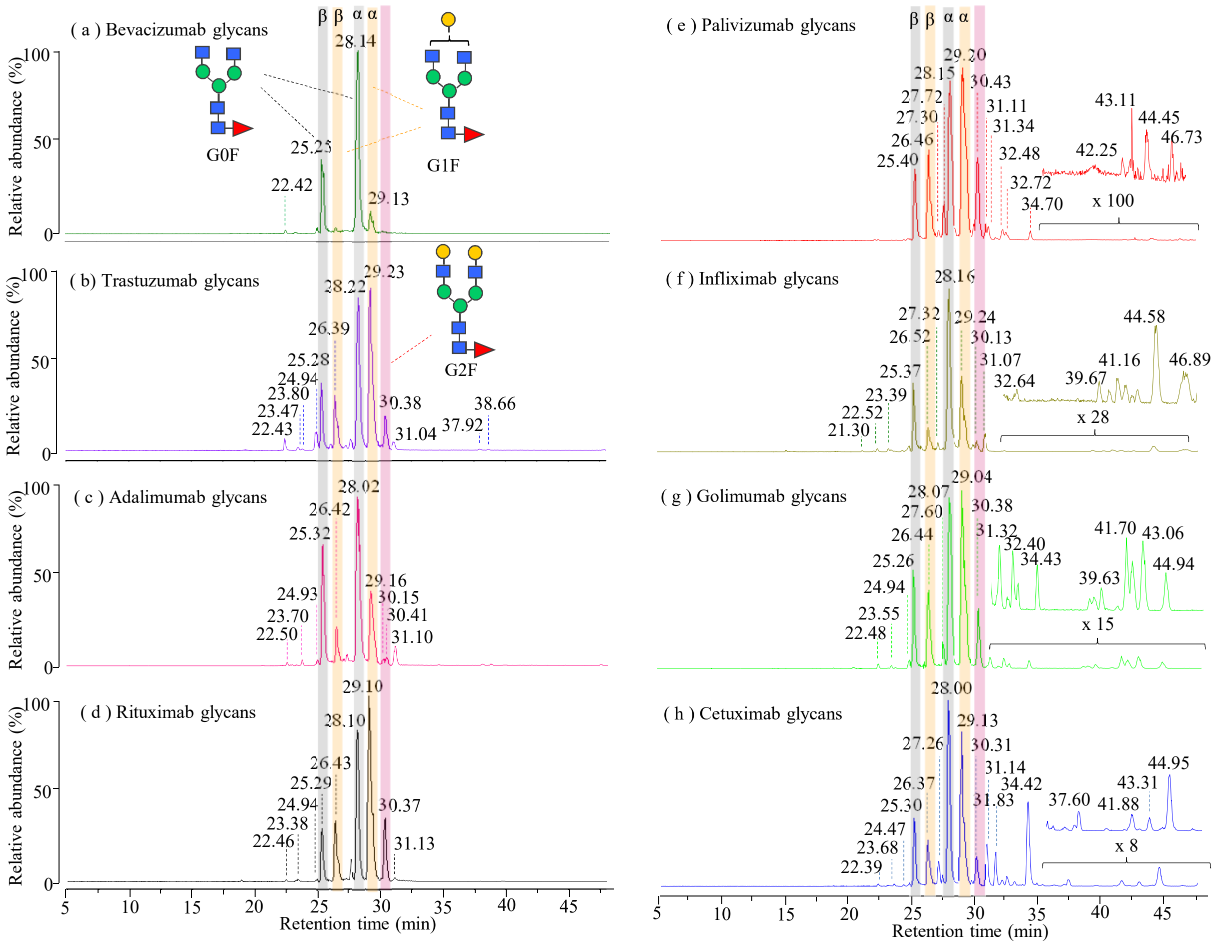

Figure S8. Base peak PGC LC MS/MS chromatograms of the released native glycans by endoglycosidase PNGase F from eight monoclonal antibody drugs. (a–d) Glycan profiles of mAbs expressed from Chinese Hamster Ovary (CHO) cells ; (e-h) glycan profiles of mAbs derived from murine myeloma (NS0, Sp2/0) cells. The high abundance peaks of agalactosylated, monogalactosylated and digalactosylated glycan (G0F, G1F, G2F) anomers are labelled with a consistent colour strip, respectively, and the chromatographic regions of sialoglycans containing Neu5Ac and Neu5Gc have been enlarged in the inset.

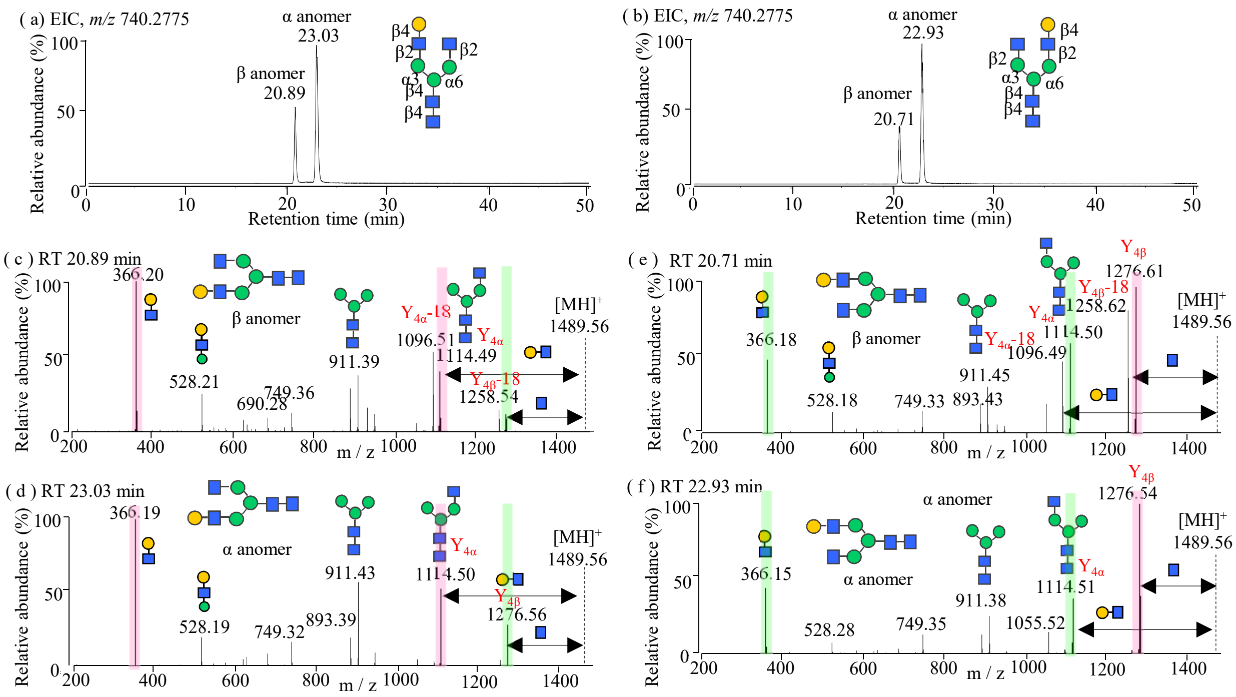

Figure S9. PGC LC MS/MS analysis of standard glycans. (a-b) The extracted ion chromatogram (EIC) of glycan standard G1 (α1-3) and G1 (α1-6) at the doubly charged ion of *m/z* 740.2775. (c-f) MS/MS spectra of the branch-specific glycan isomers of *m/z* 740.2775 at different retention times (RTs). The glycan G1 with a branched Gal-GlcNAc on the α1,6 antenna (*i.e.* G1(α1,6)) elutes faster than that on the α1,3 antenna (*i.e.* G1(α1,3)). A comparison of the relative intensities of ions shows a higher abundance of the fragments resulting from the branching side chain at the α1,3 antenna than those at the α1,6 antenna.

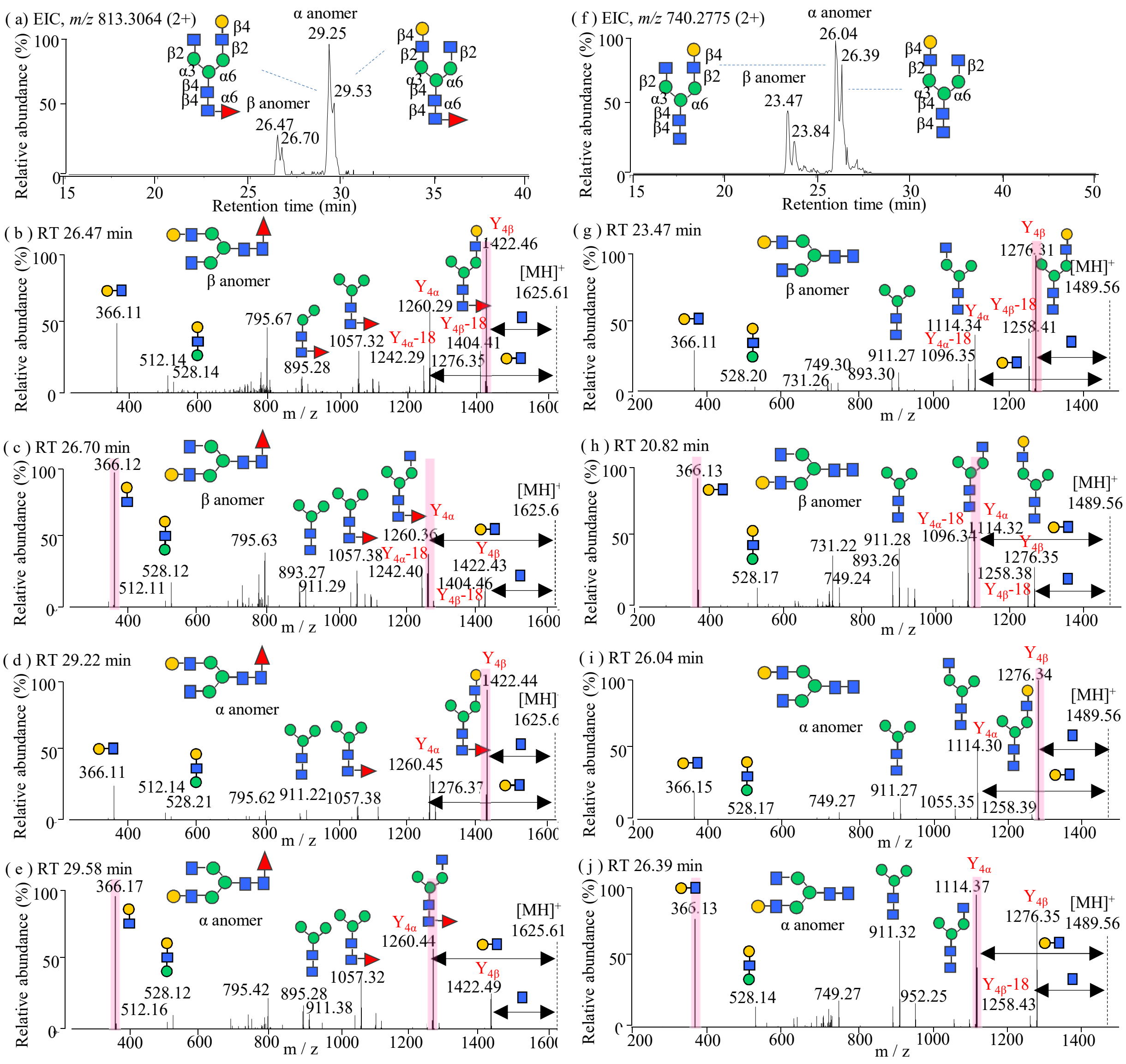

Figure S10. PGC LC MS/MS analyses of glycan isomers G1 and G1F of bovine fetuin and monoclonal antibodies. (a) The extracted ion chromatogram (EIC) of the fetuin glycan G1F at the doubly charged ion of *m/z* 813.3064. (b-e) MS/MS spectra of the branch-specific glycan isomers of *m/z* 813.30 at different retention times (RTs). (f) The EIC of the trastuzumab glycan G1 at the doubly charged ion of *m/z* 740.2775. (g-j) MS/MS spectra of the branch-specific glycan isomers of *m/z* 740.28 at different RTs. The glycan G1 on the α1,3 antenna branching structure (*i.e.* G1(α1,3)) elutes slower than that on the α1,6 antenna *(i.e.* G1(α1,6)). The relative abundances of the fragment ions shows an easier cleavage or neutral loss of the branching side chain at the α1,3 antenna than that at the α1,6 antenna, resulting in a higher intensity of the corresponding b and y fragments (highlighted in pink). The bovine fetuin was purchased from Sigma Co. (Catalog no. F2379).

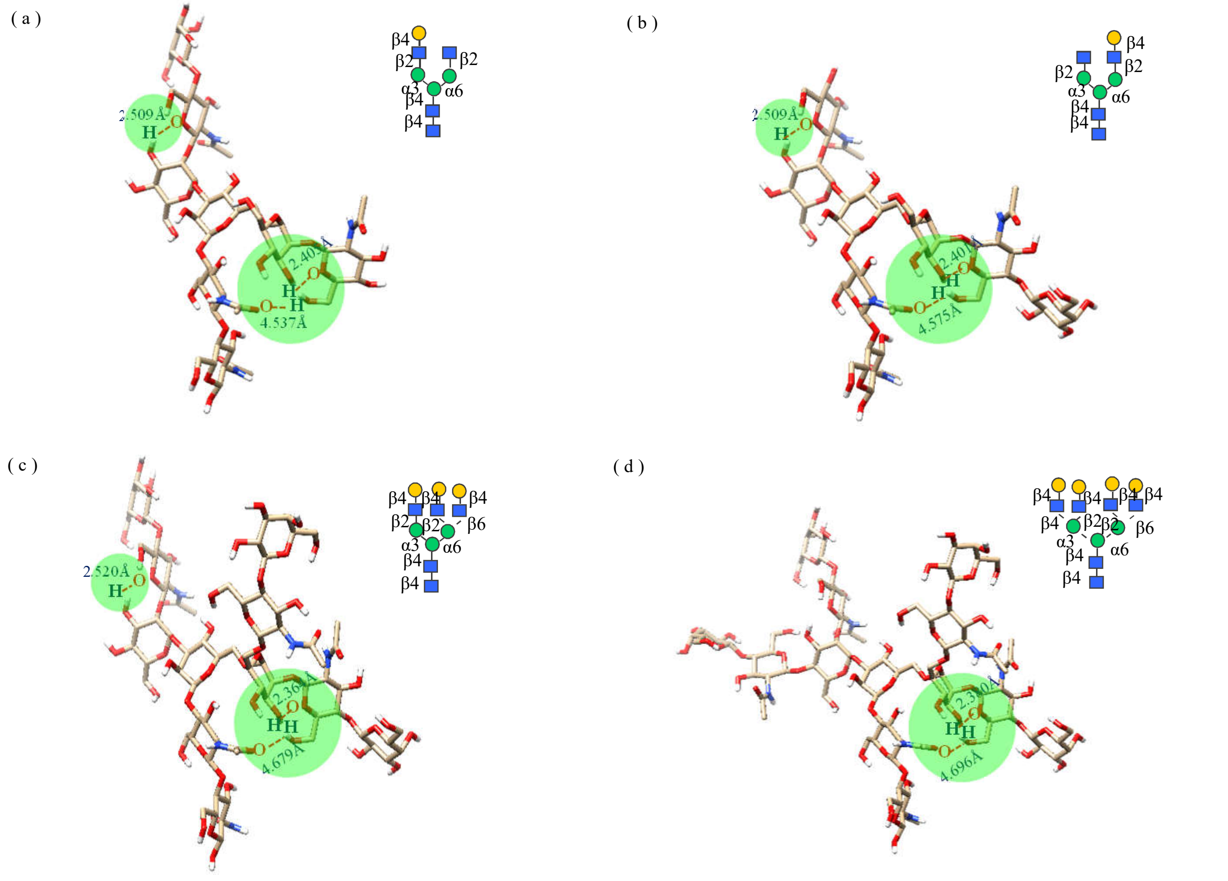

Figure S11. Predicted topological structures of glycan isomers**.** (a) Biantennary glycan G1(α1,3). (b) Biantennary glycan G1(α1,6). (c) Triantennary glycan. (d) Tetraantennary glycan. The 3D structural modeling is constructed by Glycam (www.glycam.org, Complex Carbohydrate Research Center, The University of Georgia), and the intramolecular hydrogen-bonding interactions are highlighted on the glycans.

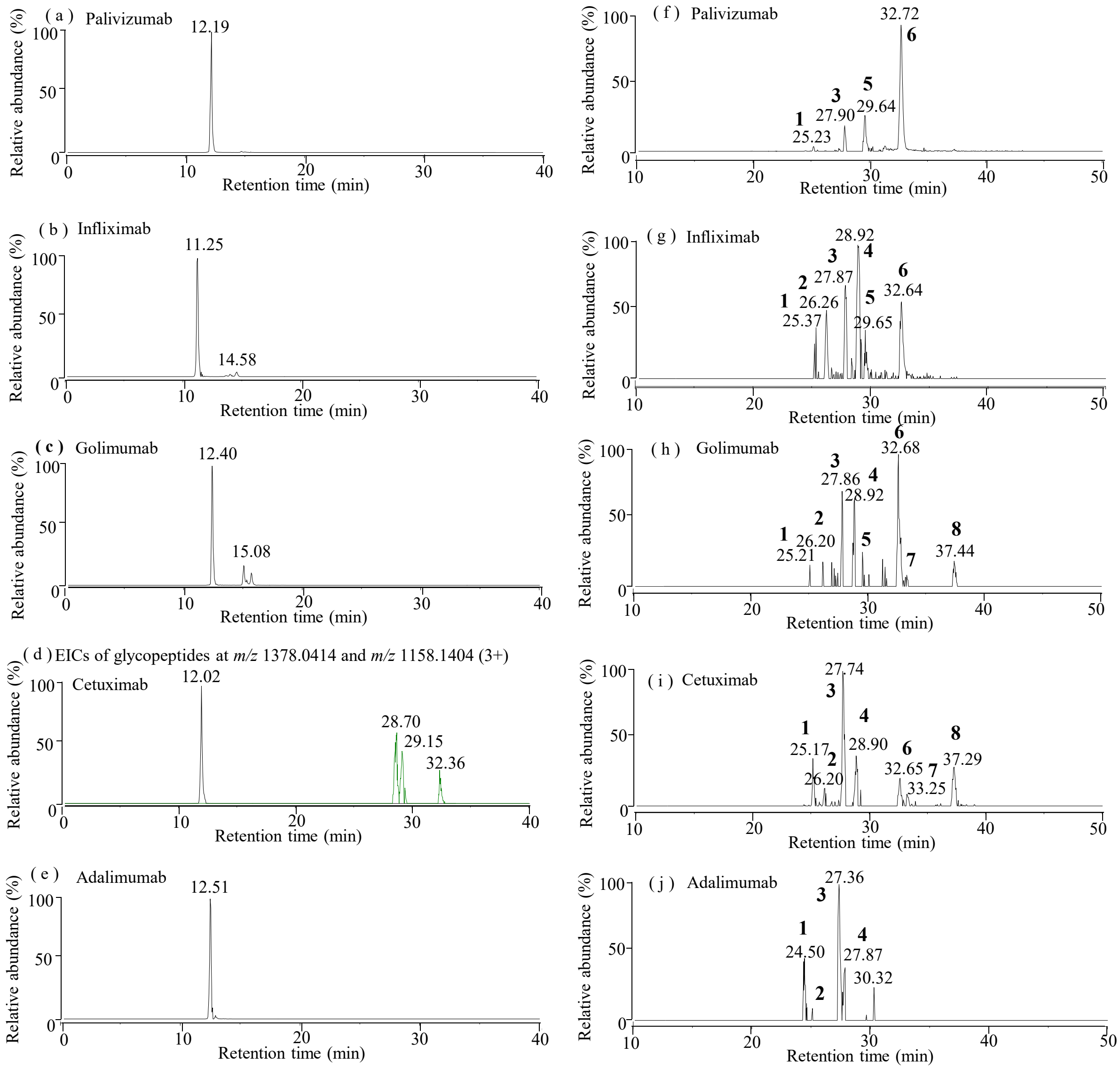

Figure S12. Parallel comparison of the extracted ion chromatograms (EICs) of tryptic glycopeptides and the released glycans of mAbs by PNGase F. LC MS/MS analyses of the Man_3-based α-Gal containing glycopeptides at the doubly charged ion of *m/z* 1378.0414 (residues EEQYNSTYR at the Fc region, black line), the triply-charged ion of *m/z* 1158.1404 (residues MNSLQSNDTAIYYCAR at the Fab region, dark green line) and the released glycans at the ion of *m/z* 792.7932 from palivizumab, infliximab, golimumab and cetuximab derived from murine myeloma NS0, Sp2/0 cells, and adalimumab as a representative mAb derived from CHO cells. (a-e) EICs of the glycopeptide ions at *m/z* 1378.0414 and *m/z* 1158.1404 separated by reversed-phase LC; (f-j) EICs of the released glycan isomers at *m/z* 792.7932 resolved by PGC. The labeled glycans **1-8** on chromatographic peaks were subsequently identified by MS/MS sequencing in Figure S13.

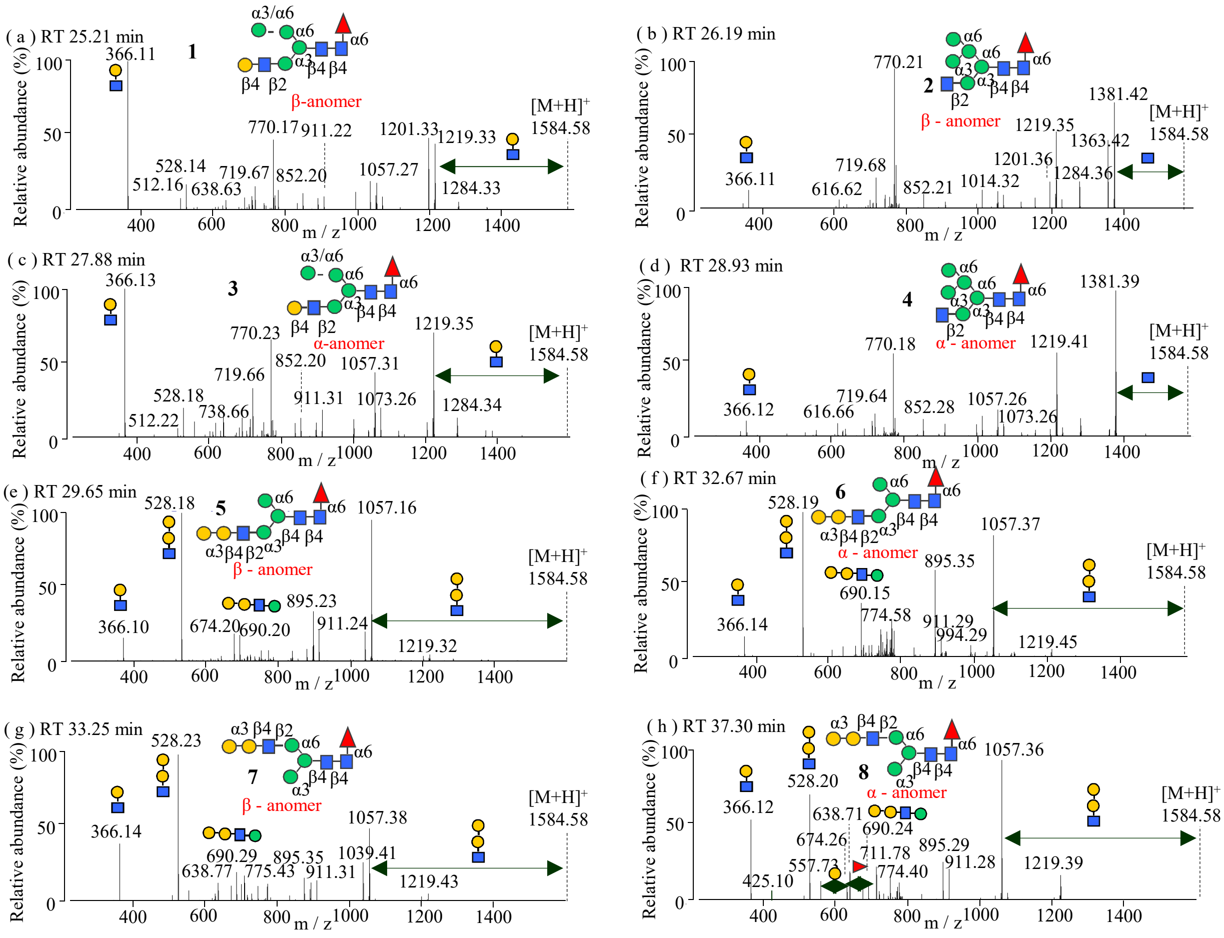

Figure S13. Identification of the structural isomers containing Man$_3$-based α-Gal containing glycans from mAbs derived from murine myeloma cells. (a-h) MS/MS spectra of the glycan ions at *m/z* 792.7932 at different retention times (RTs).

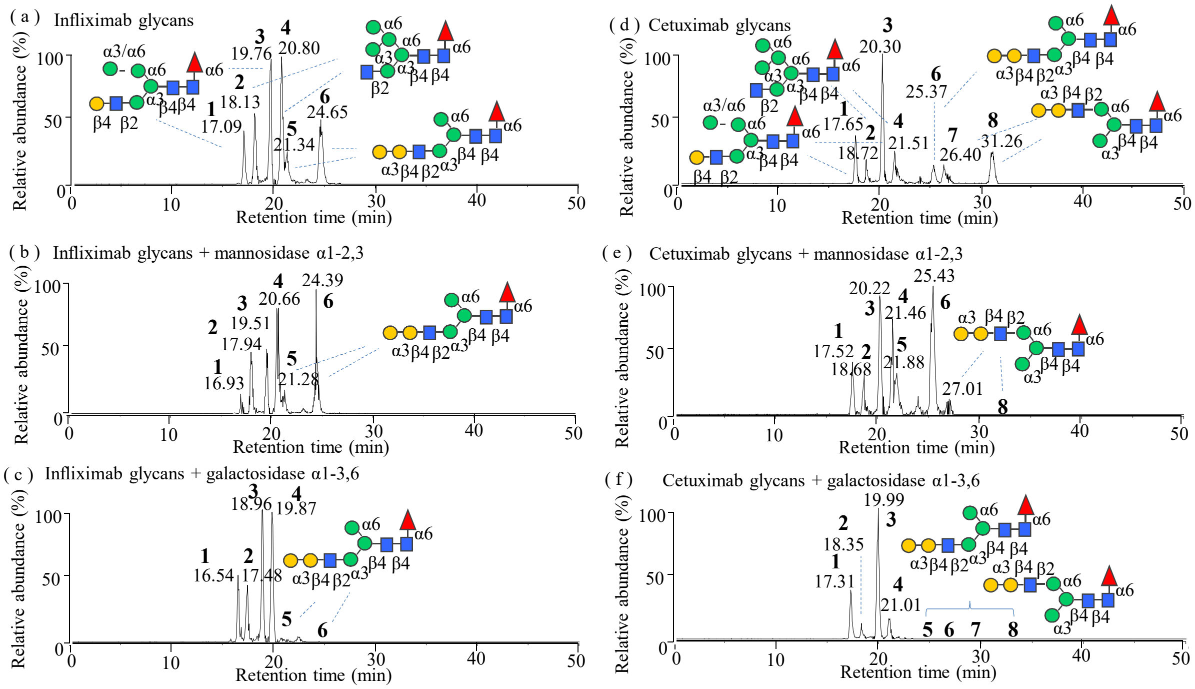

Figure S14. Exoglycosidase sequencing of glycan isomers containing Man_3-based α-Gal at *m/z* 792.7932 from infliximab and cetuximab derived from murine myeloma cells. PGC MS/MS analyses were conducted using Orbitrap Fusion coupled with an Acquity UPLC M-class system. (a-b, d-e) Extracted ion chromatograms (EICs) are shown an unbranched mannose in the α-1,3 antenna of isomers **7** and **8,** and α-1,6 antenna of isomers **1** and **3** by mannosidases α1-2,3, respectively. The presence of an increasing intensity of isomer **6** (EICs, b and f) might be derived from the cleaved products of the Man_4-based α-Gal-containing glycans in the glycan mixture; (c, f) The cleavage of isomers **5, 6, 7** and **8** by galactosidase α1-3,6 confirms the terminal α-Gal linkages of the glycans in the original structures.

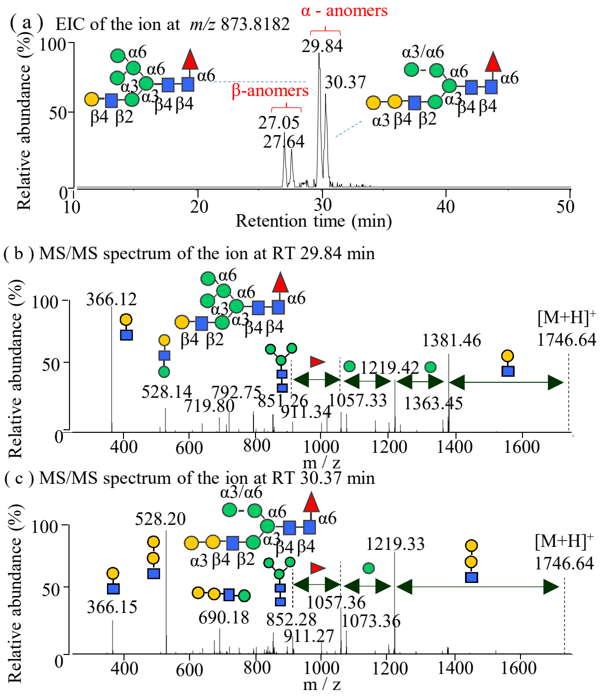

Figure S15. Identification of the structural isomers containing Man_4-based α-Gal containing glycans of mAbs derived from murine myeloma cells. (a) Extracted ion chromatogram (EIC) of the glycan at *m/z* 873.8182. (b) MS/MS fragmentation of the glycan ion at the retention time (RT) of 29.84 min. (c) MS/MS fragmentation of the glycan ion at the RT 30.37 min.

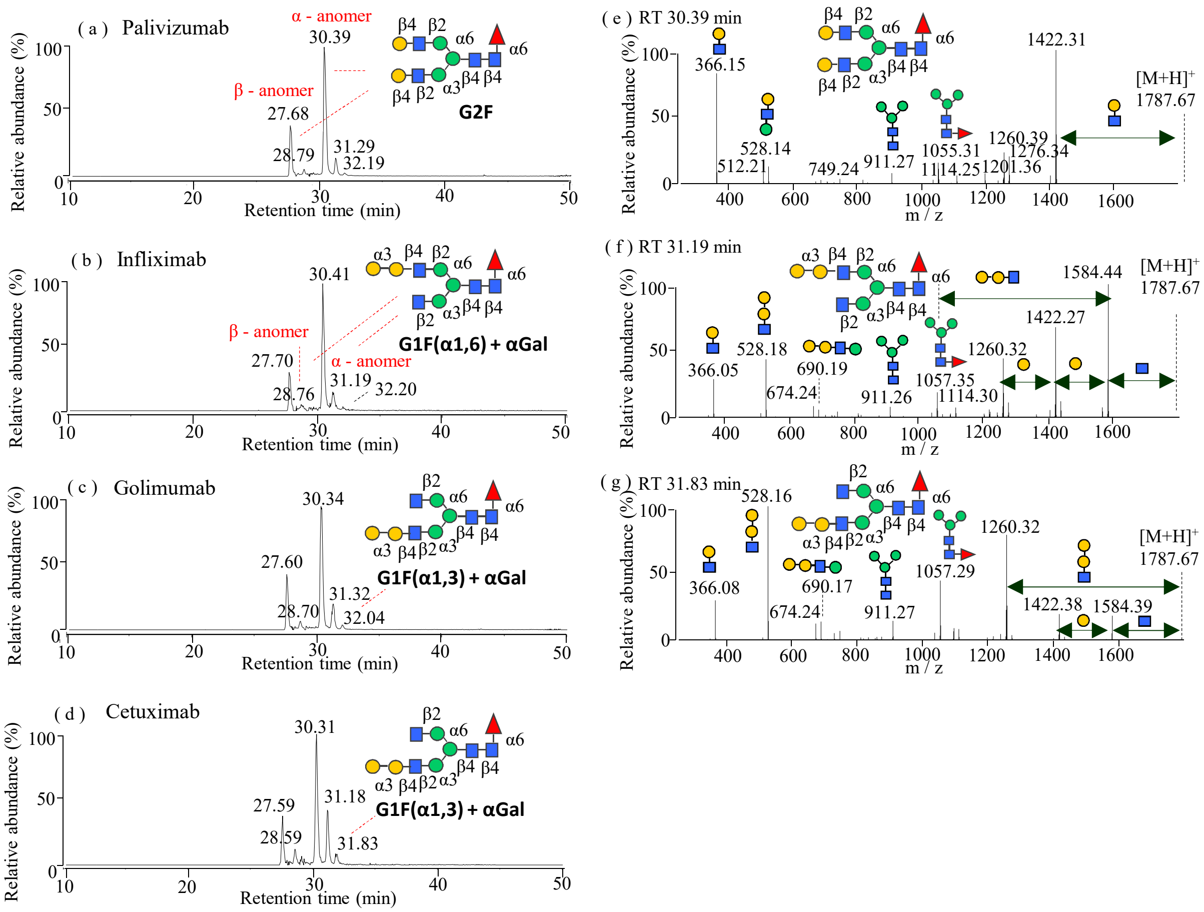

Figure S16. PGC LC MS/MS analyses of the biantennary α-Gal containing glycans at the doubly charged ion of *m/z* 894.3328 from palivizumab, infliximab, golimumab and cetuximab derived from murine myeloma NS0 and Sp2/0 cells. (a-d) The extracted ion chromatograms (EICs) of the ion at *m/z* 894.3328. (e-g) MS/MS spectra of the ions at different retention times (RTs).

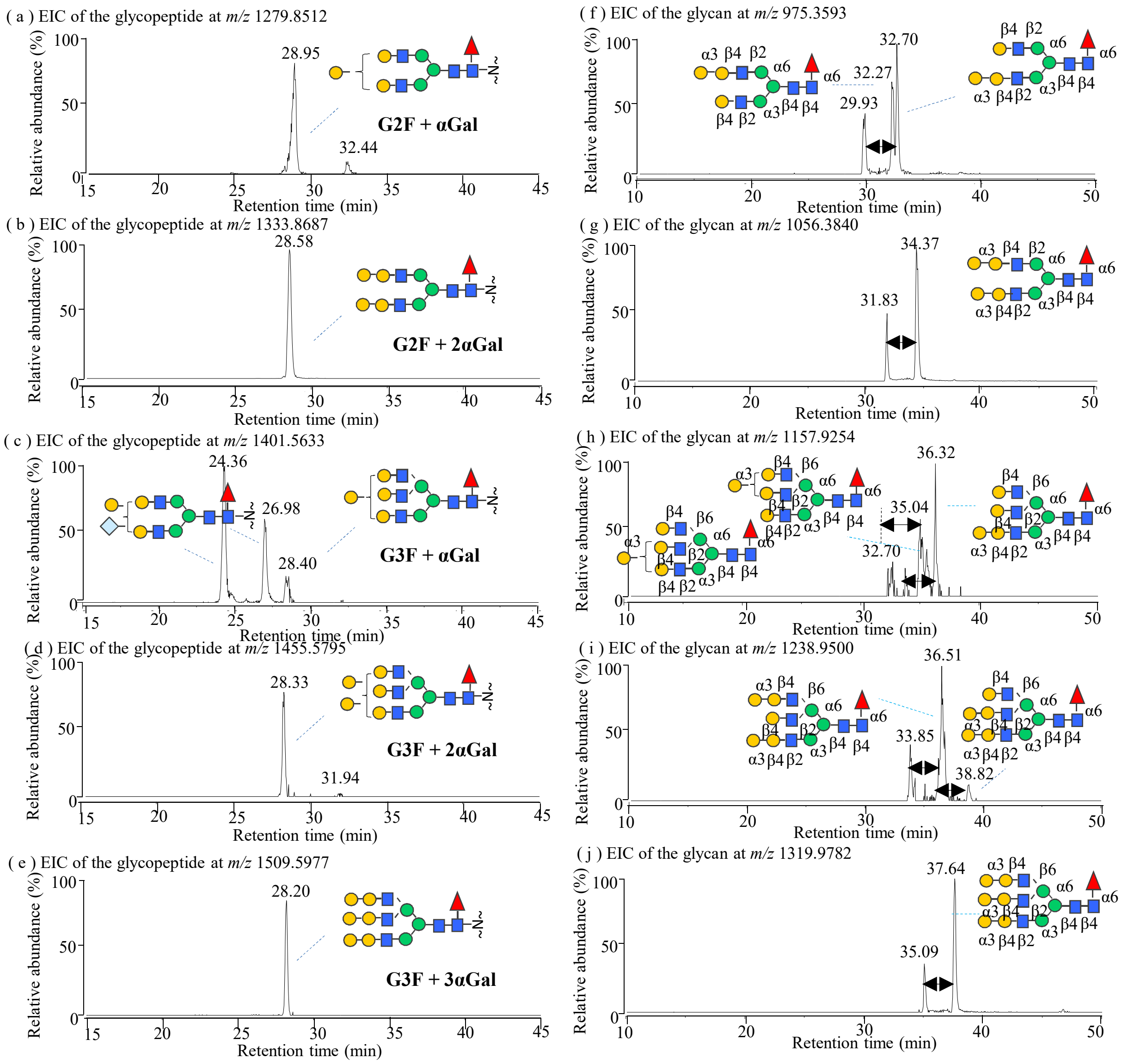

Figure S17. Comparison of LC MS/MS analyses of the triantennary α-Gal containing glycopeptides (residues MNSLQSNDTAIYYCAR) and the released glycans of Cetuximab derived from murine myeloma cells. (a-e) The extracted ion chromatograms (EICs) of the triply charged glycopeptide ions at *m/z* 1279.8512, *m/z* 1333.8687, *m/z* 1401.5633, *m/z* 1455.5795 and *m/z* 1509.5977, which are obtained by reversed-phase LC MS/MS. (f-j) EICs of the corresponding glycan ions separated by PGC LC MS/MS, respectively.

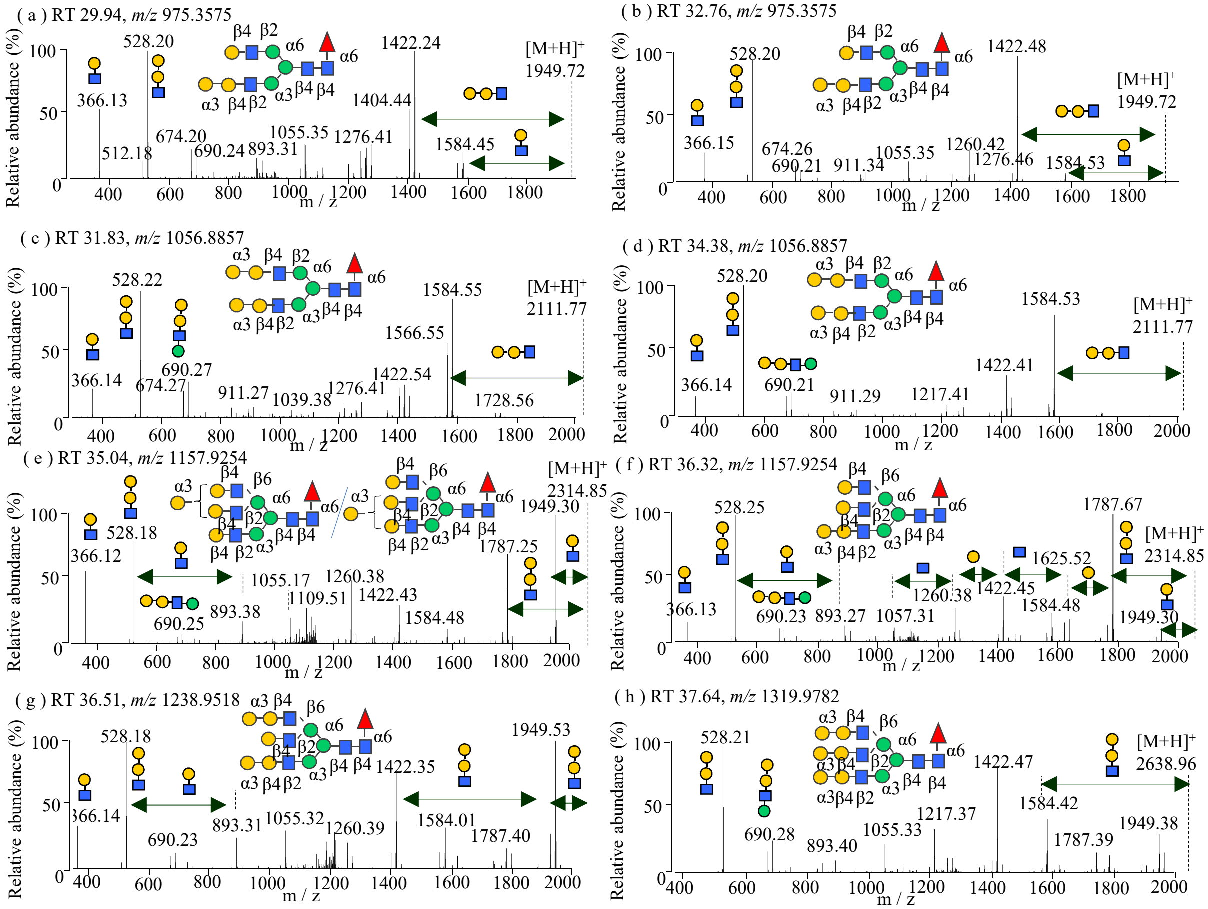

Figure S18. Identification of α-Gal containing glycan isomers by PGC LC MS/MS. (a, b) MS/MS spectra of the biantennary glycan G1F; (c, d) MS/MS spectra of the biantennary glycan G2F; (e, f) MS/MS spectra of the triantennary mono-α-Gal-containing glycan G3F; (g) MS/MS spectrum of the triantennary di-α-Gal-containing glycan G3F; (h) MS/MS spectrum of the triantennary tri-α-Gal-containing glycan G3F.

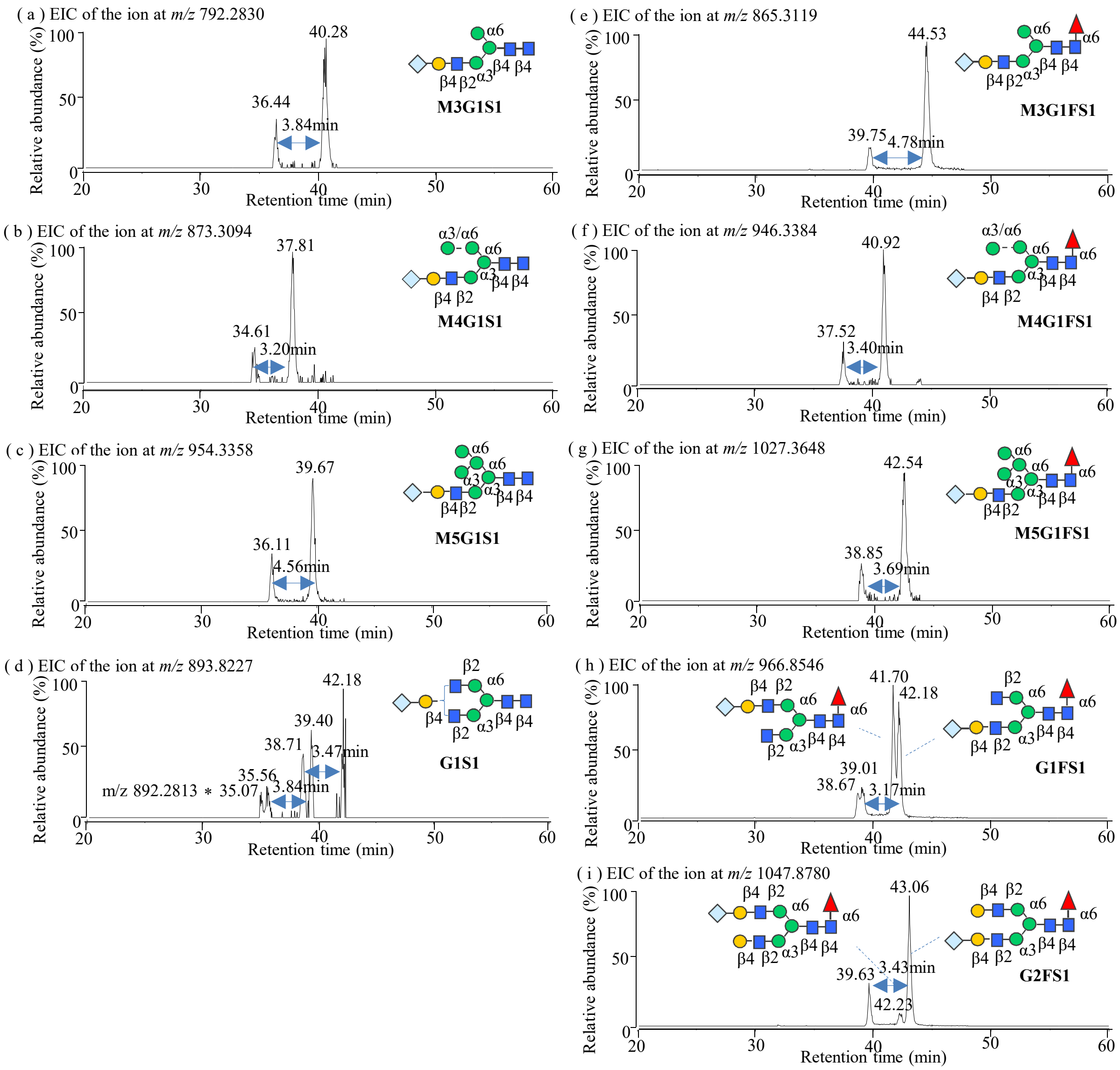

Figure S19. Extracted ion chromatograms (EICs) of the typical sialoglycans containing Neu5Gc released from mAbs of infliximab and golimumab. (a-d) Afucosylated Neu5Gc-sialoglycans. (e-i) Fucosylated Neu5Gc-sialoglycans.

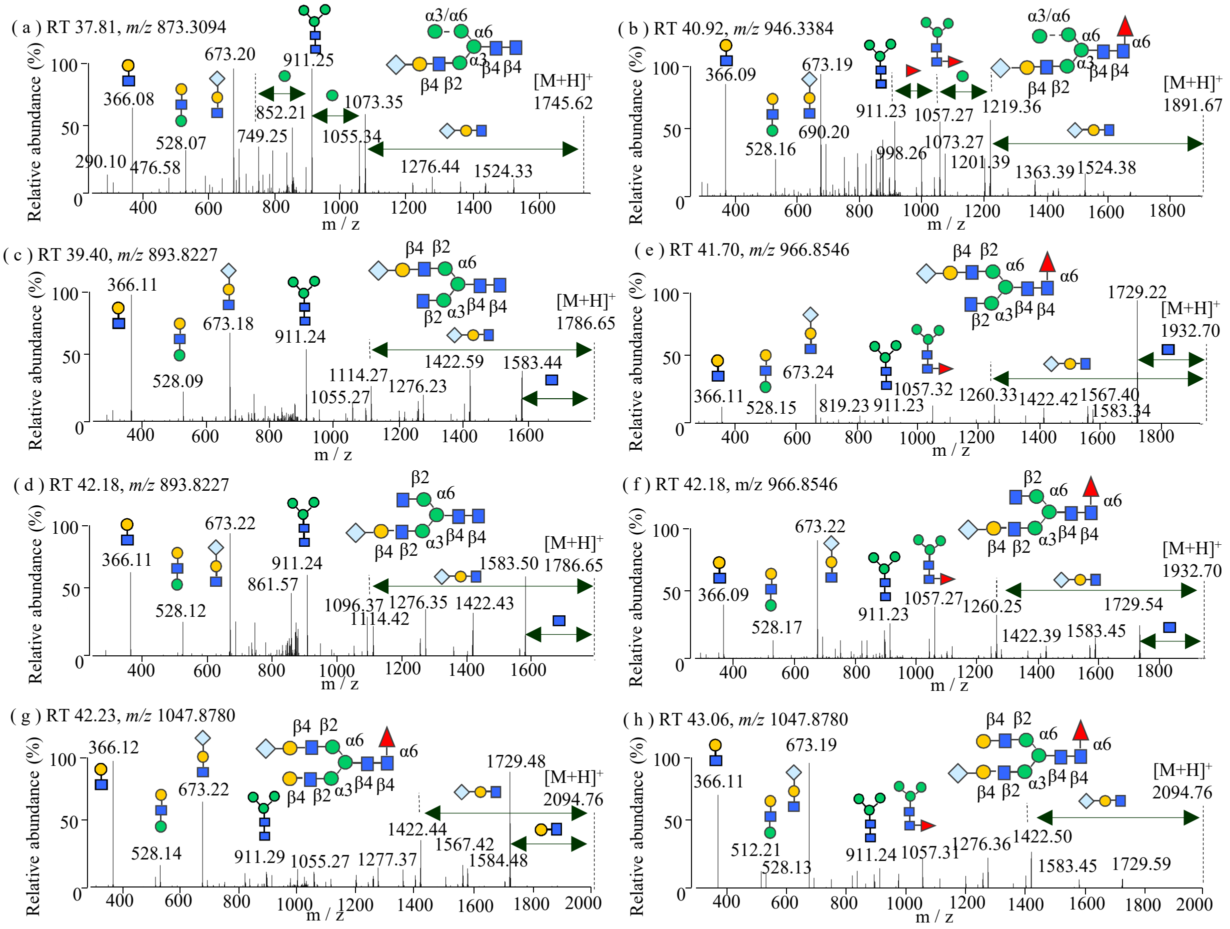

Figure S20. Identification of Neu5Gc-containing glycan isomers by PGC LC MS/MS. (a, b) MS/MS spectra of the Man_4-based hybrid Neu5Gc-containing glycans at different retention times (RTs); (c-h) MS/MS spectra of mono-Neu5Gc-sialylated complex glycans at different RTs.

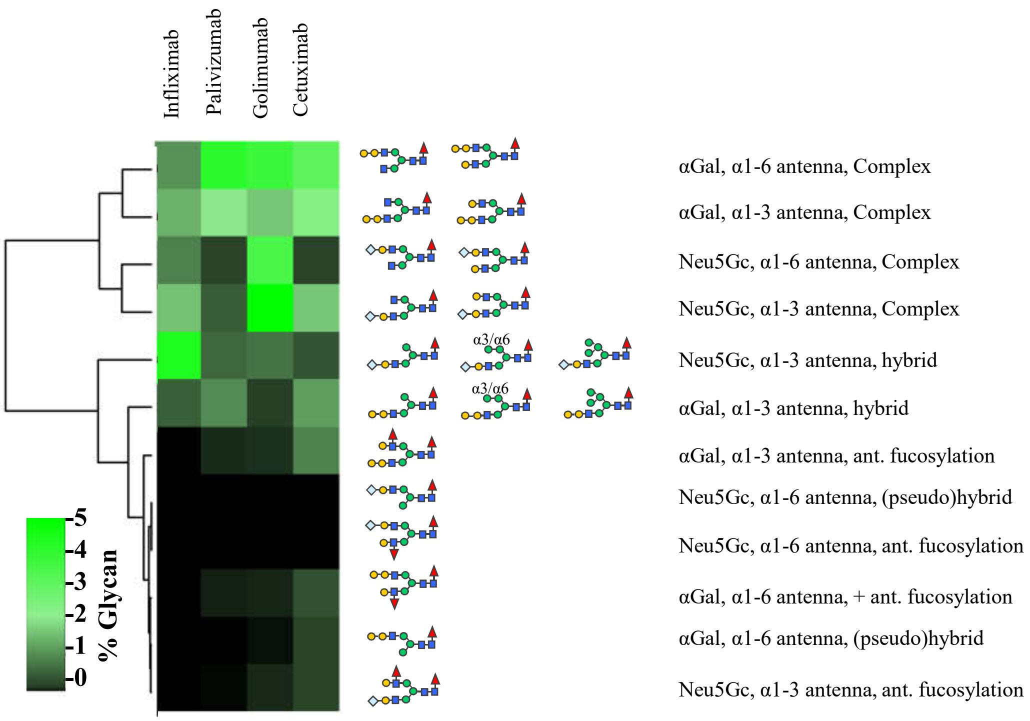

Figure S21. Distribution of non-human glycans in the mAbs derived from murine myeloma cells. Heatmap of α-Gal and Neu5Gc containing glycan isomers is built based on the percentage of the glycans in the total glycan composition (human compatible glycans + non-human glycans). The isomeric glycan structures are identified by PGC LC MS/MS analyses, and the peak areas of the extracted ion chromatogram are measured by Thermo Xcalibur 4.1 software (Table S10). Hierarchical clustering of glycan groups is performed using R software (Ward's Method).

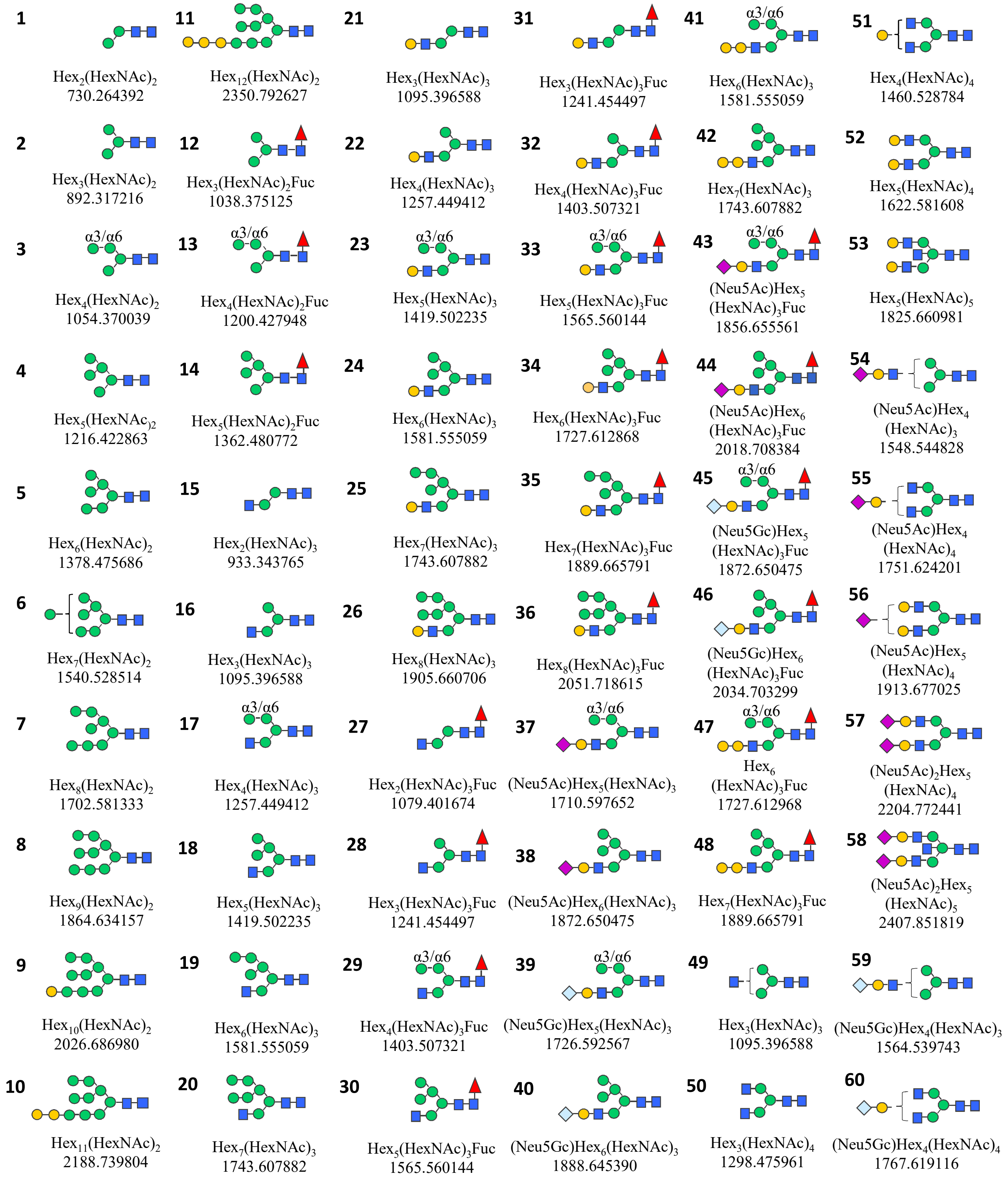

Figure S22. N-glycan library of monoclonal antibodies. The compositions and residue masses are shown under the glycan structures

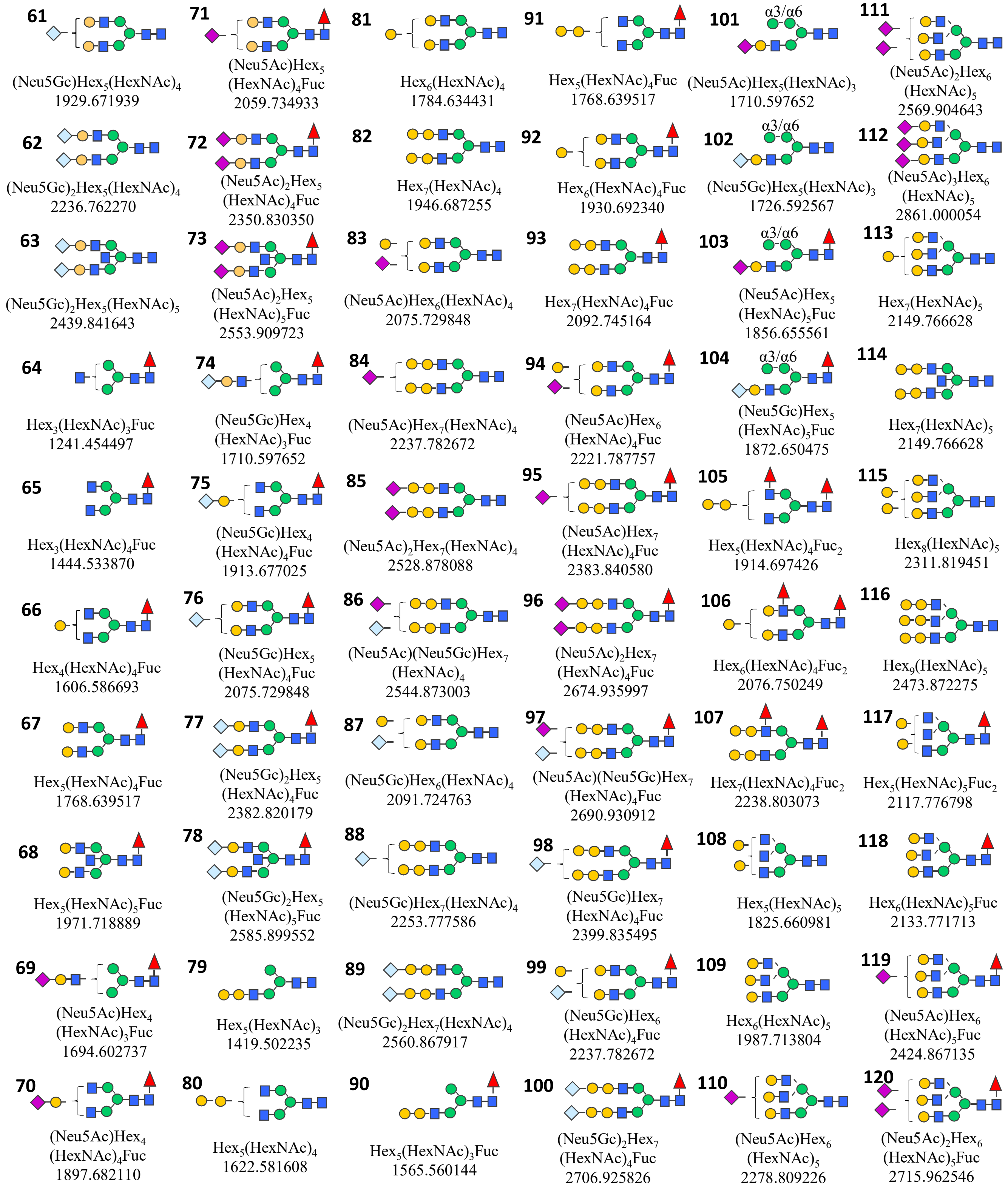

Figure S22. N-glycan library of monoclonal antibodies. The compositions and residue masses are shown under the glycan structures

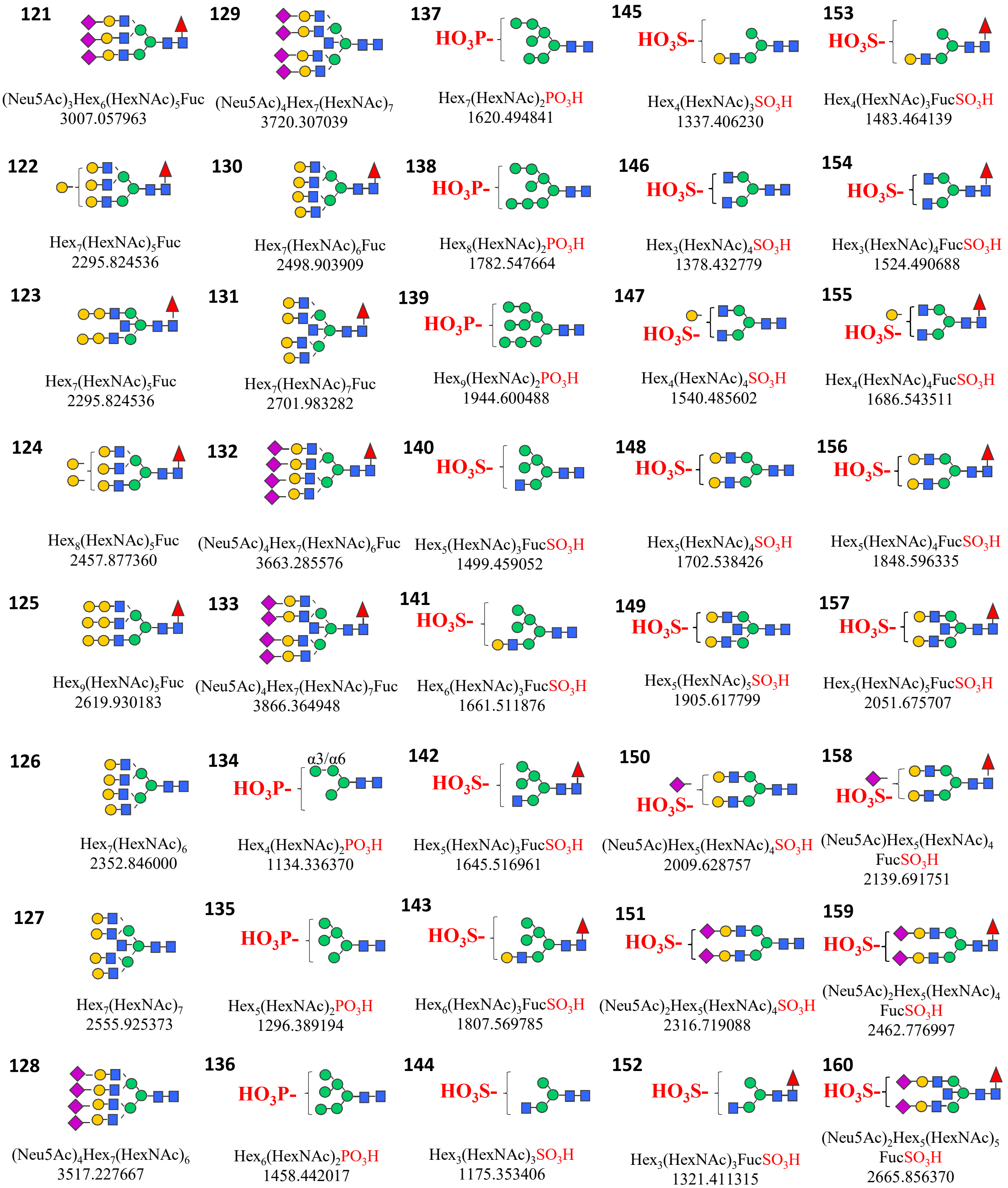

Figure S22. N-glycan library of monoclonal antibodies. The compositions and residue masses are shown under the glycan structures

Table S1. Identification of the tryptic glycopeptides of trastuzumab derived from CHO cells by reversed-phase LC MS/MS

Glycopeptide * *m/z* (charge)	Meas. MH+	Calc. MH+	ppm	Peptide MH+	Peptide sequence §	Glycan mass	Glycan composition	Putative glycan structure #
641.7867(4+)	2564.1233	2564.1257	-1	1671.8085	K.^{292}TKPREEQY^{300}NSTYR^{304}.V	892.3172	Man$_3$(GlcNAc)$_2$	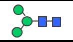
855.3793(3+)	2564.1223	2564.1257	-1	1671.8085	K.^{292}TKPREEQY^{300}NSTYR^{304}.V	892.3172	Man$_3$(GlcNAc)$_2$	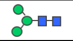
682.2995(4+)	2726.1745	2726.1785	-2	1671.8085	K.^{292}TKPREEQY^{300}NSTYR^{304}.V	1054.3700	Man$_4$(GlcNAc)$_2$	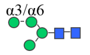
909.3971(3+)	2726.1757	2726.1785	-1	1671.8085	K.^{292}TKPREEQY^{300}NSTYR^{304}.V	1054.3700	Man$_4$(GlcNAc)$_2$	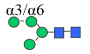
722.8135(4+)	2888.2305	2888.2314	0	1671.8085	K.^{292}TKPREEQY^{300}NSTYR^{304}.V	1216.4229	Man$_5$(GlcNAc)$_2$	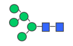
963.4149(3+)	2888.2291	2888.2314	-1	1671.8085	K.^{292}TKPREEQY^{300}NSTYR^{304}.V	1216.4229	Man$_5$(GlcNAc)$_2$	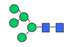
1017.4321(3+)	3050.2807	3050.2842	-1	1671.8085	K.^{292}TKPREEQY^{300}NSTYR^{304}.V	1378.4757	Man$_6$(GlcNAc)$_2$	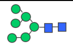
692.5560 (4+)	2767.2005	2767.2051	-2	1671.8085	K.^{292}TKPREEQY^{300}NSTYR^{304}.V	1095.3966	(GlcNAc)Man$_3$(GlcNAc)$_2$	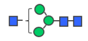
923.0724(3+)	2767.2016	2767.2051	-1	1671.8085	K.^{292}TKPREEQY^{300}NSTYR^{304}.V	1095.3966	(GlcNAc)Man$_3$(GlcNAc)$_2$	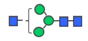
743.3263(4+)	2970.2817	2970.2845	-1	1671.8085	K.^{292}TKPREEQY^{300}NSTYR^{304}.V	1298.4760	(GlcNAc)$_2$Man$_3$(GlcNAc)$_2$	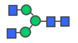
990.7654(3+)	2970.2806	2970.2845	-1	1671.8085	K.^{292}TKPREEQY^{300}NSTYR^{304}.V	1298.4760	(GlcNAc)$_2$Man$_3$(GlcNAc)$_2$	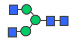
1044.7825(3+)	3132.3319	3132.3373	-2	1671.8085	K.^{292}TKPREEQY^{300}NSTYR^{304}.V	1460.5288	Gal(GlcNAc)$_2$Man$_3$(GlcNAc)$_2$	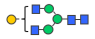
1025.7764(3+)	3075.3136	3075.3158	-1	1671.8085	K.^{292}TKPREEQY^{300}NSTYR^{304}.V	1403.5073	Gal(GlcNAc)Man$_3$(GlcNAc)$_2$Fuc	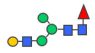
850.6113(4+)	3399.4217	3399.4214	0	1671.8085	K.^{292}TKPREEQY^{300}NSTYR^{304}.V	1727.6129	Gal(GlcNAc)Man$_5$(GlcNAc)$_2$Fuc	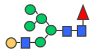
917.7408(3+)	2751.2068	2751.2100	-1	1671.8085	K.^{292}TKPREEQY^{300}NSTYR^{304}.V	1079.4015	(GlcNAc)Man$_2$(GlcNAc)$_2$Fuc	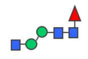
971.7584(3+)	2913.2596	2913.2630	-1	1671.8085	K.^{292}TKPREEQY^{300}NSTYR^{304}.V	1241.4545	(GlcNAc)Man$_3$(GlcNAc)$_2$Fuc	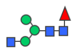
779.8412(4+)	3116.3413	3116.3424	0	1671.8085	K.^{292}TKPREEQY^{300}NSTYR^{304}.V	1444.5339	(GlcNAc)$_2$Man$_3$(GlcNAc)$_2$Fuc	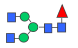
1039.4517(3+)	3116.3395	3116.3424	-1	1671.8085	K.^{292}TKPREEQY^{300}NSTYR^{304}.V	1444.5339	(GlcNAc)$_2$Man$_3$(GlcNAc)$_2$Fuc	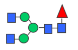
1025.7766(3+)	3075.3142	3075.3158	-1	1671.8085	K.^{292}TKPREEQY^{300}NSTYR^{304}.V	1403.5073	Gal(GlcNAc)Man$_3$(GlcNAc)$_2$Fuc	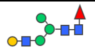
820.3549(4+)	3278.3961	3278.3952	0	1671.8085	K.^{292}TKPREEQY^{300}NSTYR^{304}.V	1606.5867	Gal(GlcNAc)$_2$Man$_3$(GlcNAc)$_2$Fuc	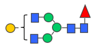
1093.4696(3+)	3278.3932	3278.3952	-1	1671.8085	K.^{292}TKPREEQY^{300}NSTYR^{304}.V	1606.5867	Gal(GlcNAc)$_2$Man$_3$(GlcNAc)$_2$Fuc	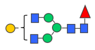
860.8676(4+)	3440.4469	3440.4480	0	1671.8085	K.^{292}TKPREEQY^{300}NSTYR^{304}.V	1768.6395	Gal$_2$(GlcNAc)$_2$Man$_3$(GlcNAc)$_2$Fuc	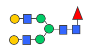
1147.4879(3+)	3440.4481	3440.4480	0	1671.8085	K.^{292}TKPREEQY^{300}NSTYR^{304}.V	1768.6395	Gal$_2$(GlcNAc)$_2$Man$_3$(GlcNAc)$_2$Fuc	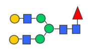
805.8437(4+)	3220.3513	3220.3533	-1	1671.8085	K.^{292}TKPREEQY^{300}NSTYR^{304}.V	1548.5448	(Neu5Ac)Gal(GlcNAc)Man$_3$(GlcNAc)$_2$	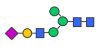
1074.1224(3+)	3220.3516	3220.3533	-1	1671.8085	K.^{292}TKPREEQY^{300}NSTYR^{304}.V	1548.5448	(Neu5Ac)Gal(GlcNAc)Man$_3$(GlcNAc)$_2$	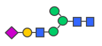
1128.1396(3+)	3382.4032	3382.4062	-1	1671.8085	K.^{292}TKPREEQY^{300}NSTYR^{304}.V	1710.5977	(Neu5Ac)Gal(GlcNAc)Man$_4$(GlcNAc)$_2$	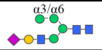
1182.1581(3+)	3544.4587	3544.4590	0	1671.8085	K.^{292}TKPREEQY^{300}NSTYR^{304}.V	1872.6505	(Neu5Ac)Gal(GlcNAc)Man$_5$(GlcNAc)$_2$	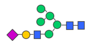
1141.8140(3+)	3423.4264	3423.4327	-2	1671.8085	K.^{292}TKPREEQY^{300}NSTYR^{304}.V	1751.6242	(Neu5Ac)Gal(GlcNAc)$_2$Man$_3$(GlcNAc)$_2$	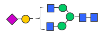
1195.8342(3+)	3585.487	3585.4855	0	1671.8085	K.^{292}TKPREEQY^{300}NSTYR^{304}.V	1913.6770	(Neu5Ac)Gal$_2$(GlcNAc)$_2$Man$_3$(GlcNAc)$_2$	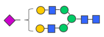
842.3586(4+)	3366.4109	3366.4112	0	1671.8085	K.^{292}TKPREEQY^{300}NSTYR^{304}.V	1694.6027	(Neu5Ac)Gal(GlcNAc)Man$_3$(GlcNAc)$_2$Fuc	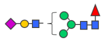
923.3842(4+)	3690.5133	3690.5169	-1	1671.8085	K.^{292}TKPREEQY^{300}NSTYR^{304}.V	2018.7084	(Neu5Ac)Gal(GlcNAc)Man$_5$(GlcNAc)$_2$Fuc	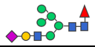
1122.8086(3+)	3366.4102	3366.4112	0	1671.8085	K.^{292}TKPREEQY^{300}NSTYR^{304}.V	1694.6027	(Neu5Ac)Gal(GlcNAc)Man$_3$(GlcNAc)$_2$Fuc	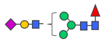

893.1288(4+)	3569.4917	3569.4906	0	1671.8085	K.^{292}TKPREEQY^{300}NSTYR^{304}.V	1897.6821	(Neu5Ac)Gal(GlcNAc)$_2$Man$_3$(GlcNAc)$_2$Fuc	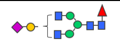
1190.5016(3+)	3569.4892	3569.4906	0	1671.8085	K.^{292}TKPREEQY^{300}NSTYR^{304}.V	1897.6821	(Neu5Ac)Gal(GlcNAc)$_2$Man$_3$(GlcNAc)$_2$Fuc	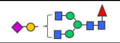
933.6427(4+)	3731.5473	2059.7349	1	1671.8085	K.^{292}TKPREEQY^{300}NSTYR^{304}.V	2059.7349	(Neu5Ac)Gal$_2$(GlcNAc)$_2$Man$_3$(GlcNAc)$_2$Fuc	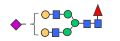
1244.5220(3+)	3731.5504	3731.5434	2	1671.8085	K.^{292}TKPREEQY^{300}NSTYR^{304}.V	2059.7349	(Neu5Ac)Gal$_2$(GlcNAc)$_2$Man$_3$(GlcNAc)$_2$Fuc	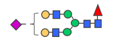
1041.4185(2+)	2081.8292	2081.8292	0	1189.5120	R.^{296}EEQY^{300}NSTYR^{304}.V	892.3172	Man$_3$(GlcNAc)$_2$	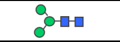
1122.4453(2+)	2243.8828	2243.8820	0	1189.5120	R.^{296}EEQY^{300}NSTYR^{304}.V	1054.3700	Man$_4$(GlcNAc)$_2$	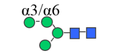
802.6495(3+)	2405.9329	2405.9349	-1	1189.5120	R.^{296}EEQY^{300}NSTYR^{304}.V	1216.4229	Man$_5$(GlcNAc)$_2$	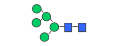
1203.4702(2+)	2405.9326	2405.9349	-1	1189.5120	R.^{296}EEQY^{300}NSTYR^{304}.V	1216.4229	Man$_5$(GlcNAc)$_2$	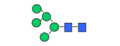
1284.4983(2+)	2567.9888	2567.9877	0	1189.5120	R.^{296}EEQY^{300}NSTYR^{304}.V	1378.4757	Man$_6$(GlcNAc)$_2$	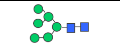
856.6672(3+)	2567.9860	2567.9877	-1	1189.5120	R.^{296}EEQY^{300}NSTYR^{304}.V	1378.4757	Man$_6$(GlcNAc)$_2$	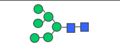
1142.9575(2+)	2284.9072	2284.9086	-1	1189.5120	R.^{296}EEQY^{300}NSTYR^{304}.V	1095.3966	(GlcNAc)Man$_3$(GlcNAc)$_2$	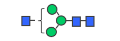
1244.4976(2+)	2487.9874	2487.9880	0	1189.5120	R.^{296}EEQY^{300}NSTYR^{304}.V	1298.4760	(GlcNAc)$_2$Man$_3$(GlcNAc)$_2$	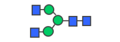
1325.5227(2+)	2650.0376	2650.0408	-1	1189.5120	R.^{296}EEQY^{300}NSTYR^{304}.V	1460.5288	Gal(GlcNAc)$_2$Man$_3$(GlcNAc)$_2$	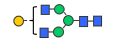
1386.0367(2+)	2771.0656	2771.0671	-1	1189.5120	R.^{296}EEQY^{300}NSTYR^{304}.V	1581.5551	Gal(GlcNAc)Man$_5$(GlcNAc)$_2$	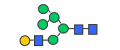
1134.9601(2+)	2268.9124	2268.9135	-1	1189.5120	R.^{296}EEQY^{300}NSTYR^{304}.V	1079.4015	(GlcNAc)Man$_2$(GlcNAc)$_2$Fuc	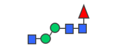
1215.9860(2+)	2430.9642	2430.9665	-1	1189.5120	R.^{296}EEQY^{300}NSTYR^{304}.V	1241.4545	(GlcNAc)Man$_3$(GlcNAc)$_2$Fuc	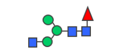
878.6880(3+)	2634.0484	2634.0459	1	1189.5120	R.^{296}EEQY^{300}NSTYR^{304}.V	1444.5339	(GlcNAc)$_2$Man$_3$(GlcNAc)$_2$Fuc	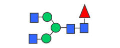
1317.5275(2+)	2634.0472	2634.0459	1	1189.5120	R.^{296}EEQY^{300}NSTYR^{304}.V	1444.5339	(GlcNAc)$_2$Man$_3$(GlcNAc)$_2$Fuc	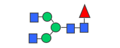
865.0118(3+)	2593.0198	2593.0193	0	1189.5120	R.^{296}EEQY^{300}NSTYR^{304}.V	1403.5073	Gal(GlcNAc)Man$_3$(GlcNAc)$_2$Fuc	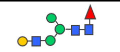
1297.0125(2+)	2593.0172	2593.0193	-1	1189.5120	R.^{296}EEQY^{300}NSTYR^{304}.V	1403.5073	Gal(GlcNAc)Man$_3$(GlcNAc)$_2$Fuc	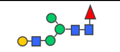
1398.5515(2+)	2796.0952	2796.0987	-1	1189.5120	R.^{296}EEQY^{300}NSTYR^{304}.V	1606.5867	Gal(GlcNAc)$_2$Man$_3$(GlcNAc)$_2$Fuc	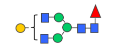
932.7063(3+)	2796.1033	2796.0987	2	1189.5120	R.^{296}EEQY^{300}NSTYR^{304}.V	1606.5867	Gal(GlcNAc)$_2$Man$_3$(GlcNAc)$_2$Fuc	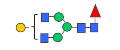
1398.5524(2+)	2796.097	2796.0987	-1	1189.5120	R.^{296}EEQY^{300}NSTYR^{304}.V	1606.5867	Gal(GlcNAc)$_2$Man$_3$(GlcNAc)$_2$Fuc	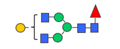
1398.5525(2+)	2796.0972	2796.0987	-1	1189.5120	R.^{296}EEQY^{300}NSTYR^{304}.V	1606.5867	Gal(GlcNAc)$_2$Man$_3$(GlcNAc)$_2$Fuc	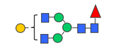
986.7225(3+)	2958.1519	2958.1515	0	1189.5120	R.^{296}EEQY^{300}NSTYR^{304}.V	1768.6395	Gal$_2$(GlcNAc)$_2$Man$_3$(GlcNAc)$_2$Fuc	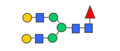
1479.5792(2+)	2958.1506	2958.1515	0	1189.5120	R.^{296}EEQY^{300}NSTYR^{304}.V	1768.6395	Gal$_2$(GlcNAc)$_2$Man$_3$(GlcNAc)$_2$Fuc	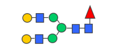
1040.7395(3+)	3120.2029	3120.2043	0	1189.5120	R.^{296}EEQY^{300}NSTYR^{304}.V	1930.6923	Gal$_3$(GlcNAc)$_2$Man$_3$(GlcNAc)$_2$Fuc	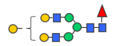
1369.5314(2+)	2738.0550	2738.0568	-1	1189.5120	R.^{296}EEQY^{300}NSTYR^{304}.V	1548.5448	(Neu5Ac)Gal(GlcNAc)Man$_3$(GlcNAc)$_2$	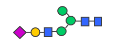
967.3741(3+)	2900.1067	2900.1097	-1	1189.5120	R.^{296}EEQY^{300}NSTYR^{304}.V	1710.5977	(Neu5Ac)Gal(GlcNAc)Man$_4$(GlcNAc)$_2$	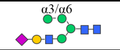
1450.5582(2+)	2900.1086	2900.1096	0	1189.5120	R.^{296}EEQY^{300}NSTYR^{304}.V	1710.5976	(Neu5Ac)Gal(GlcNAc)Man$_4$(GlcNAc)$_2$	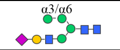
1471.0710(2+)	2941.1342	2941.1362	-1	1189.5120	R.^{296}EEQY^{300}NSTYR^{304}.V	1751.6242	(Neu5Ac)Gal(GlcNAc)$_2$Man$_3$(GlcNAc)$_2$	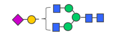
962.0427(3+)	2884.1125	2884.1147	-1	1189.5120	R.^{296}EEQY^{300}NSTYR^{304}.V	1694.6027	(Neu5Ac)Gal(GlcNAc)Man$_3$(GlcNAc)$_2$Fuc	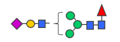
1442.5625(2+)	2884.1172	2884.1147	1	1189.5120	R.^{296}EEQY^{300}NSTYR^{304}.V	1694.6027	(Neu5Ac)Gal(GlcNAc)Man$_3$(GlcNAc)$_2$Fuc	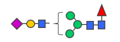
1523.5889(2+)	3046.1700	3046.1676	1	1189.5120	R.^{296}EEQY^{300}NSTYR^{304}.V	1856.6556	(Neu5Ac)Gal(GlcNAc)Man$_4$(GlcNAc)$_2$Fuc	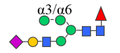

1070.0773(3+)	3208.2163	3208.2204	-1	1189.5120	R.^{296}EEQY^{300}NSTYR^{304}.V	2018.7084	(Neu5Ac)Gal(GlcNAc)Man$_5$(GlcNAc)$_2$Fuc	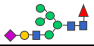
1604.6123(2+)	3208.2168	3208.2204	-1	1189.5120	R.^{296}EEQY^{300}NSTYR^{304}.V	2018.7084	(Neu5Ac)Gal(GlcNAc)Man$_5$(GlcNAc)$_2$Fuc	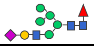
1029.7361(3+)	3087.1927	3087.1941	0	1189.5120	R.^{296}EEQY^{300}NSTYR^{304}.V	1897.6821	(Neu5Ac)Gal(GlcNAc)$_2$Man$_3$(GlcNAc)$_2$Fuc	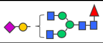
1544.1025(2+)	3087.1972	3087.1941	1	1189.5120	R.^{296}EEQY^{300}NSTYR^{304}.V	1897.6821	(Neu5Ac)Gal(GlcNAc)$_2$Man$_3$(GlcNAc)$_2$Fuc	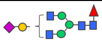
1083.7532(3+)	3249.2440	3249.2469	-1	1189.5120	R.^{296}EEQY^{300}NSTYR^{304}.V	2059.7349	(Neu5Ac)Gal$_2$(GlcNAc)$_2$Man$_3$(GlcNAc)$_2$Fuc	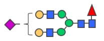
1625.1284(2+)	3249.2490	3249.2469	1	1189.5120	R.^{296}EEQY^{300}NSTYR^{304}.V	2059.7349	(Neu5Ac)Gal$_2$(GlcNAc)$_2$Man$_3$(GlcNAc)$_2$Fuc	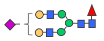
1377.5281(2+)	2754.0484	2754.0517	-1	1189.5120	R.^{296}EEQY^{300}NSTYR^{304}.V	1564.5397	(Neu5Gc)Gal(GlcNAc)Man$_3$(GlcNAc)$_2$	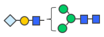
1035.0688(3+)	3103.1908	3103.1890	1	1189.5120	R.^{296}EEQY^{300}NSTYR^{304}.V	1913.6770	(Neu5Gc)Gal(GlcNAc)$_2$Man$_3$(GlcNAc)$_2$Fuc	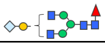
1035.0682(3+)	3103.1890	3103.1890	0	1189.5120	R.^{296}EEQY^{300}NSTYR^{304}.V	1913.6770	(Neu5Ac)Gal$_2$(GlcNAc)$_2$Man$_3$(GlcNAc)$_2$	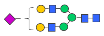
1552.0952(2+)	3103.1826	3103.1890	-2	1189.5120	R.^{296}EEQY^{300}NSTYR^{304}.V	1913.6770	(Neu5Gc)Gal(GlcNAc)$_2$Man$_3$(GlcNAc)$_2$Fuc	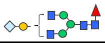
1552.1005(2+)	3103.1932	3103.1890	1	1189.5120	R.^{296}EEQY^{300}NSTYR^{304}.V	1913.6770	(Neu5Ac)Gal$_2$(GlcNAc)$_2$Man$_3$(GlcNAc)$_2$	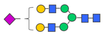
1089.0851(3+)	3265.2397	3265.2418	-1	1189.5120	R.^{296}EEQY^{300}NSTYR^{304}.V	2075.7298	(Neu5Gc)Gal$_2$(GlcNAc)$_2$Man$_3$(GlcNAc)$_2$Fuc	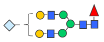
1089.0857(3+)	3265.2415	3265.2418	0	1189.5120	R.^{296}EEQY^{300}NSTYR^{304}.V	2075.7298	(Neu5Ac)Gal$_3$(GlcNAc)$_2$Man$_3$(GlcNAc)$_2$	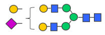
1633.1246(2+)	3265.2414	3265.2418	0	1189.5120	R.^{296}EEQY^{300}NSTYR^{304}.V	2075.7298	(Neu5Gc)Gal$_3$(GlcNAc)$_2$Man$_3$(GlcNAc)$_2$	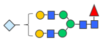
1633.1248(2+)	3265.0418	3265.2418	0	1189.5120	R.^{296}EEQY^{300}NSTYR^{304}.V	2075.7298	(Neu5Ac)Gal$_3$(GlcNAc)$_2$Man$_3$(GlcNAc)$_2$	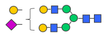
887.0343(3+)	2659.0873	2659.0888	-1	1442.6659	K.^{294}PREEQY^{300}NSTYR^{304}.V	1216.4229	Man$_5$(GlcNAc)$_2$	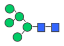
914.3859(3+)	2741.1421	2741.1419	0	1442.6659	K.^{294}PREEQY^{300}NSTYR^{304}.V	1298.4760	(GlcNAc)$_2$Man$_3$(GlcNAc)$_2$	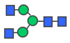
963.0718(3+)	2887.1998	2887.1998	0	1442.6659	K.^{294}PREEQY^{300}NSTYR^{304}.V	1444.5339	(GlcNAc)$_2$Man$_3$(GlcNAc)$_2$Fuc	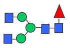
1017.0895(3+)	3049.2529	3049.2526	0	1442.6659	K.^{294}PREEQY^{300}NSTYR^{304}.V	1606.5867	Gal(GlcNAc)$_2$Man$_3$(GlcNAc)$_2$Fuc	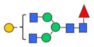
1071.1064(3+)	3211.3036	3211.3054	-1	1442.6659	K.^{294}PREEQY^{300}NSTYR^{304}.V	1768.6395	Gal$_2$(GlcNAc)$_2$Man$_3$(GlcNAc)$_2$Fuc	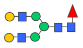
1168.1385(3+)	3502.3999	3502.4008	0	1442.6659	K.^{294}PREEQY^{300}NSTYR^{304}.V	2059.7349	(Neu5Ac)Gal$_2$(GlcNAc)$_2$Man$_3$(GlcNAc)$_2$Fuc	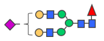

* The N-glycopeptides are determined by reversed-phase (RP) LC MS/MS of a tryptic Trastuzumab digest, and identified by database search using Byonic software.

§ Missed cleavages of Fc peptides at residues TKPREEQYNSTYR were also observed due to a low hydrolysis rate caused by the suppression of trypsin activity resulting from the co-existence of acidic residues (*i.e.* glutamic acid) and proline near the cleavage site (Rodriguez, J.; Gupta, N.; Smith, R. D.; Pevzner, P. A. *J Proteome Res.* **2008**, 7, 300-305).

\# Possible structures of N-glycans are predicted based on the initial database search against the in-house mAb glycan library by the accurate mass, glycan composition and the structures derived from the glycan biosynthesis pathway. The isomeric glycan structures are not distinguishable at the glycopeptide level.

Table S2. Identification of the tryptic glycopeptides of rituximab derived from CHO cells by reversed-phase LC MS/MS

Glycopeptide *m/z* (charge) *	Meas. MH+	Calc. MH+	ppm	Peptide MH+	Peptide sequence §	Glycan mass	Glycan composition	Putative glycan structure #
850.6100(4+)	3399.4165	3399.4214	-2	1671.8085	K.^{293}TKPREEQY^{301}NSTYR^{305}.V	1727.6129	Gal(GlcNAc)Man$_5$(GlcNAc)$_2$Fuc	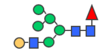
917.7407(3+)	2751.2065	2751.2100	-1	1671.8085	K.^{293}TKPREEQY^{301}NSTYR^{305}.V	1079.4015	(GlcNAc)Man$_2$(GlcNAc)$_2$Fuc	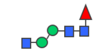
971.7581(3+)	2913.2587	2913.2630	-2	1671.8085	K.^{293}TKPREEQY^{301}NSTYR^{305}.V	1241.4545	(GlcNAc)Man$_3$(GlcNAc)$_2$Fuc	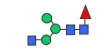
779.8406(4+)	3116.3389	3116.3424	-1	1671.8085	K.^{293}TKPREEQY^{301}NSTYR^{305}.V	1444.5339	(GlcNAc)$_2$Man$_3$(GlcNAc)$_2$Fuc	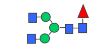
1039.4514(3+)	3116.3386	3116.3424	-1	1671.8085	K.^{293}TKPREEQY^{301}NSTYR^{305}.V	1444.5339	(GlcNAc)$_2$Man$_3$(GlcNAc)$_2$Fuc	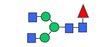
1025.7773(3+)	3075.3163	3075.3158	0	1671.8085	K.^{293}TKPREEQY^{301}NSTYR^{305}.V	1403.5073	Gal(GlcNAc)Man$_3$(GlcNAc)$_2$Fuc	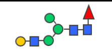
820.3541(4+)	3278.3929	3278.3952	-1	1671.8085	K.^{293}TKPREEQY^{301}NSTYR^{305}.V	1606.5867	Gal(GlcNAc)$_2$Man$_3$(GlcNAc)$_2$Fuc	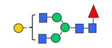
1093.4702(3+)	3278.3950	3278.3952	0	1671.8085	K.^{293}TKPREEQY^{301}NSTYR^{305}.V	1606.5867	Gal(GlcNAc)$_2$Man$_3$(GlcNAc)$_2$Fuc	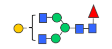
860.8671(4+)	3440.4449	3440.4480	-1	1671.8085	K.^{293}TKPREEQY^{301}NSTYR^{305}.V	1768.6395	Gal$_2$(GlcNAc)$_2$Man$_3$(GlcNAc)$_2$Fuc	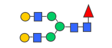
1147.4882(3+)	3440.4490	3440.4480	0	1671.8085	K.^{293}TKPREEQY^{301}NSTYR^{305}.V	1768.6395	Gal$_2$(GlcNAc)$_2$Man$_3$(GlcNAc)$_2$Fuc	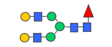
842.3591(4+)	3366.4129	3366.4112	1	1671.8085	K.^{293}TKPREEQY^{301}NSTYR^{305}.V	1694.6027	(Neu5Ac)Gal(GlcNAc)Man$_3$(GlcNAc)$_2$Fuc	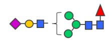
1122.8092(3+)	3366.412	3366.4112	0	1671.8085	K.^{293}TKPREEQY^{301}NSTYR^{305}.V	1694.6027	(Neu5Ac)Gal(GlcNAc)Man$_3$(GlcNAc)$_2$Fuc	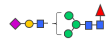
923.3859(4+)	3690.5201	3690.5169	1	1671.8085	K.^{293}TKPREEQY^{301}NSTYR^{305}.V	2018.7084	(Neu5Ac)Gal(GlcNAc)Man$_5$(GlcNAc)$_2$Fuc	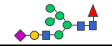
893.1291(4+)	3569.4929	3569.4906	1	1671.8085	K.^{293}TKPREEQY^{301}NSTYR^{305}.V	1897.6821	(Neu5Ac)Gal(GlcNAc)$_2$Man$_3$(GlcNAc)$_2$Fuc	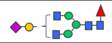
1190.5029(3+)	3569.4931	3569.4906	1	1671.8085	K.^{293}TKPREEQY^{301}NSTYR^{305}.V	1897.6821	(Neu5Ac)Gal(GlcNAc)$_2$Man$_3$(GlcNAc)$_2$Fuc	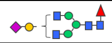
933.6420(4+)	3731.5445	3731.5434	0	1671.8085	K.^{293}TKPREEQY^{301}NSTYR^{305}.V	2059.7349	(Neu5Ac)Gal$_2$(GlcNAc)$_2$Man$_3$(GlcNAc)$_2$Fuc	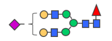
1142.9570(2+)	2284.9062	2284.9086	-1	1189.5120	R.^{297}EEQY^{301}NSTYR^{305}.V	1095.3966	(GlcNAc)Man$_3$(GlcNAc)$_2$	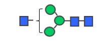
1244.4967(2+)	2487.9856	2487.9880	-1	1189.5120	R.^{297}EEQY^{301}NSTYR^{305}.V	1298.4760	(GlcNAc)$_2$Man$_3$(GlcNAc)$_2$	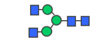
1134.9592(2+)	2268.9106	2268.9135	-1	1189.5120	R.^{297}EEQY^{301}NSTYR^{305}.V	1079.4015	(GlcNAc)Man$_2$(GlcNAc)$_2$Fuc	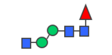
1215.9867(2+)	2430.9656	2430.9665	0	1189.5120	R.^{297}EEQY^{301}NSTYR^{305}.V	1241.4545	(GlcNAc)Man$_3$(GlcNAc)$_2$Fuc	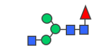
878.6869(3+)	2634.0451	2634.0459	0	1189.5120	R.^{297}EEQY^{301}NSTYR^{305}.V	1444.5339	(GlcNAc)$_2$Man$_3$(GlcNAc)$_2$Fuc	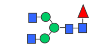
1317.5262(2+)	2634.0446	2634.0459	-1	1189.5120	R.^{297}EEQY^{301}NSTYR^{305}.V	1444.5339	(GlcNAc)$_2$Man$_3$(GlcNAc)$_2$Fuc	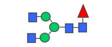
1297.0134(2+)	2593.0190	2593.0193	0	1189.5120	R.^{297}EEQY^{301}NSTYR^{305}.V	1403.5073	Gal(GlcNAc)Man$_3$(GlcNAc)$_2$Fuc	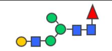
932.7056(3+)	2796.1012	2796.0987	1	1189.5120	R.^{297}EEQY^{301}NSTYR^{305}.V	1606.5867	Gal(GlcNAc)$_2$Man$_3$(GlcNAc)$_2$Fuc	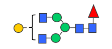
1398.5526(2+)	2796.0974	2796.0987	0	1189.5120	R.^{297}EEQY^{301}NSTYR^{305}.V	1606.5867	Gal(GlcNAc)$_2$Man$_3$(GlcNAc)$_2$Fuc	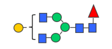
986.7219(3+)	2958.1501	2958.1515	-1	1189.5120	R.^{297}EEQY^{301}NSTYR^{305}.V	1768.6395	Gal$_2$(GlcNAc)$_2$Man$_3$(GlcNAc)$_2$Fuc	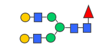
1479.5785(2+)	2958.1492	2958.1515	-1	1189.5120	R.^{297}EEQY^{301}NSTYR^{305}.V	1768.6395	Gal$_2$(GlcNAc)$_2$Man$_3$(GlcNAc)$_2$Fuc	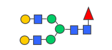
962.0427(3+)	2884.1125	2884.1147	-1	1189.5120	R.^{297}EEQY^{301}NSTYR^{305}.V	1694.6027	(Neu5Ac)Gal(GlcNAc)Man$_3$(GlcNAc)$_2$Fuc	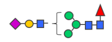
1442.5610(2+)	2884.1142	2884.1147	0	1189.5120	R.^{297}EEQY^{301}NSTYR^{305}.V	1694.6027	(Neu5Ac)Gal(GlcNAc)Man$_3$(GlcNAc)$_2$Fuc	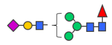
1016.0607(3+)	3046.1665	3046.1676	0	1189.5120	R.^{297}EEQY^{301}NSTYR^{305}.V	1856.6556	(Neu5Ac)Gal(GlcNAc)Man$_4$(GlcNAc)$_2$Fuc	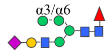

1523.5878(2+)	3046.1678	3046.1676	0	1189.5120	R.^{297}EEQY^{301}NSTYR^{305}.V	1856.6556	(Neu5Ac)Gal(GlcNAc)Man$_4$(GlcNAc)$_2$Fuc	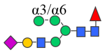
1070.0780(3+)	3208.2184	3208.2204	1	1189.5120	R.^{297}EEQY^{301}NSTYR^{305}.V	2018.7084	(Neu5Ac)Gal(GlcNAc)Man$_5$(GlcNAc)$_2$Fuc	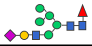
1604.6154(2+)	3208.223	3208.2204	1	1189.5120	R.^{297}EEQY^{301}NSTYR^{305}.V	2018.7084	(Neu5Ac)Gal(GlcNAc)Man$_5$(GlcNAc)$_2$Fuc	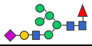
1029.7368(3+)	3087.1948	3087.1941	0	1189.5120	R.^{297}EEQY^{301}NSTYR^{305}.V	1897.6821	(Neu5Ac)Gal(GlcNAc)$_2$Man$_3$(GlcNAc)$_2$Fuc	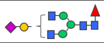
1544.1026(2+)	3087.1974	3087.1941	1	1189.5120	R.^{297}EEQY^{301}NSTYR^{305}.V	1897.6821	(Neu5Ac)Gal(GlcNAc)$_2$Man$_3$(GlcNAc)$_2$Fuc	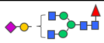
1625.1282(2+)	3249.2486	3249.2469	1	1189.5120	R.^{297}EEQY^{301}NSTYR^{305}.V	2059.7349	(Neu5Ac)Gal$_2$(GlcNAc)$_2$Man$_3$(GlcNAc)$_2$Fuc	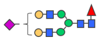
1083.7549(3+)	3249.2491	3249.2469	1	1189.5120	R.^{297}EEQY^{301}NSTYR^{305}.V	2059.7349	(Neu5Ac)Gal$_2$(GlcNAc)$_2$Man$_3$(GlcNAc)$_2$Fuc	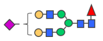
963.0717(3+)	2887.1995	2887.1998	0	1442.6659	K.^{295}PREEQY^{301}NSTYR^{305}.V	1444.5339	(GlcNAc)$_2$Man$_3$(GlcNAc)$_2$Fuc	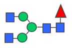
1017.0897(3+)	3049.2535	3049.2526	0	1442.6659	K.^{295}PREEQY^{301}NSTYR^{305}.V	1606.5867	Gal(GlcNAc)$_2$Man$_3$(GlcNAc)$_2$Fuc	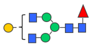
1071.1068(3+)	3211.3048	3211.3054	0	1442.6659	K.^{295}PREEQY^{301}NSTYR^{305}.V	1768.6395	Gal$_2$(GlcNAc)$_2$Man$_3$(GlcNAc)$_2$Fuc	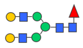

* The N-glycopeptides are determined by reversed-phase (RP) LC MS/MS of a tryptic Rituximab digest, and identified by database search using Byonic software.

§ Missed cleavages of Fc peptides at residues TKPREEQYNSTYR were also observed due to a low hydrolysis rate caused by the suppression of trypsin activity resulting from the co-existence of acidic residues (*i.e.* glutamic acid) and proline near the cleavage site (Rodriguez, J.; Gupta, N.; Smith, R. D.; Pevzner, P. A. *J Proteome Res.* **2008**, 7, 300-305).

# Possible structures of N-glycans are predicted based on the initial database search against the in-house mAb glycan library by the accurate mass, glycan composition and the structures derived from the glycan biosynthesis pathway. The isomeric glycan structures are not distinguishable at the glycopeptide level.

Table S3. Identification of the tryptic glycopeptides of infliximab derived from murine myeloma cells by reversed-phase LC MS/MS

Glycopeptide *m/z* (charge)*	Meas. MH+	Calc. MH+	ppm	Peptide MH+	Peptide sequence §	Glycan mass	Glycan composition	Glycan structure #
855.3802(3+)	2564.125	2564.1257	0	1671.8085	K.^{294}TKPREEQY^{302}NSTYR^{306}.V	892.3172	Man$_3$(GlcNAc)$_2$	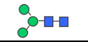
682.2994(4+)	2726.1741	2726.1785	-2	1671.8085	K.^{294}TKPREEQY^{302}NSTYR^{306}.V	1054.3700	Man$_4$(GlcNAc)$_2$	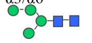
909.3969(3+)	2726.1751	2726.1785	-1	1671.8085	K.^{294}TKPREEQY^{302}NSTYR^{306}.V	1054.3700	Man$_4$(GlcNAc)$_2$	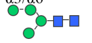
722.8134(4+)	2888.2301	2888.2314	0	1671.8085	K.^{294}TKPREEQY^{302}NSTYR^{306}.V	1216.4229	Man$_5$(GlcNAc)$_2$	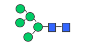
963.4146(3+)	2888.2282	2888.2314	-1	1671.8085	K.^{294}TKPREEQY^{302}NSTYR^{306}.V	1216.4229	Man$_5$(GlcNAc)$_2$	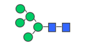
1017.4338(3+)	3050.2858	3050.2842	1	1671.8085	K.^{294}TKPREEQY^{302}NSTYR^{306}.V	1378.4757	Man$_6$(GlcNAc)$_2$	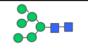
803.8393(4+)	3212.3337	3212.3370	-1	1671.8085	K.^{294}TKPREEQY^{302}NSTYR^{306}.V	1540.5285	Man$_7$(GlcNAc)$_2$	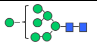
844.3533(4+)	3374.3897	3374.3898	0	1671.8085	K.^{294}TKPREEQY^{302}NSTYR^{306}.V	1702.5813	Man$_8$(GlcNAc)$_2$	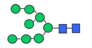
1012.1025(3+)	3034.2919	3034.2893	1	1671.8085	K.^{294}TKPREEQY^{302}NSTYR^{306}.V	1362.4808	Man$_5$(GlcNAc)$_2$Fuc	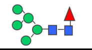
773.5829(4+)	3091.3081	3091.3107	-1	1671.8085	K.^{294}TKPREEQY^{302}NSTYR^{306}.V	1419.5022	(GlcNAc)Man$_5$(GlcNAc)$_2$	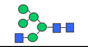
1031.1089(3+)	1031.1089	3091.3107	0	1671.8085	K.^{294}TKPREEQY^{302}NSTYR^{306}.V	1419.5022	(GlcNAc)Man$_5$(GlcNAc)$_2$	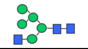
814.0966(4+)	3253.3629	3253.3636	0	1671.8085	K.^{294}TKPREEQY^{302}NSTYR^{306}.V	1581.5551	Gal(GlcNAc)Man$_5$(GlcNAc)$_2$	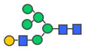
1085.1255(3+)	3253.3609	3253.3636	-1	1671.8085	K.^{294}TKPREEQY^{302}NSTYR^{306}.V	1581.5551	Gal(GlcNAc)Man$_5$(GlcNAc)$_2$	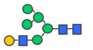
854.6095(4+)	3415.4145	3415.4164	-1	1671.8085	K.^{294}TKPREEQY^{302}NSTYR^{306}.V	1743.6079	Gal$_2$(GlcNAc)Man$_5$(GlcNAc)$_2$	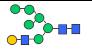
810.0977(4+)	3237.3673	3237.3686	0	1671.8085	K.^{294}TKPREEQY^{302}NSTYR^{306}.V	1565.5601	(GlcNAc)Man$_5$(GlcNAc)$_2$Fuc	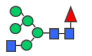
1079.7949(3+)	3237.3691	3237.3686	0	1671.8085	K.^{294}TKPREEQY^{302}NSTYR^{306}.V	1565.5601	(GlcNAc)Man$_5$(GlcNAc)$_2$Fuc	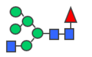
850.6105(4+)	3399.4185	3399.4214	-1	1671.8085	K.^{294}TKPREEQY^{302}NSTYR^{306}.V	1727.6129	Gal(GlcNAc)Man$_5$(GlcNAc)$_2$Fuc	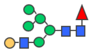
1133.8114(3+)	3399.4186	3399.4214	-1	1671.8085	K.^{294}TKPREEQY^{302}NSTYR^{306}.V	1727.6129	Gal(GlcNAc)Man$_5$(GlcNAc)$_2$Fuc	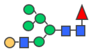
891.1236(4+)	3561.4709	3561.4743	-1	1671.8085	K.^{294}TKPREEQY^{302}NSTYR^{306}.V	1889.6658	Gal(GlcNAc)Man$_6$(GlcNAc)$_2$Fuc	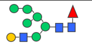
923.0726(3+)	2767.2022	2767.2051	-1	1671.8085	K.^{294}TKPREEQY^{302}NSTYR^{306}.V	1095.3966	(GlcNAc)Man$_3$(GlcNAc)$_2$	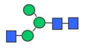
990.7659(3+)	2970.2821	2970.2845	-1	1671.8085	K.^{294}TKPREEQY^{302}NSTYR^{306}.V	1298.4760	(GlcNAc)$_2$Man$_3$(GlcNAc)$_2$	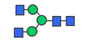
743.3264(4+)	2970.2821	2970.2845	-1	1671.8085	K.^{294}TKPREEQY^{302}NSTYR^{306}.V	1298.4760	(GlcNAc)$_2$Man$_3$(GlcNAc)$_2$	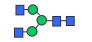
977.0907(3+)	2929.2565	2929.2579	-1	1671.8085	K.^{294}TKPREEQY^{302}NSTYR^{306}.V	1257.4494	(GlcNAc)Man$_4$(GlcNAc)$_2$	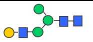
917.7409(3+)	2751.2071	2751.2102	-1	1671.8085	K.^{294}TKPREEQY^{302}NSTYR^{306}.V	1079.4017	(GlcNAc)Man$_2$(GlcNAc)$_2$Fuc	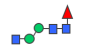
971.7590(3+)	2913.2614	2913.2630	-1	1671.8085	K.^{294}TKPREEQY^{302}NSTYR^{306}.V	1241.4545	(GlcNAc)Man$_3$(GlcNAc)$_2$Fuc	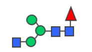
779.8409(4+)	3116.3401	3116.3424	-1	1671.8085	K.^{294}TKPREEQY^{302}NSTYR^{306}.V	1444.5339	(GlcNAc)$_2$Man$_3$(GlcNAc)$_2$Fuc	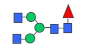
1039.4520(3+)	3116.3404	3116.3424	-1	1671.8085	K.^{294}TKPREEQY^{302}NSTYR^{306}.V	1444.5339	(GlcNAc)$_2$Man$_3$(GlcNAc)$_2$Fuc	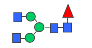
769.5849(4+)	3075.3161	3075.3158	0	1671.8085	K.^{294}TKPREEQY^{302}NSTYR^{306}.V	1403.5073	Gal(GlcNAc)Man$_3$(GlcNAc)$_2$Fuc	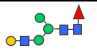
1025.7767(3+)	3075.3145	3075.3158	0	1671.8085	K.^{294}TKPREEQY^{302}NSTYR^{306}.V	1403.5073	Gal(GlcNAc)Man$_3$(GlcNAc)$_2$Fuc	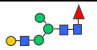
820.3546(4+)	3278.3949	3278.3952	0	1671.8085	K.^{294}TKPREEQY^{302}NSTYR^{306}.V	1606.5867	Gal(GlcNAc)$_2$Man$_3$(GlcNAc)$_2$Fuc	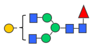
1093.4695(3+)	3278.3929	3278.3952	-1	1671.8085	K.^{294}TKPREEQY^{302}NSTYR^{306}.V	1606.5867	Gal(GlcNAc)$_2$Man$_3$(GlcNAc)$_2$Fuc	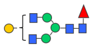

860.8674(4+)	3440.4461	3440.4480	-1	1671.8085	K.^{294}TKPREEQY^{302}NSTYR^{306}.V	1768.6395	Gal$_2$(GlcNAc)$_2$Man$_3$(GlcNAc)$_2$Fuc	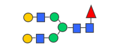
1147.4872(3+)	3440.4460	3440.4480	-1	1671.8085	K.^{294}TKPREEQY^{302}NSTYR^{306}.V	1768.6395	Gal$_2$(GlcNAc)$_2$Man$_3$(GlcNAc)$_2$Fuc	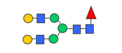
901.3802(4+)	3602.4973	3602.5008	-1	1671.8085	K.^{294}TKPREEQY^{302}NSTYR^{306}.V	1930.6923	Gal$_3$(GlcNAc)$_2$Man$_3$(GlcNAc)$_2$Fuc	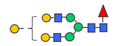
933.6404 (4+)	3731.5381	3731.5434	-2	1671.8085	K.^{294}TKPREEQY^{302}NSTYR^{306}.V	2059.7349	(Neu5Ac)Gal$_2$(GlcNAc)$_2$Man$_3$(GlcNAc)$_2$Fuc	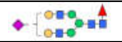
809.8429(4+)	3236.3481	3236.3482	0	1671.8085	K.^{294}TKPREEQY^{302}NSTYR^{306}.V	1564.5397	(Neu5Gc)Gal(GlcNAc)Man$_3$(GlcNAc)$_2$	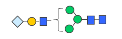
1079.4550(3+)	3236.3494	3236.3482	0	1671.8085	K.^{294}TKPREEQY^{302}NSTYR^{306}.V	1564.5397	(Neu5Gc)Gal(GlcNAc)Man$_3$(GlcNAc)$_2$	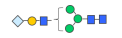
850.3553(4+)	3398.3977	3398.4011	-1	1671.8085	K.^{294}TKPREEQY^{302}NSTYR^{306}.V	1726.5926	(Neu5Gc)Gal(GlcNAc)Man$_4$(GlcNAc)$_2$	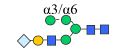
1133.4712(3+)	3398.398	3398.4011	-1	1671.8085	K.^{294}TKPREEQY^{302}NSTYR^{306}.V	1726.5926	(Neu5Gc)Gal(GlcNAc)Man$_4$(GlcNAc)$_2$	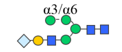
1187.4899(3+)	3560.4541	3560.4539	0	1671.8085	K.^{294}TKPREEQY^{302}NSTYR^{306}.V	1888.6454	(Neu5Gc)Gal(GlcNAc)Man$_5$(GlcNAc)$_2$	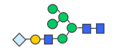
890.8690(4+)	3560.4525	1888.6454	0	1671.8085	K.^{294}TKPREEQY^{302}NSTYR^{306}.V	1888.6454	(Neu5Gc)Gal(GlcNAc)Man$_5$(GlcNAc)$_2$	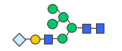
1128.1411(3+)	3382.4077	3382.4062	0	1671.8085	K.^{294}TKPREEQY^{302}NSTYR^{306}.V	1710.5977	(Neu5Gc)Gal(GlcNAc)Man$_3$(GlcNAc)$_2$Fuc	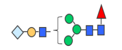
886.8705(4+)	3544.4585	3544.4590	0	1671.8085	K.^{294}TKPREEQY^{302}NSTYR^{306}.V	1872.6505	(Neu5Gc)Gal(GlcNAc)Man$_4$(GlcNAc)$_2$Fuc	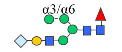
1182.1572(3+)	3544.456	3544.459	-1	1671.8085	K.^{294}TKPREEQY^{302}NSTYR^{306}.V	1872.6505	(Neu5Gc)Gal(GlcNAc)Man$_4$(GlcNAc)$_2$Fuc	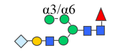
846.3569(4+)	3382.4041	3382.4062	-1	1671.8085	K.^{294}TKPREEQY^{302}NSTYR^{306}.V	1710.5977	(Neu5Gc)Gal(GlcNAc)Man$_3$(GlcNAc)$_2$Fuc	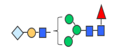
1128.1400(3+)	3382.4044	3382.4062	-1	1671.8085	K.^{294}TKPREEQY^{302}NSTYR^{306}.V	1710.5977	(Neu5Gc)Gal(GlcNAc)Man$_3$(GlcNAc)$_2$Fuc	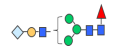
927.3839(4+)	3706.5121	3706.5118	0	1671.8085	K.^{294}TKPREEQY^{302}NSTYR^{306}.V	2034.7033	(Neu5Gc)Gal(GlcNAc)Man$_5$(GlcNAc)$_2$Fuc	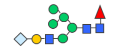
897.1269(4+)	3585.4841	3585.4855	0	1671.8085	K.^{294}TKPREEQY^{302}NSTYR^{306}.V	1913.6770	(Neu5Gc)Gal(GlcNAc)$_2$Man$_3$(GlcNAc)$_2$Fuc	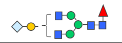
1195.8330(3+)	3585.4834	3585.4855	-1	1671.8085	K.^{294}TKPREEQY^{302}NSTYR^{306}.V	1913.6770	(Neu5Gc)Gal(GlcNAc)$_2$Man$_3$(GlcNAc)$_2$Fuc	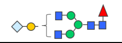
937.6404(4+)	3747.5381	3747.5383	0	1671.8085	K.^{294}TKPREEQY^{302}NSTYR^{306}.V	2075.7298	(Neu5Gc)Gal(GlcNAc)$_2$Man$_3$(GlcNAc)$_2$Fuc	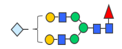
978.1534(4+)	3909.5901	3909.5912	0	1671.8085	K.^{294}TKPREEQY^{302}NSTYR^{306}.V	2237.7827	(Neu5Gc)Gal$_3$(GlcNAc)$_2$Man$_3$(GlcNAc)$_2$Fuc	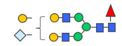
978.1526(4+)	3909.5869	3909.5912	-1	1671.8085	K.^{294}TKPREEQY^{302}NSTYR^{306}.V	2237.7827	(Neu5Gc)Gal$_3$(GlcNAc)$_2$Man$_3$(GlcNAc)$_2$Fuc	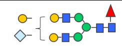
1120.1191(3+)	3358.3507	3358.3520	0	1671.8085	K.^{294}TKPREEQY^{302}NSTYR^{306}.V	1686.5435	Gal(GlcNAc)$_2$Man$_3$(GlcNAc)$_2$FucSO$_3$	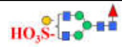
1174.1401(3+)	3520.4047	3520.4048	0	1671.8085	K.^{294}TKPREEQY^{302}NSTYR^{306}.V	1848.5963	Gal$_2$(GlcNAc)$_2$Man$_3$(GlcNAc)$_2$FucSO$_3$	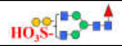
694.6143(3+)	2081.8273	2081.8292	-1	1189.5120	R.^{298}EEQY^{302}NSTYR^{306}.V	892.3172	Man$_3$(GlcNAc)$_2$	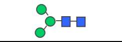
1041.4175(2+)	2081.8272	2081.8292	-1	1189.5120	R.^{298}EEQY^{302}NSTYR^{306}.V	892.3172	Man$_3$(GlcNAc)$_2$	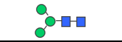
748.6321(3+)	2243.8807	2243.882	-1	1189.5120	R.^{298}EEQY^{302}NSTYR^{306}.V	1054.3700	Man$_4$(GlcNAc)$_2$	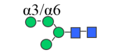
1122.4448(2+)	2243.8818	2243.8820	0	1189.5120	R.^{298}EEQY^{302}NSTYR^{306}.V	1054.3700	Man$_4$(GlcNAc)$_2$	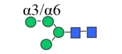
802.6494(3+)	2405.9326	2405.9349	-1	1189.5120	R.^{298}EEQY^{302}NSTYR^{306}.V	1216.4229	Man$_5$(GlcNAc)$_2$	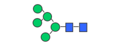
1203.4718(2+)	2405.9358	2405.9349	0	1189.5120	R.^{298}EEQY^{302}NSTYR^{306}.V	1216.4229	Man$_5$(GlcNAc)$_2$	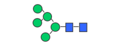
1446.5498(2+)	2892.0918	2892.0933	-1	1189.5120	R.^{298}EEQY^{302}NSTYR^{306}.V	1702.5813	Man$_8$(GlcNAc)$_2$	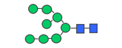
1276.4996(2+)	2551.9914	2551.9928	-1	1189.5120	R.^{298}EEQY^{302}NSTYR^{306}.V	1362.4808	Man$_5$(GlcNAc)$_2$Fuc	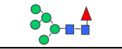
870.3422(3+)	2609.011	2609.0142	-1	1189.5120	R.^{298}EEQY^{302}NSTYR^{306}.V	1419.5022	(GlcNAc)Man$_5$(GlcNAc)$_2$	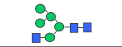
1305.0110(2+)	2609.0142	2609.0142	0	1189.5120	R.^{298}EEQY^{302}NSTYR^{306}.V	1419.5022	(GlcNAc)Man$_5$(GlcNAc)$_2$	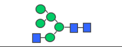
924.3608(3+)	2771.0668	2771.0671	0	1189.5120	R.^{298}EEQY^{302}NSTYR^{306}.V	1581.5551	Gal(GlcNAc)Man$_5$(GlcNAc)$_2$	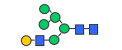

1386.0393(2+)	2771.0708	2771.0671	1	1189.5120	R.^{298}EEQY^{302}NSTYR^{306}.V	1581.5551	Gal(GlcNAc)Man$_5$(GlcNAc)$_2$	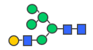
978.3774(3+)	2933.1166	2933.1199	-1	1189.5120	R.^{298}EEQY^{302}NSTYR^{306}.V	1743.6079	Gal(GlcNAc)Man$_6$(GlcNAc)$_2$	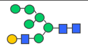
1467.0632(2+)	2933.1186	2933.1199	0	1189.5120	R.^{298}EEQY^{302}NSTYR^{306}.V	1743.6079	Gal(GlcNAc)Man$_6$(GlcNAc)$_2$	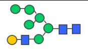
919.0282(3+)	2755.069	2755.0721	-1	1189.5120	R.^{298}EEQY^{302}NSTYR^{306}.V	1565.5601	(GlcNAc)Man$_5$(GlcNAc)$_2$Fuc	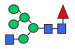
1378.0417(2+)	2755.0756	2755.0721	1	1189.5120	R.^{298}EEQY^{302}NSTYR^{306}.V	1565.5601	(GlcNAc)Man$_5$(GlcNAc)$_2$Fuc	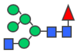
973.0490(3+)	2917.1314	2917.1249	2	1189.5120	R.^{298}EEQY^{302}NSTYR^{306}.V	1727.6129	Gal(GlcNAc)Man$_5$(GlcNAc)$_2$Fuc	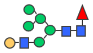
1459.0646(2+)	2917.1214	2917.1249	-1	1189.5120	R.^{298}EEQY^{302}NSTYR^{306}.V	1727.6129	Gal(GlcNAc)Man$_5$(GlcNAc)$_2$Fuc	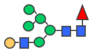
1027.0649(3+)	3079.1791	3079.1778	0	1189.5120	R.^{298}EEQY^{302}NSTYR^{306}.V	1889.6658	Gal(GlcNAc)Man$_6$(GlcNAc)$_2$Fuc / Gal$_2$(GlcNAc)Man$_5$(GlcNAc)$_2$Fuc	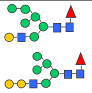
1540.0933(2+)	3079.1788	3079.1778	0	1189.5120	R.^{298}EEQY^{302}NSTYR^{306}.V	1889.6658	Gal(GlcNAc)Man$_6$(GlcNAc)$_2$Fuc / Gal$_2$(GlcNAc)Man$_5$(GlcNAc)$_2$Fuc	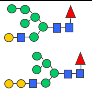
1142.9575(2+)	2284.9072	2284.9086	-1	1189.5120	R.^{298}EEQY^{302}NSTYR^{306}.V	1095.3966	(GlcNAc)Man$_3$(GlcNAc)$_2$	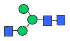
830.0000 (3+)	2487.9844	2487.988	-2	1189.5120	R.^{298}EEQY^{302}NSTYR^{306}.V	1298.4760	(GlcNAc)$_2$Man$_3$(GlcNAc)$_2$	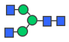
1244.4972(2+)	2487.9866	2487.9880	-1	1189.5120	R.^{298}EEQY^{302}NSTYR^{306}.V	1298.4760	(GlcNAc)$_2$Man$_3$(GlcNAc)$_2$	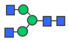
1223.9838(2+)	2446.9598	2446.9614	-1	1189.5120	R.^{298}EEQY^{302}NSTYR^{306}.V	1257.4494	(GlcNAc)Man$_4$(GlcNAc)$_2$	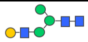
1134.9609(2+)	2268.914	2268.9135	0	1189.5120	R.^{298}EEQY^{302}NSTYR^{306}.V	1079.4015	(GlcNAc)Man$_2$(GlcNAc)$_2$Fuc	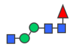
1215.9873(2+)	2430.9668	2430.9664	0	1189.5120	R.^{298}EEQY^{302}NSTYR^{306}.V	1241.4544	(GlcNAc)Man$_3$(GlcNAc)$_2$Fuc	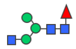
1317.5269(2+)	2634.0460	2634.0459	0	1189.5120	R.^{298}EEQY^{302}NSTYR^{306}.V	1444.5339	(GlcNAc)$_2$Man$_3$(GlcNAc)$_2$Fuc	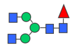
878.6876(3+)	2634.0472	2634.0459	1	1189.5120	R.^{298}EEQY^{302}NSTYR^{306}.V	1444.5339	(GlcNAc)$_2$Man$_3$(GlcNAc)$_2$Fuc	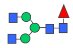
1297.0142(2+)	2593.0206	2593.0193	1	1189.5120	R.^{298}EEQY^{302}NSTYR^{306}.V	1403.5073	Gal(GlcNAc)Man$_3$(GlcNAc)$_2$Fuc	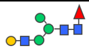
932.7050(3+)	2796.0994	2796.0987	0	1189.5120	R.^{298}EEQY^{302}NSTYR^{306}.V	1606.5867	Gal(GlcNAc)$_2$Man$_3$(GlcNAc)$_2$Fuc	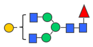
1398.5546(2+)	2796.1014	2796.0987	1	1189.5120	R.^{298}EEQY^{302}NSTYR^{306}.V	1606.5867	Gal(GlcNAc)$_2$Man$_3$(GlcNAc)$_2$Fuc	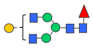
986.7223(3+)	2958.1513	2958.1515	0	1189.5120	R.^{298}EEQY^{302}NSTYR^{306}.V	1768.6395	Gal$_2$(GlcNAc)$_2$Man$_3$(GlcNAc)$_2$Fuc	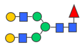
1479.5801(2+)	2958.1524	2958.1515	0	1189.5120	R.^{298}EEQY^{302}NSTYR^{306}.V	1768.6395	Gal$_2$(GlcNAc)$_2$Man$_3$(GlcNAc)$_2$Fuc	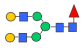
1040.7388(3+)	3120.2008	3120.2043	-1	1189.5120	R.^{298}EEQY^{302}NSTYR^{306}.V	1930.6923	Gal$_3$(GlcNAc)$_2$Man$_3$(GlcNAc)$_2$Fuc	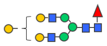
1560.6055(2+)	3120.2032	3120.2043	0	1189.5120	R.^{298}EEQY^{302}NSTYR^{306}.V	1930.6923	Gal$_3$(GlcNAc)$_2$Man$_3$(GlcNAc)$_2$Fuc	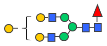
1641.6318(2+)	3282.2558	3282.2572	0	1189.5120	R.^{298}EEQY^{302}NSTYR^{306}.V	2092.7452	Gal$_4$(GlcNAc)$_2$Man$_3$(GlcNAc)$_2$Fuc	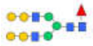
1094.7582(3+)	3282.259	3282.2572	1	1189.5120	R.^{298}EEQY^{302}NSTYR^{306}.V	2092.7452	Gal$_4$(GlcNAc)$_2$Man$_3$(GlcNAc)$_2$Fuc	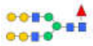
1369.5311(2+)	2738.0544	2738.0568	-1	1189.5120	R.^{298}EEQY^{302}NSTYR^{306}.V	1548.5448	(Neu5Ac)Gal(GlcNAc)Man$_3$(GlcNAc)$_2$	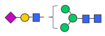
918.6889(3+)	2754.0511	2754.0517	0	1189.5120	R.^{298}EEQY^{302}NSTYR^{306}.V	1564.5397	(Neu5Gc)Gal(GlcNAc)Man$_3$(GlcNAc)$_2$	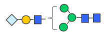
1377.5298(2+)	2754.0518	2754.0517	0	1189.5120	R.^{298}EEQY^{302}NSTYR^{306}.V	1564.5397	(Neu5Gc)Gal(GlcNAc)Man$_3$(GlcNAc)$_2$	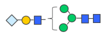
972.7065(3+)	2916.1039	2916.1046	0	1189.5120	R.^{298}EEQY^{302}NSTYR^{306}.V	1726.5926	(Neu5Gc)Gal(GlcNAc)Man$_4$(GlcNAc)$_2$	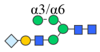
1458.5570(2+)	2916.1062	2916.1046	1	1189.5120	R.^{298}EEQY^{302}NSTYR^{306}.V	1726.5926	(Neu5Gc)Gal(GlcNAc)Man$_4$(GlcNAc)$_2$	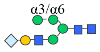

1026.7247(3+)	3078.1585	3078.1574	0	1189.5120	R.^{298}EEQY^{302}NSTYR^{306}.V	1888.6454	(Neu5Gc)Gal(GlcNAc)Man$_5$(GlcNAc)$_2$	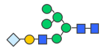
1539.5822(2+)	3078.1566	3078.1574	0	1189.5120	R.^{298}EEQY^{302}NSTYR^{306}.V	1888.6454	(Neu5Gc)Gal(GlcNAc)Man$_5$(GlcNAc)$_2$	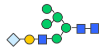
967.3745 (3+)	2900.1079	2900.1097	-1	1189.5120	R.^{298}EEQY^{302}NSTYR^{306}.V	1710.5977	(Neu5Gc)Gal(GlcNAc)Man$_3$(GlcNAc)$_2$Fuc	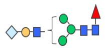
1450.5580(2+)	2900.1082	2900.1097	-1	1189.5120	R.^{298}EEQY^{302}NSTYR^{306}.V	1710.5977	(Neu5Gc)Gal(GlcNAc)Man$_3$(GlcNAc)$_2$Fuc	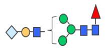
1021.3918(3+)	3062.1598	3062.1625	-1	1189.5120	R.^{298}EEQY^{302}NSTYR^{306}.V	1872.6505	(Neu5Gc)Gal(GlcNAc)Man$_4$(GlcNAc)$_2$Fuc	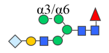
1531.5845(2+)	3062.1612	3062.1625	0	1189.5120	R.^{298}EEQY^{302}NSTYR^{306}.V	1872.6505	(Neu5Gc)Gal(GlcNAc)Man$_4$(GlcNAc)$_2$Fuc	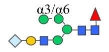
1075.4105(3+)	3224.2159	3224.2153	0	1189.5120	R.^{298}EEQY^{302}NSTYR^{306}.V	2034.7033	(Neu5Gc)Gal(GlcNAc)Man$_5$(GlcNAc)$_2$Fuc	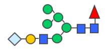
1612.6115(2+)	3224.2152	3224.2153	0	1189.5120	R.^{298}EEQY^{302}NSTYR^{306}.V	2034.7033	(Neu5Gc)Gal(GlcNAc)Man$_5$(GlcNAc)$_2$Fuc	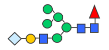
1035.0696(3+)	3103.1932	3103.189	1	1189.5120	R.^{298}EEQY^{302}NSTYR^{306}.V	1913.6770	(Neu5Gc)Gal(GlcNAc)$_2$Man$_3$(GlcNAc)$_2$Fuc	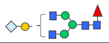
1552.1014(2+)	3103.1950	3103.1890	2	1189.5120	R.^{298}EEQY^{302}NSTYR^{306}.V	1913.6770	(Neu5Gc)Gal(GlcNAc)$_2$Man$_3$(GlcNAc)$_2$ Fuc	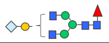
1089.0850(3+)	3265.2394	3265.2418	-1	1189.5120	R.^{298}EEQY^{302}NSTYR^{306}.V	2075.7298	(Neu5Gc)Gal(GlcNAc)$_2$Man$_3$(GlcNAc)$_2$Fuc	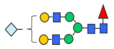
1633.1248(2+)	3265.2418	3265.2418	0	1189.5120	R.^{298}EEQY^{302}NSTYR^{306}.V	2075.7298	(Neu5Gc)Gal(GlcNAc)$_2$Man$_3$(GlcNAc)$_2$Fuc	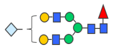
1143.1029(3+)	3427.2931	3427.2947	0	1189.5120	R.^{298}EEQY^{302}NSTYR^{306}.V	2237.7827	(Neu5Gc)Gal$_3$(GlcNAc)$_2$Man$_3$(GlcNAc)$_2$Fuc	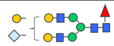
1191.4496 (3+)	3572.3332	3572.3322	0	1189.5120	R.^{298}EEQY^{302}NSTYR^{306}.V	2382.8202	(Neu5Gc)$_2$Gal$_2$(GlcNAc)$_2$Man$_3$(GlcNAc)$_2$Fuc	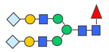
954.7283(3+)	2862.1693	2862.1681	0	1442.6659	K.^{296}PREEQY^{302}NSTYR^{306}.V	1419.5022	(GlcNAc)Man$_5$(GlcNAc)$_2$	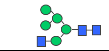
949.3959(3+)	2846.1721	2846.1732	0	1442.6659	K.^{296}PREEQY^{302}NSTYR^{306}.V	1403.5073	Gal(GlcNAc)Man$_3$(GlcNAc)$_2$Fuc	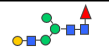
963.0709(3+)	2887.1971	2887.1998	-1	1442.6659	K.^{296}PREEQY^{302}NSTYR^{306}.V	1444.5339	(GlcNAc)$_2$Man$_3$(GlcNAc)$_2$Fuc	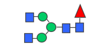
963.0717(3+)	2887.1995	2887.1998	0	1442.6659	K.^{296}PREEQY^{302}NSTYR^{306}.V	1444.5339	(GlcNAc)$_2$Man$_3$(GlcNAc)$_2$Fuc	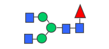
1017.0887(3+)	3049.2505	3049.2526	-1	1442.6659	K.^{296}PREEQY^{302}NSTYR^{306}.V	1606.5867	Gal(GlcNAc)$_2$Man$_3$(GlcNAc)$_2$Fuc	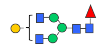
1071.1062(3+)	3211.303	3211.3054	-1	1442.6659	K.^{296}PREEQY^{302}NSTYR^{306}.V	1768.6395	Gal$_2$(GlcNAc)$_2$Man$_3$(GlcNAc)$_2$Fuc	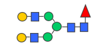
1119.4531(3+)	3356.3437	3356.3429	0	1442.6659	K.^{296}PREEQY^{302}NSTYR^{306}.V	1913.6770	(Neu5Gc)Gal(GlcNAc)$_2$Man$_3$(GlcNAc)$_2$Fuc	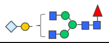
1051.7596(3+)	3153.2632	3153.2636	0	1442.6659	K.^{296}PREEQY^{302}NSTYR^{306}.V	1710.5977	(Neu5Gc)Gal(GlcNAc)Man$_3$(GlcNAc)$_2$Fuc	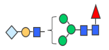

* The N-glycopeptides are determined by reversed-phase (RP) LC MS/MS of a tryptic Infliximab digest, and identified by database search using Byonic software.

§ Missed cleavages of Fc peptides at residues TKPREEQYNSTYR were also observed due to a low hydrolysis rate caused by the suppression of trypsin activity resulting from the co-existence of acidic residues (*i.e.* glutamic acid) and proline near the cleavage site (Rodriguez, J.; Gupta, N.; Smith, R. D.; Pevzner, P. A. *J Proteome Res.* **2008**, 7, 300-305).

\# Possible structures of N-glycans are predicted based on the initial database search against the in-house mAb glycan library by the accurate mass, glycan composition and the structures derived from the glycan biosynthesis pathway. The isomeric glycan structures are not distinguishable at the glycopeptide level.

Table S4. Identification of the tryptic glycopeptides of cetuximab derived from murine myeloma cells by reversed-phase LC MS/MS

Glycopeptide m/z (charge) *	Meas. MH+	Calc. MH+	ppm	Peptide MH+	Peptide sequence §	Glycan mass	Glycan composition	Putative glycan structure #
963.4149(3+)	2888.2291	2888.2314	-1	1671.8085	K.^{291}TKPREEQY^{299}NSTYR^{303}.V	1216.4229	Man$_5$(GlcNAc)$_2$	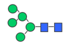
722.8134(4+)	2888.2301	2888.2314	0	1671.8085	K.^{291}TKPREEQY^{299}NSTYR^{303}.V	1216.4229	Man$_5$(GlcNAc)$_2$	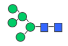
1444.6195(2+)	2888.2312	2888.2313	0	1671.8085	K.^{291}TKPREEQY^{299}NSTYR^{303}.V	1216.4229	Man$_5$(GlcNAc)$_2$	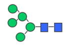
850.6108(4+)	3399.4197	3399.4214	-1	1671.8085	K.^{291}TKPREEQY^{299}NSTYR^{303}.V	1727.6129	Gal(GlcNAc)Man$_5$(GlcNAc)$_2$Fuc	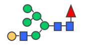
1133.8120(3+)	3399.4204	3399.4214	0	1671.8085	K.^{291}TKPREEQY^{299}NSTYR^{303}.V	1727.6129	Gal(GlcNAc)Man$_5$(GlcNAc)$_2$Fuc	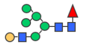
891.1239(4+)	3561.4721	3561.4743	-1	1671.8085	K.^{291}TKPREEQY^{299}NSTYR^{303}.V	1889.6658	Gal(GlcNAc)Man$_6$(GlcNAc)$_2$Fuc	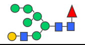
891.1239(4+)	3561.4721	3561.4743	-1	1671.8085	K.^{291}TKPREEQY^{299}NSTYR^{303}.V	1889.6658	Gal$_2$(GlcNAc)Man$_5$(GlcNAc)$_2$Fuc	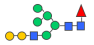
1187.8286(3+)	3561.4702	3561.4743	-1	1671.8085	K.^{291}TKPREEQY^{299}NSTYR^{303}.V	1889.6658	Gal(GlcNAc)Man$_6$(GlcNAc)$_2$Fuc	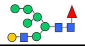
1187.8286(3+)	3561.4702	3561.4743	-1	1671.8085	K.^{291}TKPREEQY^{299}NSTYR^{303}.V	1889.6658	Gal$_2$(GlcNAc)Man$_5$(GlcNAc)$_2$Fuc	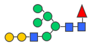
971.7589(3+)	2913.2611	2913.2630	-1	1671.8085	K.^{291}TKPREEQY^{299}NSTYR^{303}.V	1241.4545	(GlcNAc)Man$_3$(GlcNAc)$_2$Fuc	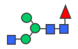
779.8416(4+)	3116.3429	3116.3424	0	1671.8085	K.^{291}TKPREEQY^{299}NSTYR^{303}.V	1444.5339	(GlcNAc)$_2$Man$_3$(GlcNAc)$_2$Fuc	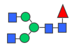
1039.4535(3+)	3116.3449	3116.3424	1	1671.8085	K.^{291}TKPREEQY^{299}NSTYR^{303}.V	1444.5339	(GlcNAc)$_2$Man$_3$(GlcNAc)$_2$Fuc	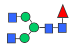
1025.7769(3+)	3075.3151	3075.3158	0	1671.8085	K.^{291}TKPREEQY^{299}NSTYR^{303}.V	1403.5073	Gal(GlcNAc)Man$_3$(GlcNAc)$_2$Fuc	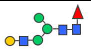
820.3544(4+)	3278.3941	3278.3952	0	1671.8085	K.^{291}TKPREEQY^{299}NSTYR^{303}.V	1606.5867	Gal(GlcNAc)$_2$Man$_3$(GlcNAc)$_2$Fuc	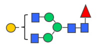
1093.4709(3+)	3278.3971	3278.3952	1	1671.8085	K.^{291}TKPREEQY^{299}NSTYR^{303}.V	1606.5867	Gal(GlcNAc)$_2$Man$_3$(GlcNAc)$_2$Fuc	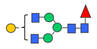
820.3550(4+)	3278.3965	3278.3952	0	1671.8085	K.^{291}TKPREEQY^{299}NSTYR^{303}.V	1606.5867	Gal(GlcNAc)$_2$Man$_3$(GlcNAc)$_2$Fuc	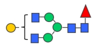
860.8677(4+)	3440.4473	3440.4480	0	1671.8085	K.^{291}TKPREEQY^{299}NSTYR^{303}.V	1768.6395	Gal$_2$(GlcNAc)$_2$Man$_3$(GlcNAc)$_2$Fuc	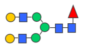
1147.4879(3+)	3440.4481	3440.448	0	1671.8085	K.^{291}TKPREEQY^{299}NSTYR^{303}.V	1768.6395	Gal$_2$(GlcNAc)$_2$Man$_3$(GlcNAc)$_2$Fuc	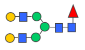
1201.5051(3+)	3602.4997	3602.5008	0	1671.8085	K.^{291}TKPREEQY^{299}NSTYR^{303}.V	1930.6923	Gal$_3$(GlcNAc)$_2$Man$_3$(GlcNAc)$_2$Fuc	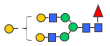
901.3807(4+)	3602.4993	3602.5008	0	1671.8085	K.^{291}TKPREEQY^{299}NSTYR^{303}.V	1930.6923	Gal$_3$(GlcNAc)$_2$Man$_3$(GlcNAc)$_2$Fuc	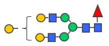
941.8935(4+)	3764.5505	3764.5537	-1	1671.8085	K.^{291}TKPREEQY^{299}NSTYR^{303}.V	2092.7452	Gal$_4$(GlcNAc)$_2$Man$_3$(GlcNAc)$_2$Fuc	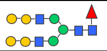
846.3577(4+)	3382.4073	3382.4061	0	1671.8085	K.^{291}TKPREEQY^{299}NSTYR^{303}.V	1710.5976	(Neu5Gc)Gal(GlcNAc)Man$_3$(GlcNAc)$_2$Fuc	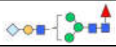
850.3563(4+)	3398.4017	3398.4011	0	1671.8085	K.^{291}TKPREEQY^{299}NSTYR^{303}.V	1726.5926	(Neu5Gc)Gal(GlcNAc)Man$_4$(GlcNAc)$_2$	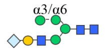
1041.4183(2+)	2081.8288	2081.8292	0	1189.5120	R.^{295}EEQY^{299}NSTYR^{303}.V	892.3172	Man$_3$(GlcNAc)$_2$	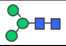
1122.4443(2+)	2243.8808	2243.8820	-1	1189.5120	R.^{295}EEQY^{299}NSTYR^{303}.V	1054.3700	Man$_4$(GlcNAc)$_2$	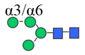
1203.4712(2+)	2405.9346	2405.9349	0	1189.5120	R.^{295}EEQY^{299}NSTYR^{303}.V	1216.4229	Man$_5$(GlcNAc)$_2$	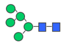
1284.4977(2+)	2567.9876	2567.9877	0	1189.5120	R.^{295}EEQY^{299}NSTYR^{303}.V	1378.4757	Man$_6$(GlcNAc)$_2$Fuc	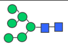
1195.4751(2+)	2389.9424	2389.9399	1	1189.5120	R.^{295}EEQY^{299}NSTYR^{303}.V	1200.4279	Man$_4$(GlcNAc)$_2$Fuc	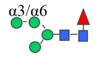
1276.5013(2+)	2551.9948	2551.9928	1	1189.5120	R.^{295}EEQY^{299}NSTYR^{303}.V	1362.4808	Man$_5$(GlcNAc)$_2$Fuc	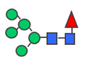
1386.0382(2+)	2771.0686	2771.0671	1	1189.5120	R.^{295}EEQY^{299}NSTYR^{303}.V	1581.5551	Gal(GlcNAc)Man$_5$(GlcNAc)$_2$	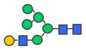
1378.0414(2+)	2755.075	2755.0721	1	1189.5120	R.^{295}EEQY^{299}NSTYR^{303}.V	1565.5601	(GlcNAc)Man$_5$(GlcNAc)$_2$Fuc	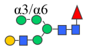

1378.0417(2+)	2755.0756	2755.0721	1	1189.5120	R.^{295}EEQY^{299}NSTYR^{303}.V	1565.5601	GlcNAcMan$_5$(GlcNAc)$_2$Fuc	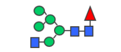
1378.0417(2+)	2755.0756	2755.0721	1	1189.5120	R.^{295}EEQY^{299}NSTYR^{303}.V	1565.5601	Gal$_2$(GlcNAc)Man$_3$(GlcNAc)$_2$Fuc	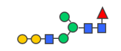
973.0466(3+)	2917.1242	2917.1248	0	1189.5120	R.^{295}EEQY^{299}NSTYR^{303}.V	1727.6128	Gal(GlcNAc)Man$_5$(GlcNAc)$_2$Fuc	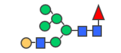
1459.0681(2+)	2917.1284	2917.1249	1	1189.5120	R.^{295}EEQY^{299}NSTYR^{303}.V	1727.6129	Gal(GlcNAc)Man$_5$(GlcNAc)$_2$Fuc	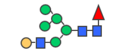
1027.0640(3+)	3079.1764	3079.1778	0	1189.5120	R.^{295}EEQY^{299}NSTYR^{303}.V	1889.6658	Gal(GlcNAc)Man$_6$(GlcNAc)$_2$Fuc	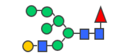
1027.0640(3+)	3079.1764	3079.1778	0	1189.5120	R.^{295}EEQY^{299}NSTYR^{303}.V	1889.6658	Gal$_2$(GlcNAc)Man$_5$(GlcNAc)$_2$Fuc	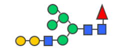
1540.0950(2+)	3079.1822	3079.1778	2	1189.5120	R.^{295}EEQY^{299}NSTYR^{303}.V	1889.6658	Gal(GlcNAc)Man$_6$(GlcNAc)$_2$Fuc	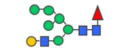
1540.0950(2+)	3079.1822	3079.1778	2	1189.5120	R.^{295}EEQY^{299}NSTYR^{303}.V	1889.6658	Gal$_2$(GlcNAc)Man$_5$(GlcNAc)$_2$Fuc	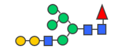
1142.9573(2+)	2284.9068	2284.9086	-1	1189.5120	R.^{295}EEQY^{299}NSTYR^{303}.V	1095.3966	(GlcNAc)Man$_3$(GlcNAc)$_2$	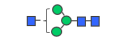
1244.4971(2+)	2487.9864	2487.9880	-1	1189.5120	R.^{295}EEQY^{299}NSTYR^{303}.V	1298.4760	(GlcNAc)$_2$Man$_3$(GlcNAc)$_2$	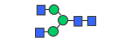
1134.9604(2+)	2268.9130	2268.9136	0	1189.5120	R.^{295}EEQY^{299}NSTYR^{303}.V	1079.4016	(GlcNAc)Man$_2$(GlcNAc)$_2$Fuc	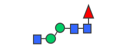
1215.9869(2+)	2430.966	2430.9665	0	1189.5120	R.^{295}EEQY^{299}NSTYR^{303}.V	1241.4545	(GlcNAc)Man$_3$(GlcNAc)$_2$Fuc	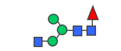
878.6876(3+)	2634.0472	2634.0459	1	1189.5120	R.^{295}EEQY^{299}NSTYR^{303}.V	1444.5339	(GlcNAc)$_2$Man$_3$(GlcNAc)$_2$Fuc	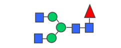
1317.5271(2+)	2634.0464	2634.0459	0	1189.5120	R.^{295}EEQY^{299}NSTYR^{303}.V	1444.5339	(GlcNAc)$_2$Man$_3$(GlcNAc)$_2$Fuc	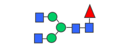
1297.0139(2+)	2593.0200	2593.0193	0	1189.5120	R.^{295}EEQY^{299}NSTYR^{303}.V	1403.5073	Gal(GlcNAc)Man$_3$(GlcNAc)$_2$Fuc	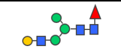
932.7061(3+)	2796.1027	2796.0987	2	1189.5120	R.^{295}EEQY^{299}NSTYR^{303}.V	1606.5867	Gal(GlcNAc)$_2$Man$_3$(GlcNAc)$_2$Fuc	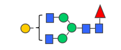
1398.5530(2+)	2796.0982	2796.0987	0	1189.5120	R.^{295}EEQY^{299}NSTYR^{303}.V	1606.5867	Gal(GlcNAc)$_2$Man$_3$(GlcNAc)$_2$Fuc	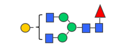
986.7228(3+)	2958.1528	2958.1515	0	1189.5120	R.^{295}EEQY^{299}NSTYR^{303}.V	1768.6395	Gal$_2$(GlcNAc)$_2$Man$_3$(GlcNAc)$_2$Fuc	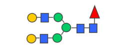
1479.5795(2+)	2958.1512	2958.1515	0	1189.5120	R.^{295}EEQY^{299}NSTYR^{303}.V	1768.6395	Gal$_2$(GlcNAc)$_2$Man$_3$(GlcNAc)$_2$Fuc	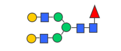
1040.7404(3+)	3120.2056	3120.2043	0	1189.5120	R.^{295}EEQY^{299}NSTYR^{303}.V	1930.6923	Gal$_3$(GlcNAc)$_2$Man$_3$(GlcNAc)$_2$Fuc	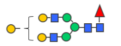
1094.7570(3+)	3282.2554	3282.2572	-1	1189.5120	R.^{295}EEQY^{299}NSTYR^{303}.V	2092.7452	Gal$_4$(GlcNAc)$_2$Man$_3$(GlcNAc)$_2$Fuc	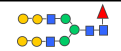
1641.6338(2+)	3282.2598	3282.2572	1	1189.5120	R.^{295}EEQY^{299}NSTYR^{303}.V	2092.7452	Gal$_4$(GlcNAc)$_2$Man$_3$(GlcNAc)$_2$Fuc	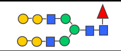
972.7073(3+)	2916.1063	2916.1046	1	1189.5120	R.^{295}EEQY^{299}NSTYR^{303}.V	1726.5926	(Neu5Gc)Gal(GlcNAc)Man$_4$(GlcNAc)$_2$	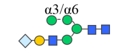
1026.7247(3+)	3078.1585	3078.1574	0	1189.5120	R.^{295}EEQY^{299}NSTYR^{303}.V	1888.6454	(Neu5Gc)Gal(GlcNAc)Man$_5$(GlcNAc)$_2$	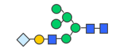
967.3755(3+)	2900.1109	2900.1097	0	1189.5120	R.^{295}EEQY^{299}NSTYR^{303}.V	1710.5977	(Neu5Gc)Gal(GlcNAc)Man$_3$(GlcNAc)$_2$Fuc	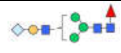
1450.5599(2+)	2900.1120	2900.1097	1	1189.5120	R.^{295}EEQY^{299}NSTYR^{303}.V	1710.5977	(Neu5Gc)Gal(GlcNAc)Man$_3$(GlcNAc)$_2$Fuc	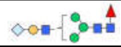
1021.3926(3+)	3062.1622	3062.1625	0	1189.5120	R.^{295}EEQY^{299}NSTYR^{303}.V	1872.6505	(Neu5Gc)Gal(GlcNAc)Man$_4$(GlcNAc)$_2$Fuc	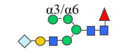
1531.5880(2+)	3062.1682	3062.1625	2	1189.5120	R.^{295}EEQY^{299}NSTYR^{303}.V	1872.6505	(Neu5Gc)Gal(GlcNAc)Man$_4$(GlcNAc)$_2$Fuc	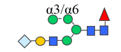
1075.4098(3+)	3224.2138	3224.2153	0	1189.5120	R.^{295}EEQY^{299}NSTYR^{303}.V	2034.7033	(Neu5Gc)Gal(GlcNAc)Man$_5$(GlcNAc)$_2$Fuc	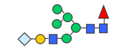
1089.0858(3+)	3265.2418	3265.2418	0	1189.5120	R.^{295}EEQY^{299}NSTYR^{303}.V	2075.7298	(Neu5Gc)Gal$_2$(GlcNAc)$_2$Man$_3$(GlcNAc)$_2$Fuc	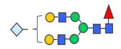
1633.1262(2+)	3265.2446	3265.2418	1	1189.5120	R.^{295}EEQY^{299}NSTYR^{303}.V	2075.7298	(Neu5Gc)Gal$_2$(GlcNAc)$_2$Man$_3$(GlcNAc)$_2$Fuc	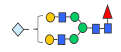
1143.1034(3+)	3427.2946	3427.2947	0	1189.5120	R.^{295}EEQY^{299}NSTYR^{303}.V	2237.7827	(Neu5Gc)Gal$_3$(GlcNAc)$_2$Man$_3$(GlcNAc)$_2$Fuc	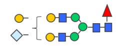

1137.77277(3+)	3411.3025	3411.2997	1	1189.5120	R.^{295}EEQY^{299}NSTYR^{303}.V	2221.7877	(Neu5Gc)Gal$_2$(GlcNAc)$_2$FucMan$_3$(GlcNAc)$_2$ Fuc	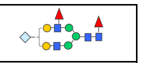
1041.7604(3+)	3123.2656	3123.2651	0	1906.8422	K.^{82}MNSLQS^{88}NDTAIYYCAR^{97}.A	1216.4229	Man$_5$(GlcNAc)$_2$	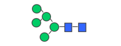
1562.1359(2+)	3123.264	3123.2651	0	1906.8422	K.^{82}MNSLQS^{88}NDTAIYYCAR^{97}.A	1216.4229	Man$_5$(GlcNAc)$_2$	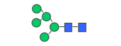
1212.1577(3+)	3634.4575	3634.4551	1	1906.8422	K.^{82}MNSLQS^{88}NDTAIYYCAR^{97}.A	1727.6129	Gal(GlcNAc)Man$_5$(GlcNAc)$_2$Fuc	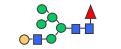
1266.1753(3+)	3796.5103	3796.5080	1	1906.8422	K.^{82}MNSLQS^{88}NDTAIYYCAR^{97}.A	1889.6658	Gal$_2$(GlcNAc)Man$_5$(GlcNAc)$_2$Fuc	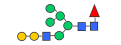
1684.1915(2+)	3367.3752	3367.3710	1	1906.8422	K.^{82}MNSLQS^{88}NDTAIYYCAR^{97}.A	1460.5288	Gal(GlcNAc)$_2$Man$_3$(GlcNAc)$_2$	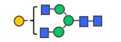
1655.6782(2+)	3310.3486	3310.3495	0	1906.8422	K.^{82}MNSLQS^{88}NDTAIYYCAR^{97}.A	1403.5073	Gal(GlcNAc)Man$_3$(GlcNAc)$_2$Fuc	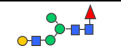
1158.1404(3+)	3472.4056	3472.4023	1	1906.8422	K.^{82}MNSLQS^{88}NDTAIYYCAR^{97}.A	1565.5601	(GlcNAc)Man$_5$(GlcNAc)$_2$Fuc	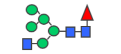
1158.1404(3+)	3472.4056	3472.4023	1	1906.8422	K.^{82}MNSLQS^{88}NDTAIYYCAR^{97}.A	1565.5601	Gal$_2$(GlcNAc)Man$_3$(GlcNAc)$_2$Fuc	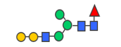
1574.6547(2+)	3148.3016	3148.2967	2	1906.8422	K.^{82}MNSLQS^{88}NDTAIYYCAR^{97}.A	1241.4545	(GlcNAc)Man$_3$(GlcNAc)$_2$Fuc	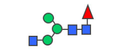
1117.7979(3+)	3351.3781	3351.3761	1	1906.8422	K.^{82}MNSLQS^{88}NDTAIYYCAR^{97}.A	1444.5339	(GlcNAc)$_2$Man$_3$(GlcNAc)$_2$Fuc	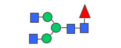
1676.1929(2+)	3351.378	3351.3761	1	1906.8422	K.^{82}MNSLQS^{88}NDTAIYYCAR^{97}.A	1444.5339	(GlcNAc)$_2$Man$_3$(GlcNAc)$_2$Fuc	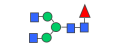
1104.1221(3+)	3310.3507	3310.3495	0	1906.8422	K.^{82}MNSLQS^{88}NDTAIYYCAR^{97}.A	1403.5073	Gal(GlcNAc)Man$_3$(GlcNAc)$_2$Fuc	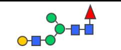
1171.8149(3+)	3513.4291	3513.4289	0	1906.8422	K.^{82}MNSLQS^{88}NDTAIYYCAR^{97}.A	1606.5867	Gal(GlcNAc)$_2$Man$_3$(GlcNAc)$_2$Fuc	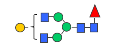
1225.8333(3+)	3675.4843	3675.4817	1	1906.8422	K.^{82}MNSLQS^{88}NDTAIYYCAR^{97}.A	1768.6395	Gal$_2$(GlcNAc)$_2$Man$_3$(GlcNAc)$_2$Fuc	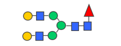
1279.8512(3+)	3837.5380	3837.5345	1	1906.8422	K.^{82}MNSLQS^{88}NDTAIYYCAR^{97}.A	1930.6923	Gal$_3$(GlcNAc)$_2$Man$_3$(GlcNAc)$_2$Fuc	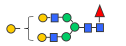
1333.8687(3+)	3999.5905	3999.5874	1	1906.8422	K.^{82}MNSLQS^{88}NDTAIYYCAR^{97}.A	2092.7452	Gal$_4$(GlcNAc)$_2$Man$_3$(GlcNAc)$_2$Fuc	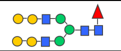
1401.227(3+)	4201.6525	4201.6464	2	1963.8637	K.^{82}MNSLQS^{88}NDTAIYYCAR^{97}.A S-carbamidomethylation of methionine (+57.0215 Da) and cysteine (+57.0215 Da)	2237.7827	(Neu5Gc)Gal$_3$(GlcNAc)$_2$Man$_3$(GlcNAc)$_2$Fuc	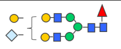
1401.5500(3+)	4202.6344	4202.6304	1	1964.8477	K.^{82}MNSLQS^{88}NDTAIYYCAR^{97}.A S-carbamidomethylation of methionine (+57.0215 Da) and cysteine (+57.0215 Da), and deamidation of asparagine (+0.9840 Da)	2237.7827	(Neu5Gc)Gal$_3$(GlcNAc)$_2$Man$_3$(GlcNAc)$_2$Fuc	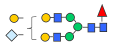
1401.5633(3+)	4202.6743	4202.6667	2	1906.8422	K.^{82}MNSLQS^{88}NDTAIYYCAR^{97}.A	2295.8245	Gal$_4$(GlcNAc)$_3$Man$_3$(GlcNAc)$_2$Fuc	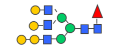
1455.5795(3+)	4364.7229	4364.7196	1	1906.8422	K.^{82}MNSLQS^{88}NDTAIYYCAR^{97}.A	2457.8774	Gal$_5$(GlcNAc)$_3$Man$_3$(GlcNAc)$_2$Fuc	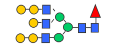
1132.4512(4+)	4526.7813	4526.7724	2	1906.8422	K.^{82}MNSLQS^{88}NDTAIYYCAR^{97}.A	2619.9302	Gal$_6$(GlcNAc)$_3$Man$_3$(GlcNAc)$_2$Fuc	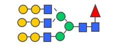
1509.5977(3+)	4526.7775	4526.7724	1	1906.8422	K.^{82}MNSLQS^{88}NDTAIYYCAR^{97}.A	2619.9302	Gal$_6$(GlcNAc)$_3$Man$_3$(GlcNAc)$_2$Fuc	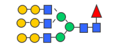
1206.4850(3+)	3617.4394	3617.4399	0	1906.8422	K.^{82}MNSLQS^{88}NDTAIYYCAR^{97}.A	1710.5977	(Neu5Gc)Gal(GlcNAc)Man$_3$(GlcNAc)$_2$Fuc	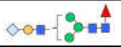
1274.1798(3+)	3820.5238	3820.5192	1	1906.8422	K.^{82}MNSLQS^{88}NDTAIYYCAR^{97}.A	1913.6770	(Neu5Gc)Gal(GlcNAc)$_2$Man$_3$(GlcNAc)$_2$Fuc	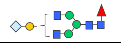
1328.1968(3+)	3982.5748	3982.5720	1	1906.8422	K.^{82}MNSLQS^{88}NDTAIYYCAR^{97}.A	2075.7298	(Neu5Gc)Gal(GlcNAc)$_2$Man$_3$(GlcNAc)$_2$Fuc	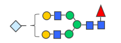
1328.1960(3+)	3982.5724	3982.5608	3	1889.8156	K.^{82}MNSLQS^{88}NDTAIYYCAR^{97}.A (Neutral loss of ammonia, -17.0266 Da)	2092.7452	Gal$_4$(GlcNAc)$_2$Man$_3$(GlcNAc)$_2$Fuc	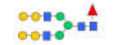
1376.8828(3+)	4128.6328	4128.6299	1	1906.8422	K.^{82}MNSLQS^{88}NDTAIYYCAR^{97}.A	2221.7877	(Neu5Ac)Gal$_3$(GlcNAc)$_2$Man$_3$(GlcNAc)$_2$Fuc	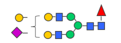
1382.2145(3+)	4144.6279	4144.6249	1	1906.8422	K.^{82}MNSLQS^{88}NDTAIYYCAR^{97}.A	2237.7827	(Neu5Gc)Gal$_3$(GlcNAc)$_2$Man$_3$(GlcNAc)$_2$Fuc	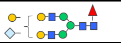
1036.9132(4+)	4144.6293	4144.6249	1	1906.8422	K.^{82}MNSLQS^{88}NDTAIYYCAR^{97}.A	2237.7827	(Neu5Gc)Gal$_3$(GlcNAc)$_2$Man$_3$(GlcNAc)$_2$Fuc	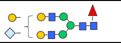
1430.5612(3+)	4289.668	4289.6624	1	1906.8422	K.^{82}MNSLQS^{88}NDTAIYYCAR^{97}.A	2382.8202	(Neu5Gc)$_2$Gal$_2$(GlcNAc)$_2$Man$_3$(GlcNAc)$_2$Fuc	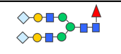
1073.1707(4+)	4289.6593	4289.6624	-1	1906.8422	K.^{82}MNSLQS^{88}NDTAIYYCAR^{97}.A	2382.8202	(Neu5Gc)$_2$Gal$_2$(GlcNAc)$_2$Man$_3$(GlcNAc)$_2$Fuc	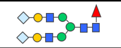
1328.5364(3+)	3983.5936	3983.5924	0	1906.8422	K.^{82}MNSLQS^{88}NDTAIYYCAR^{97}.A	2076.7502	Gal$_3$(GlcNAc)$_2$FucMan$_3$(GlcNAc)$_2$Fuc	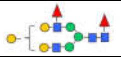

1328.5249(3+)	3983.5591	3983.5560	1	1907.8262	K.^{82}MNSLQS^{88}NDTAIYYCAR^{97}.A<br>Deamidation of asparagine (+0.9840 Da)	2075.7298	(Neu5Gc)Gal(GlcNAc)$_2$Man$_3$(GlcNAc)$_2$Fuc	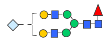
1382.5421(3+)	4145.6107	4145.6089	0	1907.8262	K.^{82}MNSLQS^{88}NDTAIYYCAR^{97}.A<br>Deamidation of asparagine (+0.9840 Da)	2237.7827	(Neu5Gc)Gal$_3$(GlcNAc)$_2$Man$_3$(GlcNAc)$_2$Fuc	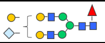
1376.8828(3+)	4128.6328	4128.8422	1	1906.8422	K.^{82}MNSLQS^{88}NDTAIYYCAR^{97}.A	2221.7877	(Neu5Gc)Gal$_2$(GlcNAc)$_2$FucMan$_3$(GlcNAc)$_2$ Fuc	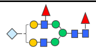

* The N-glycopeptides are determined by reversed-phase (RP) LC MS/MS of a tryptic Cetuximab digest, and identified by database search using Byonic software.

§ Missed cleavages of Fc peptides at residues TKPREEQYNSTYR were also observed due to a low hydrolysis rate caused by the suppression of trypsin activity resulting from the co-existence of acidic residues (*i.e.* glutamic acid) and proline near the cleavage site (Rodriguez, J.; Gupta, N.; Smith, R. D.; Pevzner, P. A. *J Proteome Res.* **2008**, 7, 300-305).

\# Possible structures of N-glycans are predicted based on the initial database search against the in-house mAb glycan library by the accurate mass, glycan composition and the structures derived from the glycan biosynthesis pathway. The isomeric glycan structures are not distinguishable at the glycopeptide level.

Table S5. Identification of the tryptic glycopeptides of golimumab derived from murine myelama cells by reversed-phase LC MS/MS

Glycopeptide *m/z* (charge) *	Meas. MH+	Calc. MH+	ppm	Peptide MH+	Peptide sequence §	Glycan mass	Glycan composition	Putative glycan structure #
1109.9695(2+)	2218.9312	2218.9344	-2	1164.5644	K.^{298}TKPREEQY^{306}N^{307}.S	1054.3700	Man$_4$(GlcNAc)$_2$	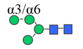
794.3325(3+)	2380.9819	2380.9873	-2	1164.5644	K.^{298}TKPREEQY^{306}N^{307}.S	1216.4229	Man$_5$(GlcNAc)$_2$	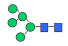
1190.9948(2+)	2380.9818	2380.9873	-2	1164.5644	K.^{298}TKPREEQY^{306}N^{307}.S	1216.4229	Man$_5$(GlcNAc)$_2$	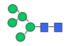
862.0252(3+)	2584.0600	2584.0666	-3	1164.5644	K.^{298}TKPREEQY^{306}N^{307}.S	1419.5022	(GlcNAc)Man$_5$(GlcNAc)$_2$	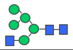
916.0431(3+)	2746.1137	2746.1195	-2	1164.5644	K.^{298}TKPREEQY^{306}N^{307}.S	1581.5551	Gal(GlcNAc)Man$_5$(GlcNAc)$_2$	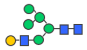
970.0605(3+)	2908.1659	2908.1723	-2	1164.5644	K.^{298}TKPREEQY^{306}N^{307}.S	1743.6079	Gal(GlcNAc)Man$_6$(GlcNAc)$_2$	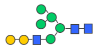
1365.5658(2+)	2730.1238	2730.1245	0	1164.5644	K.^{298}TKPREEQY^{306}N^{307}.S	1565.5601	(GlcNAc)Man$_5$(GlcNAc)$_2$Fuc	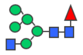
1305.0504(2+)	2609.093	2609.0983	-2	1164.5644	K.^{298}TKPREEQY^{306}N^{307}.S	1444.5339	(GlcNAc)$_2$Man$_3$(GlcNAc)$_2$Fuc	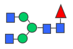
1386.0771(2+)	2771.1464	2771.1511	-2	1164.5644	K.^{298}TKPREEQY^{306}N^{307}.S	1606.5867	Gal(GlcNAc)$_2$Man$_3$(GlcNAc)$_2$Fuc	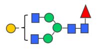
1467.1028(2+)	2933.1978	2933.2039	-2	1164.5644	K.^{298}TKPREEQY^{306}N^{307}.S	1768.6395	Gal$_2$(GlcNAc)$_2$Man$_3$(GlcNAc)$_2$Fuc	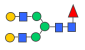
1122.4846(2+)	2243.9614	2243.9661	-2	1164.5644	K.^{298}TKPREEQY^{306}N^{307}.S	1079.4017	(GlcNAc)Man$_2$(GlcNAc)$_2$Fuc	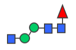
1284.5372(2+)	2568.0666	2568.0717	-2	1164.5644	K.^{298}TKPREEQY^{306}N^{307}.S	1403.5073	Gal(GlcNAc)Man$_3$(GlcNAc)$_2$Fuc	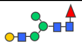
1203.5110(2+)	2406.0142	2406.0189	-2	1164.5644	K.^{298}TKPREEQY^{306}N^{307}.S	1241.4545	(GlcNAc)Man$_3$(GlcNAc)$_2$Fuc	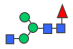
870.3699(3+)	2609.0941	2609.0983	-2	1164.5644	K.^{298}TKPREEQY^{306}N^{307}.S	1444.5339	(GlcNAc)$_2$Man$_3$(GlcNAc)$_2$Fuc	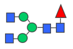
924.3882(3+)	2771.149	2771.1511	-1	1164.5644	K.^{298}TKPREEQY^{306}N^{307}.S	1606.5867	Gal(GlcNAc)$_2$Man$_3$(GlcNAc)$_2$Fuc	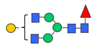
978.4047(3+)	2933.1985	2933.2039	-2	1164.5644	K.^{298}TKPREEQY^{306}N^{307}.S	1768.6395	Gal$_2$(GlcNAc)$_2$Man$_3$(GlcNAc)$_2$Fuc	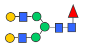
1032.4221(3+)	3095.2507	3095.2567	-2	1164.5644	K.^{298}TKPREEQY^{306}N^{307}.S	1930.6923	Gal$_3$(GlcNAc)$_2$Man$_3$(GlcNAc)$_2$Fuc	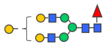
1086.4397(3+)	3257.3035	3257.3096	-2	1164.5644	K.^{298}TKPREEQY^{306}N^{307}.S	2092.7452	Gal$_4$(GlcNAc)$_2$Man$_3$(GlcNAc)$_2$Fuc	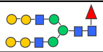
1018.4064(3+)	3053.2036	3053.2098	-2	1164.5644	K.^{298}TKPREEQY^{306}N^{307}.S	1888.6454	(Neu5Gc)Gal(GlcNAc)Man$_5$(GlcNAc)$_2$	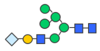
1067.0918(3+)	3199.2598	3199.2677	-3	1164.5644	K.^{298}TKPREEQY^{306}N^{307}.S	2034.7033	(Neu5Gc)Gal(GlcNAc)Man$_5$(GlcNAc)$_2$Fuc	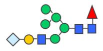
1134.7859(3+)	3402.3421	3402.3471	-2	1164.5644	K.^{298}TKPREEQY^{306}N^{307}.S	2237.7827	(Neu5Gc)Gal$_3$(GlcNAc)$_2$Man$_3$(GlcNAc)$_2$ Fuc	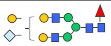
1282.5626(2+)	2564.1174	2564.1257	-3	1671.8085	K.^{298}TKPREEQY^{306}NSTYR^{310}.V	892.3172	Man$_3$(GlcNAc)$_2$	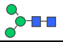
1363.5908(2+)	2726.1738	2726.1785	-2	1671.8085	K.^{298}TKPREEQY^{306}NSTYR^{310}.V	1054.3700	Man$_4$(GlcNAc)$_2$	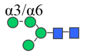
1444.6176(2+)	2888.2274	2888.2314	-1	1671.8085	K.^{298}TKPREEQY^{306}NSTYR^{310}.V	1216.4229	Man$_5$(GlcNAc)$_2$	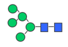
1017.4318(3+)	3050.2798	3050.2842	-2	1671.8085	K.^{298}TKPREEQY^{306}NSTYR^{310}.V	1378.4757	Man$_6$(GlcNAc)$_2$	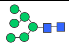
1071.4493(3+)	3212.3323	3212.337	-2	1671.8085	K.^{298}TKPREEQY^{306}NSTYR^{310}.V	1540.5285	Man$_7$(GlcNAc)$_2$	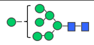
1125.4667(3+)	3374.3845	3374.3898	-2	1671.8085	K.^{298}TKPREEQY^{306}NSTYR^{310}.V	1702.5813	Man$_8$(GlcNAc)$_2$	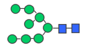
1627.1832(2+)	3253.3586	3253.3636	-2	1671.8085	K.^{298}TKPREEQY^{306}NSTYR^{310}.V	1581.5551	Gal(GlcNAc)Man$_5$(GlcNAc)$_2$	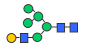
1139.1421(3+)	3415.4107	3415.4164	-2	1671.8085	K.^{298}TKPREEQY^{306}NSTYR^{310}.V	1743.6079	Gal(GlcNAc)Man$_6$(GlcNAc)$_2$	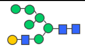
1031.1071(3+)	3091.3057	3091.3107	-2	1671.8085	K.^{298}TKPREEQY^{306}NSTYR^{310}.V	1419.5022	(GlcNAc)Man$_5$(GlcNAc)$_2$	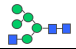
1546.1576(2+)	3091.3074	3091.3107	-1	1671.8085	K.^{298}TKPREEQY^{306}NSTYR^{310}.V	1419.5022	(GlcNAc)Man$_5$(GlcNAc)$_2$	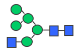

1627.1831(2+)	3253.3584	3253.3636	-2	1671.8085	K.^{298}TKPREEQY^{306}NSTYR^{310}.V	1581.5551	Gal(GlcNAc)Man$_5$(GlcNAc)$_2$	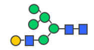
1085.1248(3+)	3253.3588	3253.3636	-2	1671.8085	K.^{298}TKPREEQY^{306}NSTYR^{310}.V	1581.5551	Gal(GlcNAc)Man$_5$(GlcNAc)$_2$	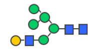
1303.0770(2+)	2605.1462	2605.1523	-2	1671.8085	K.^{298}TKPREEQY^{306}NSTYR^{310}.V	933.3438	(GlcNAc)Man$_2$(GlcNAc)$_2$	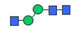
743.3244(4+)	2970.2741	2970.2845	-4	1671.8085	K.^{298}TKPREEQY^{306}NSTYR^{310}.V	1298.4760	(GlcNAc)$_2$Man$_3$(GlcNAc)$_2$	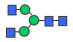
990.7639(3+)	2970.2761	2970.2845	-3	1671.8085	K.^{298}TKPREEQY^{306}NSTYR^{310}.V	1298.4760	(GlcNAc)$_2$Man$_3$(GlcNAc)$_2$	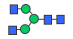
1485.6436(2+)	2970.2794	2970.2845	-2	1671.8085	K.^{298}TKPREEQY^{306}NSTYR^{310}.V	1298.4760	(GlcNAc)$_2$Man$_3$(GlcNAc)$_2$	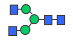
1566.6709(2+)	3132.3340	3132.3373	-1	1671.8085	K.^{298}TKPREEQY^{306}NSTYR^{310}.V	1460.5288	Gal(GlcNAc)$_2$Man$_3$(GlcNAc)$_2$	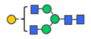
917.7389(3+)	2751.2011	2751.2102	-4	1671.8085	K.^{298}TKPREEQY^{306}NSTYR^{310}.V	1079.4017	(GlcNAc)Man$_2$(GlcNAc)$_2$Fuc	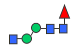
971.7565(3+)	2913.2539	2913.263	-3	1671.8085	K.^{298}TKPREEQY^{306}NSTYR^{310}.V	1241.4545	(GlcNAc)Man$_3$(GlcNAc)$_2$Fuc	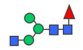
1025.7759(3+)	3075.3121	3075.3158	-1	1671.8085	K.^{298}TKPREEQY^{306}NSTYR^{310}.V	1403.5073	Gal(GlcNAc)Man$_3$(GlcNAc)$_2$Fuc	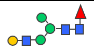
1538.1583(2+)	3075.3088	3075.3158	-2	1671.8085	K.^{298}TKPREEQY^{306}NSTYR^{310}.V	1403.5073	Gal(GlcNAc)Man$_3$(GlcNAc)$_2$Fuc	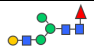
1079.7935(3+)	3237.3649	3237.3686	-1	1671.8085	K.^{298}TKPREEQY^{306}NSTYR^{310}.V	1565.5601	(GlcNAc)Man$_5$(GlcNAc)$_2$Fuc	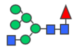
1619.1855(2+)	3237.3632	3237.3686	-2	1671.8085	K.^{298}TKPREEQY^{306}NSTYR^{310}.V	1565.5601	(GlcNAc)Man$_5$(GlcNAc)$_2$Fuc	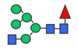
1133.8105(3+)	3399.4294	3399.4214	2	1671.8085	K.^{298}TKPREEQY^{306}NSTYR^{310}.V	1727.6129	Gal(GlcNAc)Man$_5$(GlcNAc)$_2$Fuc	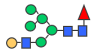
1187.8282(3+)	3561.469	3561.4743	-2	1671.8085	K.^{298}TKPREEQY^{306}NSTYR^{310}.V	1889.6658	Gal(GlcNAc)Man$_6$(GlcNAc)$_2$Fuc	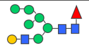
1187.8282(3+)	3561.469	3561.4743	-2	1671.8085	K.^{298}TKPREEQY^{306}NSTYR^{310}.V	1889.6658	Gal$_2$(GlcNAc)Man$_5$(GlcNAc)$_2$Fuc	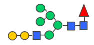
1355.5928(2+)	2710.1778	2710.1836	-2	1671.8085	K.^{298}TKPREEQY^{306}NSTYR^{310}.V	1038.3751	Man$_3$(GlcNAc)$_2$Fuc	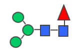
1376.1058(2+)	2751.2038	2751.2102	-2	1671.8085	K.^{298}TKPREEQY^{306}NSTYR^{310}.V	1079.4017	(GlcNAc)Man$_2$(GlcNAc)$_2$Fuc	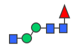
1457.1327(2+)	2913.2576	2913.2629	-2	1671.8085	K.^{298}TKPREEQY^{306}NSTYR^{310}.V	1241.4544	(GlcNAc)Man$_3$(GlcNAc)$_2$Fuc	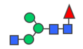
779.8397(4+)	3116.3353	3116.3424	-2	1671.8085	K.^{298}TKPREEQY^{306}NSTYR^{310}.V	1444.5339	(GlcNAc)$_2$Man$_3$(GlcNAc)$_2$Fuc	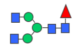
1039.4498(3+)	3116.3338	3116.3424	-3	1671.8085	K.^{298}TKPREEQY^{306}NSTYR^{310}.V	1444.5339	(GlcNAc)$_2$Man$_3$(GlcNAc)$_2$Fuc	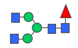
1558.6748(2+)	3116.3418	3116.3424	0	1671.8085	K.^{298}TKPREEQY^{306}NSTYR^{310}.V	1444.5339	(GlcNAc)$_2$Man$_3$(GlcNAc)$_2$Fuc	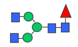
1039.4509(3+)	3116.3371	3116.3424	-2	1671.8085	K.^{298}TKPREEQY^{306}NSTYR^{310}.V	1444.5339	(GlcNAc)$_2$Man$_3$(GlcNAc)$_2$Fuc	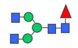
1538.1600(2+)	3075.3122	3075.3158	-1	1671.8085	K.^{298}TKPREEQY^{306}NSTYR^{310}.V	1403.5073	Gal(GlcNAc)Man$_3$(GlcNAc)$_2$Fuc	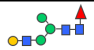
1025.7755(3+)	3075.3109	3075.3158	-2	1671.8085	K.^{298}TKPREEQY^{306}NSTYR^{310}.V	1403.5073	Gal(GlcNAc)Man$_3$(GlcNAc)$_2$Fuc	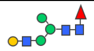
1297.0090(2+)	2593.0102	2593.0193	-4	1189.5120	R.^{302}EEQY^{306}NSTYR^{310}.V	1403.5073	Gal(GlcNAc)Man$_3$(GlcNAc)$_2$Fuc	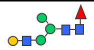
820.3527(4+)	3278.3873	3278.3952	-3	1671.8085	K.^{298}TKPREEQY^{306}NSTYR^{310}.V	1606.5867	Gal(GlcNAc)$_2$Man$_3$(GlcNAc)$_2$Fuc	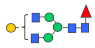
1093.4688(3+)H	3278.3908	3278.3952	-1	1671.8085	K.^{298}TKPREEQY^{306}NSTYR^{310}.V	1606.5867	Gal(GlcNAc)$_2$Man$_3$(GlcNAc)$_2$Fuc	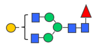
1093.4683(3+)	3278.3893	3278.3952	-2	1671.8085	K.^{298}TKPREEQY^{306}NSTYR^{310}.V	1606.5867	Gal(GlcNAc)$_2$Man$_3$(GlcNAc)$_2$Fuc	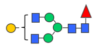
1639.7013(2+)	3278.3948	3278.3952	0	1671.8085	K.^{298}TKPREEQY^{306}NSTYR^{310}.V	1606.5867	Gal(GlcNAc)$_2$Man$_3$(GlcNAc)$_2$Fuc	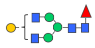
1147.4844(3+)	3440.4376	3440.4480	-3	1671.8085	K.^{298}TKPREEQY^{306}NSTYR^{310}.V	1768.6395	Gal$_2$(GlcNAc)$_2$Man$_3$(GlcNAc)$_2$Fuc	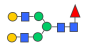
860.8652(4+)	3440.4373	3440.4480	-3	1671.8085	K.^{298}TKPREEQY^{306}NSTYR^{310}.V	1768.6395	Gal$_2$(GlcNAc)$_2$Man$_3$(GlcNAc)$_2$Fuc	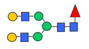
1147.4862(3+)	3440.443	3440.448	-2	1671.8085	K.^{298}TKPREEQY^{306}NSTYR^{310}.V	1768.6395	Gal$_2$(GlcNAc)$_2$Man$_3$(GlcNAc)$_2$Fuc	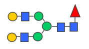

1215.1786(3+)	3643.5202	3643.5274	-2	1671.8085	K.^{298}TKPREEQY^{306}NSTYR^{310}.V	1971.7189	Gal$_2$(GlcNAc)$_3$Man$_3$(GlcNAc)$_2$Fuc	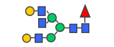
941.6364(4+)	3763.5221	3763.5333	-3	1671.8085	K.^{298}TKPREEQY^{306}NSTYR^{310}.V	2091.7248	(Neu5Gc)Gal$_3$(GlcNAc)$_2$Man$_3$(GlcNAc)$_2$	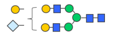
1201.5031(3+)	3602.4937	3602.5008	-2	1671.8085	K.^{298}TKPREEQY^{306}NSTYR^{310}.V	1930.6923	Gal$_3$(GlcNAc)$_2$Man$_3$(GlcNAc)$_2$Fuc	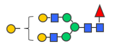
1255.5210(3+)	3764.5474	3764.5537	-2	1671.8085	K.^{298}TKPREEQY^{306}NSTYR^{310}.V	2092.7452	Gal$_4$(GlcNAc)$_2$Man$_3$(GlcNAc)$_2$Fuc	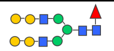
1250.1879(3+)	3748.5481	3748.5587	-3	1671.8085	K.^{298}TKPREEQY^{306}NSTYR^{310}.V	2076.7502	Gal$_3$(GlcNAc)$_2$FucMan$_3$(GlcNAc)$_2$Fuc	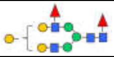
1357.8840(3+)	4071.6364	4071.644	-2	1671.8085	K.^{298}TKPREEQY^{306}NSTYR^{310}.V	2399.8355	(Neu5Gc)Gal$_4$(GlcNAc)$_2$Man$_3$(GlcNAc)$_2$ Fuc	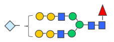
809.8409(4+)	3236.3401	3236.3482	-3	1671.8085	K.^{298}TKPREEQY^{306}NSTYR^{310}.V	1564.5397	(Neu5Gc)Gal(GlcNAc)Man$_3$(GlcNAc)$_2$	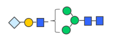
1079.4526(3+)	3236.3422	3236.3482	-2	1671.8085	K.^{298}TKPREEQY^{306}NSTYR^{310}.V	1564.5397	(Neu5Gc)Gal(GlcNAc)Man$_3$(GlcNAc)$_2$	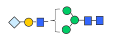
1133.4697(3+)	3398.3935	3398.4011	-2	1671.8085	K.^{298}TKPREEQY^{306}NSTYR^{310}.V	1726.5926	(Neu5Gc)Gal(GlcNAc)Man$_4$(GlcNAc)$_2$	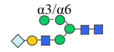
890.8674(4+)	3560.4461	3560.4539	-2	1671.8085	K.^{298}TKPREEQY^{306}NSTYR^{310}.V	1888.6454	(Neu5Gc)Gal(GlcNAc)Man$_5$(GlcNAc)$_2$	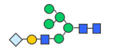
890.8681(4+)	3560.4489	3560.4539	-1	1671.8085	K.^{298}TKPREEQY^{306}NSTYR^{310}.V	1888.6454	(Neu5Gc)Gal(GlcNAc)Man$_5$(GlcNAc)$_2$	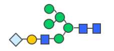
1187.4868(3+)	3560.4448	3560.4539	-3	1671.8085	K.^{298}TKPREEQY^{306}NSTYR^{310}.V	1888.6454	(Neu5Gc)Gal(GlcNAc)Man$_5$(GlcNAc)$_2$	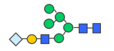
860.6105(4+)	3439.4185	3439.4276	-3	1671.8085	K.^{298}TKPREEQY^{306}NSTYR^{310}.V	1767.6191	(Neu5Gc)Gal(GlcNAc)$_2$Man$_3$(GlcNAc)$_2$	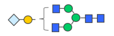
901.1225(4+)	3601.4665	3601.4804	-4	1671.8085	K.^{298}TKPREEQY^{306}NSTYR^{310}.V	1929.6719	(Neu5Gc)Gal$_2$(GlcNAc)$_2$Man$_3$(GlcNAc)$_2$	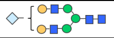
846.3555(4+)	3382.3985	3382.4062	-2	1671.8085	K.^{298}TKPREEQY^{306}NSTYR^{310}.V	1710.5977	(Neu5Gc)Gal(GlcNAc)Man$_3$(GlcNAc)$_2$<br>Fuc	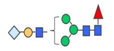
886.8693(4+)	3544.4537	3544.4590	-2	1671.8085	K.^{298}TKPREEQY^{306}NSTYR^{310}.V	1872.6505	(Neu5Gc)Gal(GlcNAc)Man$_4$(GlcNAc)$_2$<br>Fuc	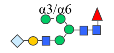
850.3542(4+)	3398.3933	3398.4011	-2	1671.8085	K.^{298}TKPREEQY^{306}NSTYR^{310}.V	1726.5926	(Neu5Gc)Gal(GlcNAc)Man$_4$(GlcNAc)$_2$	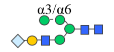
846.3556(4+)	3382.3989	3382.4062	-2	1671.8085	K.^{298}TKPREEQY^{306}NSTYR^{310}.V	1710.5977	(Neu5Gc)Gal(GlcNAc)Man$_3$(GlcNAc)$_2$<br>Fuc	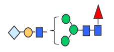
1128.1393(3+)	3382.4023	3382.4062	-1	1671.8085	K.^{298}TKPREEQY^{306}NSTYR^{310}.V	1710.5977	(Neu5Gc)Gal(GlcNAc)Man$_3$(GlcNAc)$_2$<br>Fuc	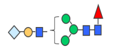
886.8693(4+)	3544.4537	3544.4590	-2	1671.8085	K.^{298}TKPREEQY^{306}NSTYR^{310}.V	1872.6505	(Neu5Gc)Gal(GlcNAc)Man$_4$(GlcNAc)$_2$<br>Fuc	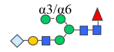
927.3812(4+)	3706.5013	3706.5118	-3	1671.8085	K.^{298}TKPREEQY^{306}NSTYR^{310}.V	2034.7033	(Neu5Gc)Gal(GlcNAc)Man$_5$(GlcNAc)$_2$Fuc	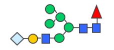
1236.1730(3+)	3706.5034	3706.5118	-2	1671.8085	K.^{298}TKPREEQY^{306}NSTYR^{310}.V	2034.7033	(Neu5Gc)Gal(GlcNAc)Man$_5$(GlcNAc)$_2$Fuc	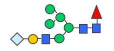
897.1250(4+)H	3585.4765	3585.4855	-3	1671.8085	K.^{298}TKPREEQY^{306}NSTYR^{310}.V	1913.6770	(Neu5Gc)Gal(GlcNAc)$_2$Man$_3$(GlcNAc)$_2$ Fuc	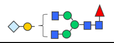
937.6401(4+)	3747.5369	3747.5383	0	1671.8085	K.^{298}TKPREEQY^{306}NSTYR^{310}.V	2075.7298	(Neu5Gc)Gal(GlcNAc)$_2$Man$_3$(GlcNAc)$_2$<br>Fuc	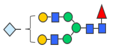
1249.8480(3+)	3747.5284	3747.5383	-3	1671.8085	K.^{298}TKPREEQY^{306}NSTYR^{310}.V	2075.7298	(Neu5Gc)Gal(GlcNAc)$_2$Man$_3$(GlcNAc)$_2$Fuc	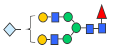
978.1528(4+)	3909.5877	3909.5912	-1	1671.8085	K.^{298}TKPREEQY^{306}NSTYR^{310}.V	2237.7827	(Neu5Gc)Gal$_3$(GlcNAc)$_2$Man$_3$(GlcNAc)$_2$Fuc	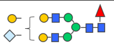
1303.8654(3+)	3909.5806	3909.5912	-3	1671.8085	K.^{298}TKPREEQY^{306}NSTYR^{310}.V	2237.7827	(Neu5Gc)Gal$_3$(GlcNAc)$_2$Man$_3$(GlcNAc)$_2$ Fuc	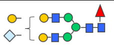
1128.1394(3+)	3382.4026	3382.4062	-1	1671.8085	K.^{298}TKPREEQY^{306}NSTYR^{310}.V	1710.5977	(Neu5Gc)Gal(GlcNAc)Man$_3$(GlcNAc)$_2$Fuc	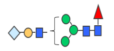
1182.1567(3+)	3544.4545	3544.459	-1	1671.8085	K.^{298}TKPREEQY^{306}NSTYR^{310}.V	1872.6505	(Neu5Gc)Gal(GlcNAc)Man$_4$(GlcNAc)$_2$Fuc	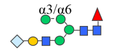
893.1271(4+)	3569.4849	3569.4906	-2	1671.8085	K.^{298}TKPREEQY^{306}NSTYR^{310}.V	1897.6821	(Neu5Ac)Gal(GlcNAc)$_2$Man$_3$(GlcNAc)$_2$ Fuc	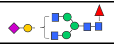
933.6397(4+)	3731.5353	3731.5434	-2	1671.8085	K.^{298}TKPREEQY^{306}NSTYR^{310}.V	2059.7349	(Neu5Ac)Gal$_2$(GlcNAc)$_2$Man$_3$(GlcNAc)$_2$ Fuc	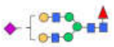
1195.8311(3+)	3585.4777	3585.4855	-2	1671.8085	K.^{298}TKPREEQY^{306}NSTYR^{310}.V	1913.6770	(Neu5Gc)Gal(GlcNAc)$_2$Man$_3$(GlcNAc)$_2$ Fuc	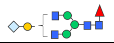
1195.8336(3+)	3585.4852	3585.4855	0	1671.8085	K.^{298}TKPREEQY^{306}NSTYR^{310}.V	1913.6770	(Neu5Gc)Gal(GlcNAc)$_2$Man$_3$(GlcNAc)$_2$ Fuc	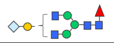
1249.8486(3+)	3747.5302	3747.5383	-2	1671.8085	K.^{298}TKPREEQY^{306}NSTYR^{310}.V	2075.7298	(Neu5Gc)Gal(GlcNAc)$_2$Man$_3$(GlcNAc)$_2$ Fuc	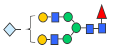

1249.8507(3+)	3747.5365	3747.5383	-1	1671.8085	K.^{298}TKPREEQY^{306}NSTYR^{310}.V	2075.7298	(Neu5Gc)Gal(GlcNAc)$_2$Man$_3$(GlcNAc)$_2$ Fuc	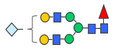
1303.8671(3+)	3909.5857	3909.5912	-1	1671.8085	K.^{298}TKPREEQY^{306}NSTYR^{310}.V	2237.7827	(Neu5Gc)Gal$_3$(GlcNAc)$_2$Man$_3$(GlcNAc)$_2$ Fuc	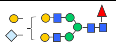
1298.5344(3+)	3893.5876	3893.5963	-2	1671.8085	K.^{298}TKPREEQY^{306}NSTYR^{310}.V	2221.7878	(Neu5Ac)Gal$_3$(GlcNAc)$_2$Man$_3$(GlcNAc)$_2$ Fuc	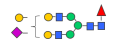
1691.7031(2+)	3382.3984	3382.4062	-2	1671.8085	K.^{298}TKPREEQY^{306}NSTYR^{310}.V	1710.5977	(Neu5Gc)Gal(GlcNAc)Man$_3$(GlcNAc)$_2$Fuc	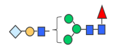
1230.8418(3+)	3690.5098	3690.5169	-2	1671.8085	K.^{298}TKPREEQY^{306}NSTYR^{310}.V	2018.7084	(Neu5Ac)Gal(GlcNAc)Man$_5$(GlcNAc)$_2$Fuc	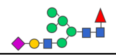
977.8963(4+)	3908.5617	3908.5708	-2	1671.8085	K.^{298}TKPREEQY^{306}NSTYR^{310}.V	2236.7623	(Neu5Gc)$_2$Gal$_2$(GlcNAc)$_2$Man$_3$(GlcNAc)$_2$	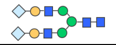
1120.1208(3+)	3358.3468	3358.352	-2	1671.8085	K.^{298}TKPREEQY^{306}NSTYR^{310}.V	1686.5435	Gal(GlcNAc)$_2$Man$_3$(GlcNAc)$_2$FucSO$_3$	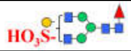
1174.1383(3+)	3520.3993	3520.4048	-2	1671.8085	K.^{298}TKPREEQY^{306}NSTYR^{310}.V	1848.5963	Gal$_2$(GlcNAc)$_2$Man$_3$(GlcNAc)$_2$FucSO$_3$	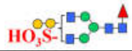
1342.5598(2+)	2684.1118	2684.1203	-3	1442.6659	K.^{300}PREEQY^{306}NSTYR^{310}.V	1241.4544	(GlcNAc)Man$_3$(GlcNAc)$_2$Fuc	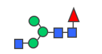
1525.1289(2+)	3049.2500	3049.2526	-1	1442.6659	K.^{300}PREEQY^{306}NSTYR^{310}.V	1606.5867	Gal(GlcNAc)$_2$Man$_3$(GlcNAc)$_2$Fuc	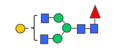
1444.1018(2+)	2887.1958	2887.1998	-1	1442.6659	K.^{300}PREEQY^{306}NSTYR^{310}.V	1444.5339	(GlcNAc)$_2$Man$_3$(GlcNAc)$_2$Fuc	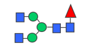
1111.1061(3+)	3331.3027	3331.3113	-3	1442.6659	K.^{300}PREEQY^{306}NSTYR^{310}.V	1888.6454	(Neu5Gc)Gal(GlcNAc)Man$_5$(GlcNAc)$_2$	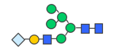
1173.4678 (3+)	3518.3878	3518.3957	-2	1442.6659	K.^{300}PREEQY^{306}NSTYR^{310}.V	2075.7298	(Neu5Gc)Gal(GlcNAc)$_2$Man$_3$(GlcNAc)$_2$ Fuc	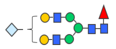
1119.4508(3+)	3356.3368	3356.3429	-2	1442.6659	K.^{300}PREEQY^{306}NSTYR^{310}.V	1913.6770	(Neu5Gc)Gal(GlcNAc)$_2$Man$_3$(GlcNAc)$_2$ Fuc	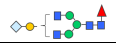
1122.4421(2+)	2243.8764	2243.8820	-3	1189.5120	R.^{302}EEQY^{306}NSTYR^{310}.V	1054.3700	Man$_4$(GlcNAc)$_2$	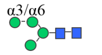
1203.4694(2+)	2405.931	2405.9349	-2	1189.5120	R.^{302}EEQY^{306}NSTYR^{310}.V	1216.4229	Man$_5$(GlcNAc)$_2$	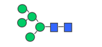
1203.4689(2+)	2405.9300	2405.9349	-2	1189.5120	R.^{302}EEQY^{306}NSTYR^{310}.V	1216.4229	Man$_5$(GlcNAc)$_2$	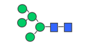
1284.4948(2+)	2567.9818	2567.9877	-2	1189.5120	R.^{302}EEQY^{306}NSTYR^{310}.V	1378.4757	Man$_6$(GlcNAc)$_2$	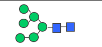
1365.5214(2+)	2730.035	2730.0405	-2	1189.5120	R.^{302}EEQY^{306}NSTYR^{310}.V	1540.5285	Man$_7$(GlcNAc)$_2$	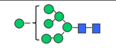
1446.5487(2+)	2892.0896	2892.0933	-1	1189.5120	R.^{302}EEQY^{306}NSTYR^{310}.V	1702.5813	Man$_8$(GlcNAc)$_2$	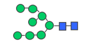
1114.4443(2+)	2227.8808	2227.8871	-3	1189.5120	R.^{302}EEQY^{306}NSTYR^{310}.V	1038.3751	Man$_3$(GlcNAc)$_2$Fuc	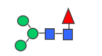
1195.4702(2+)	2389.9326	2389.9399	-3	1189.5120	R.^{302}EEQY^{306}NSTYR^{310}.V	1200.4279	Man$_4$(GlcNAc)$_2$Fuc	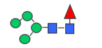
1276.4974(2+)	2551.9870	2551.9928	-2	1189.5120	R.^{302}EEQY^{306}NSTYR^{310}.V	1362.4808	Man$_5$(GlcNAc)$_2$Fuc	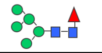
1305.0082(2+)	2609.0086	2609.0142	-2	1189.5120	R.^{302}EEQY^{306}NSTYR^{310}.V	1419.5022	(GlcNAc)Man$_5$(GlcNAc)$_2$	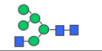
1386.0333(2+)	2771.0588	2771.0671	-3	1189.5120	R.^{302}EEQY^{306}NSTYR^{310}.V	1581.5551	Gal(GlcNAc)Man$_5$(GlcNAc)$_2$	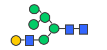
1061.9301(2+)	2122.8524	2122.8558	-2	1189.5120	R.^{302}EEQY^{306}NSTYR^{310}.V	933.3438	(GlcNAc)Man$_2$(GlcNAc)$_2$	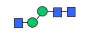
1223.9830(2+)	2446.9582	2446.9614	-1	1189.5120	R.^{302}EEQY^{306}NSTYR^{310}.V	1257.4494	(GlcNAc)Man$_4$(GlcNAc)$_2$	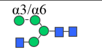
1142.9559(2+)	2284.9040	2284.9086	-2	1189.5120	R.^{302}EEQY^{306}NSTYR^{310}.V	1095.3966	(GlcNAc)Man$_3$(GlcNAc)$_2$	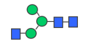
1142.9558(2+)	2284.9038	2284.9086	-2	1189.5120	R.^{302}EEQY^{306}NSTYR^{310}.V	1095.3966	(GlcNAc)Man$_3$(GlcNAc)$_2$	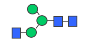
1223.9817(2+)	2446.9556	2446.9614	-3	1189.5120	R.^{302}EEQY^{306}NSTYR^{310}.V	1257.4494	(GlcNAc)Man$_4$(GlcNAc)$_2$	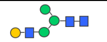
1244.4956(2+)	2487.9834	2487.9880	-2	1189.5120	R.^{302}EEQY^{306}NSTYR^{310}.V	1298.4760	(GlcNAc)$_2$Man$_3$(GlcNAc)$_2$	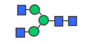
1406.5472 (2+)	2812.0866	2812.0936	-3	1189.5120	R.^{302}EEQY^{306}NSTYR^{310}.V	1622.5816	Gal$_2$(GlcNAc)$_2$Man$_3$(GlcNAc)$_2$	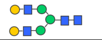
1134.9583(2+)	2268.9088	2268.9137	-2	1189.5120	R.^{302}EEQY^{306}NSTYR^{310}.V	1079.4017	(GlcNAc)Man$_2$(GlcNAc)$_2$Fuc	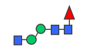
1134.9591(2+)	2268.9104	2268.9137	-2	1189.5120	R.^{302}EEQY^{306}NSTYR^{310}.V	1079.4017	(GlcNAc)Man$_2$(GlcNAc)$_2$Fuc	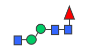

1134.9579(2+)	2268.9080	2268.9137	-3	1189.5120	R.[302]EEQY[306]NSTYR[310].V	1079.4017	(GlcNAc)Man$_2$(GlcNAc)$_2$Fuc	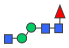
1134.9584(2+)	2268.909	2268.9137	-2	1189.5120	R.[302]EEQY[306]NSTYR[310].V	1079.4017	(GlcNAc)Man$_2$(GlcNAc)$_2$Fuc	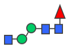
1134.9581(2+)	2268.9084	2268.9137	-2	1189.5120	R.[302]EEQY[306]NSTYR[310].V	1079.4017	(GlcNAc)Man$_2$(GlcNAc)$_2$Fuc	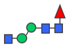
1215.9851(2+)	2430.9624	2430.9665	-2	1189.5120	R.[302]EEQY[306]NSTYR[310].V	1241.4545	(GlcNAc)Man$_3$(GlcNAc)$_2$Fuc	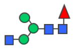
1215.9841(2+)	2430.9604	2430.9665	-3	1189.5120	R.[302]EEQY[306]NSTYR[310].V	1241.4545	(GlcNAc)Man$_3$(GlcNAc)$_2$Fuc	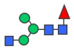
1215.9849(2+)	2430.962	2430.9665	-2	1189.5120	R.[302]EEQY[306]NSTYR[310].V	1241.4545	(GlcNAc)Man$_3$(GlcNAc)$_2$Fuc	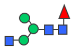
878.6852(3+)	2634.04	2634.0459	-2	1189.5120	R.[302]EEQY[306]NSTYR[310].V	1444.5339	(GlcNAc)$_2$Man$_3$(GlcNAc)$_2$Fuc	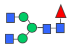
1317.5247(2+)	2634.0416	2634.0459	-2	1189.5120	R.[302]EEQY[306]NSTYR[310].V	1444.5339	(GlcNAc)$_2$Man$_3$(GlcNAc)$_2$Fuc	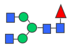
1317.5236(2+)	2634.0394	2634.0459	-3	1189.5120	R.[302]EEQY[306]NSTYR[310].V	1444.5339	(GlcNAc)$_2$Man$_3$(GlcNAc)$_2$Fuc	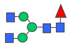
1317.5245(2+)	2634.0412	2634.0459	-2	1189.5120	R.[302]EEQY[306]NSTYR[310].V	1444.5339	(GlcNAc)$_2$Man$_3$(GlcNAc)$_2$Fuc	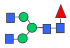
1317.5237(2+)	2634.0396	2634.0459	-3	1189.5120	R.[302]EEQY[306]NSTYR[310].V	1444.5339	(GlcNAc)$_2$Man$_3$(GlcNAc)$_2$Fuc	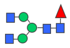
1317.5256(2+)	2634.0434	2634.0459	-1	1189.5120	R.[302]EEQY[306]NSTYR[310].V	1444.5339	(GlcNAc)$_2$Man$_3$(GlcNAc)$_2$Fuc	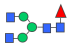
1297.0111(2+)	2593.0144	2593.0193	-2	1189.5120	R.[302]EEQY[306]NSTYR[310].V	1403.5073	Gal(GlcNAc)Man$_3$(GlcNAc)$_2$Fuc	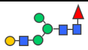
1297.0107(2+)	2593.0136	2593.0193	-2	1189.5120	R.[302]EEQY[306]NSTYR[310].V	1403.5073	Gal(GlcNAc)Man$_3$(GlcNAc)$_2$Fuc	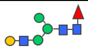
1297.0098(2+)	2593.0118	2593.0193	-3	1189.5120	R.[302]EEQY[306]NSTYR[310].V	1403.5073	Gal(GlcNAc)Man$_3$(GlcNAc)$_2$Fuc	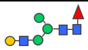
1378.0377(2+)	2755.0676	2755.0721	-2	1189.5120	R.[302]EEQY[306]NSTYR[310].V	1565.5601	(GlcNAc)Man$_5$(GlcNAc)$_2$Fuc	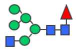
1378.0371(2+)	2755.0664	2755.0721	-2	1189.5120	R.[302]EEQY[306]NSTYR[310].V	1565.5601	(GlcNAc)Man$_5$(GlcNAc)$_2$Fuc	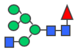
1459.0643(2+)	2917.1208	2917.1249	-1	1189.5120	R.[302]EEQY[306]NSTYR[310].V	1727.6129	Gal(GlcNAc)Man$_5$(GlcNAc)$_2$Fuc	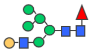
1297.0105(2+)	2593.0132	2593.0193	-2	1189.5120	R.[302]EEQY[306]NSTYR[310].V	1403.5073	Gal(GlcNAc)Man$_3$(GlcNAc)$_2$Fuc	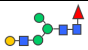
1215.9858(2+)	2430.9638	2430.9664	-1	1189.5120	R.[302]EEQY[306]NSTYR[310].V	1241.4544	(GlcNAc)Man$_3$(GlcNAc)$_2$Fuc	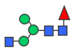
932.7038(3+)	2796.0958	2796.0987	-1	1189.5120	R.[302]EEQY[306]NSTYR[310].V	1606.5867	Gal(GlcNAc)$_2$Man$_3$(GlcNAc)$_2$Fuc	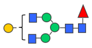
1398.5514(2+)	2796.0950	2796.0987	-1	1189.5120	R.[302]EEQY[306]NSTYR[310].V	1606.5867	Gal(GlcNAc)$_2$Man$_3$(GlcNAc)$_2$Fuc	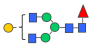
986.7205(3+)	2958.1459	2958.1515	-2	1189.5120	R.[302]EEQY[306]NSTYR[310].V	1768.6395	Gal$_2$(GlcNAc)$_2$Man$_3$(GlcNAc)$_2$Fuc	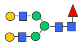
1479.5776(2+)	2958.1474	2958.1515	-1	1189.5120	R.[302]EEQY[306]NSTYR[310].V	1768.6395	Gal$_2$(GlcNAc)$_2$Man$_3$(GlcNAc)$_2$Fuc	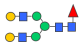
1040.7382(3+)	3120.199	3120.2043	-2	1189.5120	R.[302]EEQY[306]NSTYR[310].V	1930.6923	Gal$_3$(GlcNAc)$_2$Man$_3$(GlcNAc)$_2$Fuc	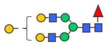
1560.6041(2+)	3120.2004	3120.2043	-1	1189.5120	R.[302]EEQY[306]NSTYR[310].V	1930.6923	Gal$_3$(GlcNAc)$_2$Man$_3$(GlcNAc)$_2$Fuc	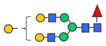
1094.7567(3+)	3282.2545	3282.2572	-1	1189.5120	R.[302]EEQY[306]NSTYR[310].V	2092.7452	Gal$_4$(GlcNAc)$_2$Man$_3$(GlcNAc)$_2$Fuc	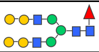
1641.6300(2+)	3282.2522	3282.2572	-2	1189.5120	R.[302]EEQY[306]NSTYR[310].V	2092.7452	Gal$_4$(GlcNAc)$_2$Man$_3$(GlcNAc)$_2$Fuc	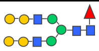
1143.1013(3+)	3427.2883	3427.2947	-2	1189.5120	R.[302]EEQY[306]NSTYR[310].V	2237.7827	(Neu5Gc)Gal$_3$(GlcNAc)$_2$Man$_3$(GlcNAc)$_2$Fuc	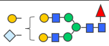
918.6874(3+)	2754.0466	2754.0517	-2	1189.5120	R.[302]EEQY[306]NSTYR[310].V	1564.5397	(Neu5Gc)Gal(GlcNAc)Man$_3$(GlcNAc)$_2$	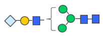
1377.5280(2+)	2754.0482	2754.0517	-1	1189.5120	R.[302]EEQY[306]NSTYR[310].V	1564.5397	(Neu5Gc)Gal(GlcNAc)Man$_3$(GlcNAc)$_2$	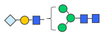
972.7052(3+)	2916.1	2916.1046	-2	1189.5120	R.[302]EEQY[306]NSTYR[310].V	1726.5926	(Neu5Gc)Gal(GlcNAc)Man$_4$(GlcNAc)$_2$	α3/α6 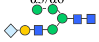

1458.5533(2+)	2916.0988	2916.1046	-2	1189.5120	R.^{302}EEQY^{306}NSTYR^{310}.V	1726.5926	(Neu5Gc)Gal(GlcNAc)Man$_4$(GlcNAc)$_2$	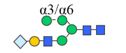
1026.7227(3+)	3078.1525	3078.1574	-2	1189.5120	R.^{302}EEQY^{306}NSTYR^{310}.V	1888.6454	(Neu5Gc)Gal(GlcNAc)Man$_5$(GlcNAc)$_2$	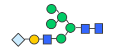
1539.5798(2+)	3078.1518	3078.1574	-2	1189.5120	R.^{302}EEQY^{306}NSTYR^{310}.V	1888.6454	(Neu5Gc)Gal(GlcNAc)Man$_5$(GlcNAc)$_2$	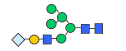
986.3799(3+)	2957.1241	2957.1311	-3	1189.5120	R.^{302}EEQY^{306}NSTYR^{310}.V	1767.6191	(Neu5Gc)Gal(GlcNAc)$_2$Man$_3$(GlcNAc)$_2$	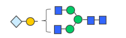
1479.0691(2+)	2957.1304	2957.1311	0	1189.5120	R.^{302}EEQY^{306}NSTYR^{310}.V	1767.6191	(Neu5Gc)Gal(GlcNAc)$_2$Man$_3$(GlcNAc)$_2$	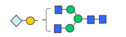
1142.7606(3+)	3426.2662	3426.2743	-3	1189.5120	R.^{302}EEQY^{306}NSTYR^{310}.V	2236.7623	(Neu5Gc)$_2$Gal$_2$(GlcNAc)$_2$Man$_3$(GlcNAc)$_2$	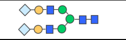
1450.5555(2+)	2900.1032	2900.1097	-2	1189.5120	R.^{302}EEQY^{306}NSTYR^{310}.V	1710.5977	(Neu5Gc)Gal(GlcNAc)Man$_3$(GlcNAc)$_2$Fuc	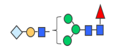
967.3735(3+)	2900.1049	2900.1097	-2	1189.5120	R.^{302}EEQY^{306}NSTYR^{310}.V	1710.5977	(Neu5Gc)Gal(GlcNAc)Man$_3$(GlcNAc)$_2$Fuc	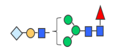
1021.3908(3+)	3062.1568	3062.1625	-2	1189.5120	R.^{302}EEQY^{306}NSTYR^{310}.V	1872.6505	(Neu5Gc)Gal(GlcNAc)Man$_4$(GlcNAc)$_2$Fuc	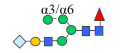
1075.4083(3+)	3224.2093	3224.2153	-2	1189.5120	R.^{302}EEQY^{306}NSTYR^{310}.V	2034.7033	(Neu5Gc)Gal(GlcNAc)Man$_5$(GlcNAc)$_2$Fuc	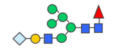
1089.0841(3+)	3265.2367	3265.2418	-2	1189.5120	R.^{302}EEQY^{306}NSTYR^{310}.V	2075.7298	(Neu5Gc)Gal(GlcNAc)$_2$Man$_3$(GlcNAc)$_2$Fuc	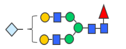
1552.0948(2+)	3103.1818	3103.1890	-2	1189.5120	R.^{302}EEQY^{306}NSTYR^{310}.V	1913.6770	(Neu5Gc)Gal(GlcNAc)$_2$Man$_3$(GlcNAc)$_2$Fuc	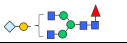
1612.6088(2+)	3224.2098	3224.2153	-2	1189.5120	R.^{302}EEQY^{306}NSTYR^{310}.V	2034.7033	(Neu5Gc)Gal(GlcNAc)Man$_5$(GlcNAc)$_2$Fuc	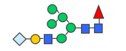
1035.0670(3+)	3103.1854	3103.1890	-1	1189.5120	R.^{302}EEQY^{306}NSTYR^{310}.V	1913.6770	(Neu5Gc)Gal(GlcNAc)$_2$Man$_3$(GlcNAc)$_2$ Fuc	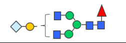
1369.5293(2+)	2738.0508	2738.0568	-2	1189.5120	R.^{302}EEQY^{306}NSTYR^{310}.V	1548.5448	(Neu5Ac)Gal(GlcNAc)Man$_3$(GlcNAc)$_2$	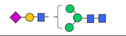
1531.5823(2+)	3062.1568	3062.1625	-2	1189.5120	R.^{302}EEQY^{306}NSTYR^{310}.V	1872.6505	(Neu5Gc)Gal(GlcNAc)Man$_4$(GlcNAc)$_2$Fuc	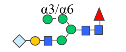
1633.1216(2+)	3265.2354	3265.2418	-2	1189.5120	R.^{302}EEQY^{306}NSTYR^{310}.V	2075.7298	(Neu5Gc)Gal(GlcNAc)$_2$Man$_3$(GlcNAc)$_2$Fuc	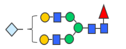
1029.7361(3+)	3087.1927	3087.1941	0	1189.5120	R.^{302}EEQY^{306}NSTYR^{310}.V	1897.6821	(Neu5Ac)Gal(GlcNAc)$_2$Man$_3$(GlcNAc)$_2$Fuc	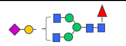
1083.7517(3+)	3249.2395	3249.2469	-2	1189.5120	R.^{302}EEQY^{306}NSTYR^{310}.V	2059.7349	(Neu5Ac)Gal$_2$(GlcNAc)$_2$Man$_3$(GlcNAc)$_2$Fuc	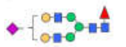
1625.1248(2+)	3249.2418	3249.2469	-2	1189.5120	R.^{302}EEQY^{306}NSTYR^{310}.V	2059.7349	(Neu5Ac)Gal$_2$(GlcNAc)$_2$Man$_3$(GlcNAc)$_2$Fuc	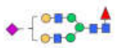
1089.4242(3+)	3266.2570	3266.2622	-2	1189.5120	R.^{302}EEQY^{306}NSTYR^{310}.V	2076.7502	Gal$_3$(GlcNAc)$_2$FucMan$_3$(GlcNAc)$_2$Fuc	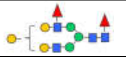
1137.7693(3+)	3411.2923	3411.2997	-2	1189.5120	R.^{302}EEQY^{306}NSTYR^{310}.V	2221.7877	(Neu5Gc)Gal$_2$(GlcNAc)$_2$FucMan$_3$(GlcNAc)$_2$Fuc	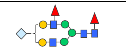
1143.1009(3+)	3427.2871	3427.2947	-2	1189.5120	R.^{302}EEQY^{306}NSTYR^{310}.V	2237.7827	(Neu5Gc)Gal$_3$(GlcNAc)$_2$Man$_3$(GlcNAc)$_2$Fuc	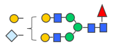
1191.4486(3+)	3572.3302	3572.3322	-1	1189.5120	R.^{302}EEQY^{306}NSTYR^{310}.V	2382.8202	(Neu5Gc)$_2$Gal$_2$(GlcNAc)$_2$Man$_3$(GlcNAc)$_2$Fuc	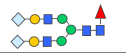

* The N-glycopeptides are determined by reversed-phase (RP) LC MS/MS of a tryptic Golimumab digest, and identified by database search using Byonic software.

§ Missed cleavages of Fc peptides at residues TKPREEQYNSTYR were also observed due to a low hydrolysis rate caused by the suppression of trypsin activity resulting from the co-existence of acidic residues (*i.e.* glutamic acid) and proline near the cleavage site (Rodriguez, J.; Gupta, N.; Smith, R. D.; Pevzner, P. A. *J Proteome Res.* **2008**, 7, 300-305).

\# Possible structures of N-glycans are predicted based on the initial database search against the in-house mAb glycan library by the accurate mass, glycan composition and the structures derived from the glycan biosynthesis pathway. The isomeric glycan structures are not distinguishable at the glycopeptide level.

Table S6 Peak areas of α-Gal containing N-glycopeptides

mAb	m/z 1040.7400	Mean	SD	*m/z* 1094.7580	Mean	SD	*m/z* 1137.7710	Mean	SD	*m/z* 1143.1030	Mean	SD
Bevacizumab	-	-	-	-	-	-	-	-	-	--	-	-
Trastuzumab	5198386<br>6032092<br>5674179	5634885.7	341491.2	-	-	-	-	-	-	-	-	-
Adalimum	-	-	-	-	-	-	-	-	-	-	-	-
Rituximab	18478165<br>15754868<br>17742376	17325136.3	1150261.8	1833841<br>1939178<br>1808459	1860492.7	56595.6	3816421<br>5702516<br>4043126	4520687.<br>7	840788.3	-	-	-
Palivizumab	568337672<br>580522678<br>558321399	569060583	9078037.5	292465470<br>304812337<br>281449678	292909161.7	9542924.3	8379155<br>9388361<br>10402885	9390133.<br>7	826185.3	139759251<br>158643375<br>139534748	145979124.7	8955446.3
Infliximab	31725282<br>30465667<br>30050349	30747099.3	712157.9	8288317<br>8920582<br>8061885	8423594.7	363377.8	-	-	-	14499741<br>15054268<br>14580428	14711479	244616.5
Golimumab	523902755<br>625515525<br>465425920	538281400	66142419.5	376471943<br>458883462<br>404861744	413405716.3	34182492.7	20222120<br>22192761<br>18877792	20430891	1361358.1	749072896<br>864190354<br>710488281	774583843.7	65290061.3
Cetuximab	44039264<br>52602474<br>45515332	47385690	3737719.5	17845577<br>16047353<br>17123253	17005394.3	738837.1	176853<br>175806<br>169802	174153.7	3106.6	3844775<br>4084271<br>3723868	3884304.7	149765.4

The peak areas of the α-Gal containing N-glycopeptides are determined by LC MS/MS measurements of tryptic mAbs in triplets.

Table S7. Identified glycan isomers of golimumab by PGC LC MS/MS analyses of PNGase F released glycans

Obs. *m/z* (Charge)	RT* (min)	Obs. $[MH]^+$	Calc. $[MH]^+$	Errors (ppm)	Composition	Glycan Structure*
861.3025(2+)	22.83	1721.5972	1721.5997	-2	Man$_8$(GlcNAc)$_2$	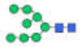 β-anomer
861.3028(2+)	24.86	1721.5978	1721.5997	-1	Man$_8$(GlcNAc)$_2$	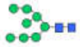 α-anomer
780.2767(2+)	22.87	1559.5456	1559.5469	-1	Man$_7$(GlcNAc)$_2$	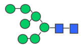 β-anomer
780.2760(2+)	24.90	1559.5442	1559.5469	-2	Man$_7$(GlcNAc)$_2$	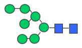 α-anomer
699.2502(2+)	22.92	1397.4926	1397.4941	-1	Man$_6$(GlcNAc)$_2$	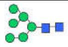 β-anomer
699.2506(2+)	24.82	1397.4934	1397.4941	-1	Man$_6$(GlcNAc)$_2$	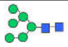 α-anomer
1235.4405(1+)	27.27	1235.4405	1235.4413	-1	Man$_5$(GlcNAc)$_2$	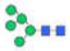 β-anomer
1235.4402(1+)	29.16	1235.4402	1235.4413	-1	Man$_5$(GlcNAc)$_2$	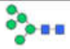 α-anomer
719.7631(2+)	22.68	1438.5184	1438.5206	-2	Gal(GlcNAc)Man$_4$(GlcNAc)$_2$	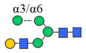 β-anomer
719.7630(2+)	23.37	1438.5182	1438.5206	-2	(GlcNAc)Man$_5$(GlcNAc)$_2$	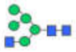 β-anomer
719.7638(2+)	25.02	1438.5198	1438.5206	-1	Gal(GlcNAc)Man$_4$(GlcNAc)$_2$	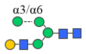 α-anomer
719.7633(2+)	25.99	1438.5188	1438.5206	-1	(GlcNAc)Man$_5$(GlcNAc)$_2$	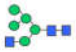 α-anomer
800.7894(2+)	24.44	1600.5710	1600.5735	-2	Gal(GlcNAc)Man$_5$(GlcNAc)$_2$	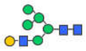 β-anomer
800.7896(2+)	27.06	1600.5714	1600.5735	-1	Gal(GlcNAc)Man$_5$(GlcNAc)$_2$	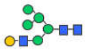 α-anomer
1114.4132(1+)	22.21	1114.4132	1114.4150	-2	(GlcNAc)Man$_3$(GlcNAc)$_2$	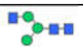 β-anomer
1114.4135(1+)	22.48	1114.4135	1114.4150	-1	(GlcNAc)Man$_3$(GlcNAc)$_2$	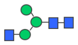 β-anomer
1114.4137(1+)	24.86	1114.4137	1114.4150	-1	(GlcNAc)Man$_3$(GlcNAc)$_2$	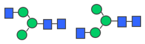 α-anomer
659.2504(2+)	22.48	1317.4930	1317.4944	-1	(GlcNAc)$_2$Man$_3$(GlcNAc)$_2$	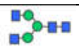 β-anomer
659.2503(2+)	24.90	1317.4928	1317.4944	-1	(GlcNAc)$_2$Man$_3$(GlcNAc)$_2$	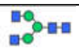 α-anomer
740.2770(2+)	23.55	1479.5462	1479.5472	-1	Gal(GlcNAc)$_2$Man$_3$(GlcNAc)$_2$	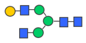 β-anomer
740.2763(2+)	23.82	1479.5468	1479.5472	-1	Gal(GlcNAc)$_2$Man$_3$(GlcNAc)$_2$	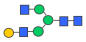 β-anomer
740.2762(2+)	26.10	1479.5446	1479.5472	-2	Gal(GlcNAc)$_2$Man$_3$(GlcNAc)$_2$	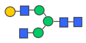 α-anomer
740.2762(2+)	26.40	1479.5446	1479.5472	-2	Gal(GlcNAc)$_2$Man$_3$(GlcNAc)$_2$	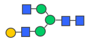 α-anomer
732.2788(2+)	25.26	1463.5498	1463.5523	-2	(GlcNAc)$_2$Man$_3$(GlcNAc)$_2$Fuc	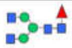 β-anomer
732.2787(2+)	28.10	1463.5496	1463.5523	-2	(GlcNAc)$_2$Man$_3$(GlcNAc)$_2$Fuc	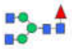 α-anomer

813.3055(2+)	26.40	1625.6032	1625.6051	-1	Gal(GlcNAc)$_2$Man$_3$(GlcNAc)$_2$Fuc	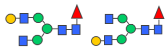 β-anomer
813.3051(2+)	29.12	1625.6024	1625.6051	-2	Gal(GlcNAc)$_2$Man$_3$(GlcNAc)$_2$Fuc	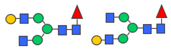 α-anomer
894.3310(2+)	27.60	1787.6542	1787.6579	-2	Gal$_2$(GlcNAc)$_2$Man$_3$(GlcNAc)$_2$Fuc	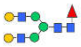 β-anomer
894.3312(2+)	28.70	1787.6546	1787.6579	-2	Gal$_2$(GlcNAc)$_2$Man$_3$(GlcNAc)$_2$Fuc	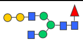 β-anomer
894.3314(2+)	30.34	1787.6550	1787.6579	-2	Gal$_2$(GlcNAc)$_2$Man$_3$(GlcNAc)$_2$Fuc	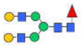 α-anomer
894.3312(2+)	31.32	1787.6546	1787.6579	-2	Gal$_2$(GlcNAc)$_2$Man$_3$(GlcNAc)$_2$Fuc	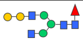 α-anomer
894.3313(2+)	32.04	1787.6548	1787.6579	-2	Gal$_2$(GlcNAc)$_2$Man$_3$(GlcNAc)$_2$Fuc	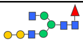 α-anomer
1056.3840(2+)	31.95	2111.7602	2111.7636	-2	Gal$_4$(GlcNAc)$_2$Man$_3$(GlcNAc)$_2$Fuc	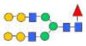 β-anomer
1056.3842(2+)	34.39	2111.7606	2111.7636	-2	Gal$_4$(GlcNAc)$_2$Man$_3$(GlcNAc)$_2$Fuc	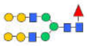 α-anomer
792.7922(2+)	25.21	1584.5766	1584.5785	-1	Gal(GlcNAc)Man$_4$(GlcNAc)$_2$Fuc	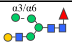 β-anomer
792.7926(2+)	26.20	1584.5774	1584.5785	-1	(GlcNAc)Man$_5$(GlcNAc)$_2$Fuc	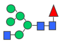 β-anomer
792.7920(2+)	27.86	1584.5762	1584.5785	-2	Gal(GlcNAc)Man$_4$(GlcNAc)$_2$Fuc	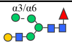 α-anomer
792.7921(2+)	28.92	1584.5764	1584.5785	-1	(GlcNAc)Man$_5$(GlcNAc)$_2$Fuc	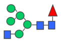 α-anomer
792.7918(2+)	32.68	1584.5758	1584.5785	-2	Gal$_2$(GlcNAc)Man$_3$(GlcNAc)$_2$Fuc	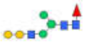 α-anomer
792.7923(2+)	37.44	1584.5768	1584.5785	-1	Gal$_2$(GlcNAc)Man$_3$(GlcNAc)$_2$Fuc	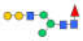 α-anomer
893.8208(2+)	35.07	1786.6338	1786.6375	-2	(Neu5Gc)Gal(GlcNAc)$_2$Man$_3$(GlcNAc)$_2$	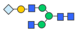 β-anomer
893.8210(2+)	35.56	1786.6342	1786.6375	-2	(Neu5Gc)Gal(GlcNAc)$_2$Man$_3$(GlcNAc)$_2$	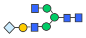 β-anomer
893.8207(2+)	38.71	1786.6336	1786.6375	-2	(Neu5Gc)Gal(GlcNAc)$_2$Man$_3$(GlcNAc)$_2$	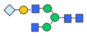 α-anomer
893.8215(2+)	39.40	1786.6352	1786.6375	-1	(Neu5Gc)Gal(GlcNAc)$_2$Man$_3$(GlcNAc)$_2$	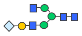 α-anomer
954.3341(2+)	36.52	1907.6604	1907.6638	-2	(Neu5Gc)Gal(GlcNAc)Man$_5$(GlcNAc)$_2$	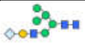 β-anomer
954.3340(2+)	40.03	1907.6602	1907.6638	-2	(Neu5Gc)Gal(GlcNAc)Man$_5$(GlcNAc)$_2$	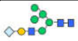 α-anomer
946.3366(2+)	37.52	1891.6654	1891.6689	-2	(Neu5Gc)Gal(GlcNAc)Man$_4$(GlcNAc)$_2$Fuc	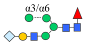 β-anomer
946.3368(2+)	40.92	1891.6658	1891.6689	-2	(Neu5Gc)Gal(GlcNAc)Man$_4$(GlcNAc)$_2$ Fuc	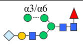 α-anomer
966.8497(2+)	38.67	1932.6916	1932.6954	-2	(Neu5Gc)Gal(GlcNAc)$_2$Man$_3$(GlcNAc)$_2$Fuc	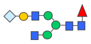 β-anomer
966.8499(2+)	39.01	1932.6920	1932.6954	-2	(Neu5Gc)Gal(GlcNAc)$_2$Man$_3$(GlcNAc)$_2$Fuc	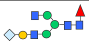 β-anomer
966.8499(2+)	41.70	1932.6920	1932.6954	-2	(Neu5Gc)Gal(GlcNAc)$_2$Man$_3$(GlcNAc)$_2$Fuc	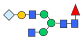 α-anomer
966.8500(2+)	42.27	1932.6922	1932.6954	-2	(Neu5Gc)Gal(GlcNAc)$_2$Man$_3$(GlcNAc)$_2$Fuc	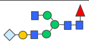 α-anomer
1027.3628(2+)	39.31	2053.7178	2053.7217	-2	(Neu5Gc)Gal(GlcNAc)Man$_5$(GlcNAc)$_2$Fuc	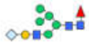 β-anomer
1027.3629(2+)	42.73	2053.7180	2053.7217	-2	(Neu5Gc)Gal(GlcNAc)Man$_5$(GlcNAc)$_2$Fuc	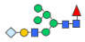 α-anomer

1047.8765(2+)	39.63	2094.7452	2094.7462	-1	(Neu5Gc)Gal$_2$(GlcNAc)$_2$Man$_3$(GlcNAc)$_2$Fuc	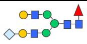 β-anomer
1047.8759(2+)	42.23	2094.7440	2094.7462	-1	(Neu5Gc)Gal$_2$(GlcNAc)$_2$Man$_3$(GlcNAc)$_2$Fuc	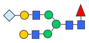 β-anomer
1047.8765(2+)	43.16	2094.7452	2094.7482	-2	(Neu5Gc)Gal$_2$(GlcNAc)$_2$Man$_3$(GlcNAc)$_2$Fuc	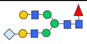 α-anomer
698.9199(3+)	43.16	2094.7441	2094.7482	-2	(Neu5Gc)Gal$_2$(GlcNAc)$_2$Man$_3$(GlcNAc)$_2$Fuc	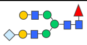 α-anomer
865.3102(2+)	40.12	1729.6126	1729.6161	-2	(Neu5Gc)Gal(GlcNAc)Man$_3$(GlcNAc)$_2$Fuc	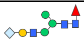 β-anomer
865.3102(2+)	44.89	1729.6126	1729.6161	-2	(Neu5Gc)Gal(GlcNAc)Man$_3$(GlcNAc)$_2$Fuc	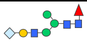 α-anomer
1128.9023(2+)	41.70	2256.7968	2256.8011	-2	(Neu5Gc)Gal$_3$(GlcNAc)$_2$Man$_3$(GlcNAc)$_2$Fuc	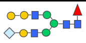 β-anomer
1128.9027(2+)	44.94	2256.7976	2256.8011	-2	(Neu5Gc)Gal$_3$(GlcNAc)$_2$Man$_3$(GlcNAc)$_2$Fuc	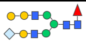 α-anomer
752.9376(3+)	44.94	2256.7972	2256.8011	-2	(Neu5Gc)Gal$_3$(GlcNAc)$_2$Man$_3$(GlcNAc)$_2$Fuc	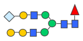 α-anomer
1120.9045(2+)	44.08	2240.8012	2240.8062	-2	(Neu5Gc)Gal$_2$(GlcNAc)$_2$Man$_3$(GlcNAc)$_2$Fuc$_2$	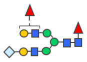 β-anomer
1120.9052(2+)	45.47	2240.8026	2240.8062	-2	(Neu5Ac)Gal$_3$(GlcNAc)$_2$Man$_3$(GlcNAc)$_2$Fuc	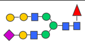 α-anomer
1120.9052(2+)	45.47	2240.8026	2240.8062	-2	(Neu5Gc)Gal$_2$(GlcNAc)$_2$Man$_3$(GlcNAc)$_2$Fuc$_2$	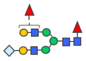 α-anomer

* Structural isomers of glycans are identified by MS/MS analyses, and the LC retention times are listed according to the chromatographic peaks of the extracted ion chromatograms (EICs).

Table S8. Identified glycan isomers of cetuximab by PGC LC MS/MS analyses of the PNGase F released glycans

**Obs. *m/z* (charge)**	**RT* (min)**	**Obs. [MH]+**	**Calc. [MH]+**	**Errors (ppm)**	**Composition**	**Glycan Structure***
780.2772(2+)	24.92	1559.5466	1559.5469	0	$Man_7(GlcNAc)_2$	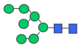 α-anomer
699.2501(2+)	23.79	1397.4924	1397.4941	-1	$Man_6(GlcNAc)_2$	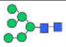 β-anomer
699.2516(2+)	24.92	1397.4954	1397.4941	1	$Man_6(GlcNAc)_2$	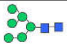 α-anomer
1235.4413(1+)	27.30	1235.4413	1235.4413	0	$Man_5(GlcNAc)_2$	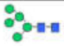 β-anomer
1235.4400(1+)	31.10	1235.4400	1235.4413	-1	$Man_5(GlcNAc)_2$	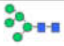 α-anomer
719.7629(2+)	22.61	1438.5180	1438.5206	-2	$Gal(GlcNAc)Man_4(GlcNAc)_2$	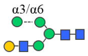 β-anomer
719.7627(2+)	23.39	1438.5176	1438.5206	-2	$(GlcNAc)Man_5(GlcNAc)_2$	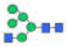 β-anomer
719.7629(2+)	25.22	1438.5180	1438.5206	-2	$Gal(GlcNAc)Man_4(GlcNAc)_2$	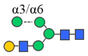 α-anomer
719.7630(2+)	26.03	1438.5182	1438.5206	-2	$(GlcNAc)Man_5(GlcNAc)_2$	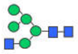 α-anomer
800.7891(2+)	24.47	1600.5704	1600.5735	-2	$Gal(GlcNAc)Man_5(GlcNAc)_2$	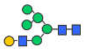 β-anomer
800.7893(2+)	27.05	1600.5708	1600.5735	-2	$Gal(GlcNAc)Man_5(GlcNAc)_2$	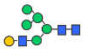 α-anomer
1114.4126(1+)	22.16	1114.4126	1114.4150	-2	$(GlcNAc)Man_3(GlcNAc)_2$	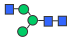 β-anomer
1114.4130(1+)	22.39	1114.4130	1114.4150	-2	$(GlcNAc)Man_3(GlcNAc)_2$	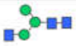 β-anomer
1114.4135(1+)	24.88	1114.4135	1114.4150	-1	$(GlcNAc)Man_3(GlcNAc)_2$	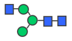 α-anomer
1114.4131(1+)	25.34	1114.4131	1114.4150	-2	$(GlcNAc)Man_3(GlcNAc)_2$	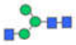 α-anomer
659.2498(2+)	22.39	1317.4918	1317.4944	-2	$(GlcNAc)_2Man_3(GlcNAc)_2$	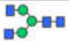 β-anomer
659.2500(2+)	24.92	1317.4922	1317.4944	-2	$(GlcNAc)_2Man_3(GlcNAc)_2$	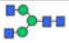 α-anomer
1317.4927(1+)	24.92	1317.4927	1317.4944	-1	$(GlcNAc)_2Man_3(GlcNAc)_2$	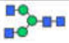 α-anomer
740.2762(2+)	23.44	1479.5446	1479.5472	-2	$Gal(GlcNAc)_2Man_3(GlcNAc)_2$	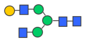 β-anomer
740.2763(2+)	23.79	1479.5448	1479.5472	-2	$Gal(GlcNAc)_2Man_3(GlcNAc)_2$	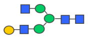 α-anomer
740.2765(2+)	26.03	1479.5452	1479.5472	-1	$Gal(GlcNAc)_2Man_3(GlcNAc)_2$	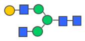 β-anomer
740.2767(2+)	26.41	1479.5456	1479.5472	-1	$Gal(GlcNAc)_2Man_3(GlcNAc)_2$	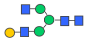 α-anomer
719.7628(2+)	22.51	1438.5178	1438.5206	-2	$Gal(GlcNAc)Man_4(GlcNAc)_2$	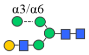 β-anomer
719.7629(2+)	23.34	1438.5180	1438.5206	-2	$(GlcNAc)Man_5(GlcNAc)_2$	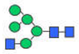 β-anomer
719.7629(2+)	25.09	1438.5180	1438.5206	-2	$Gal(GlcNAc)Man_4(GlcNAc)_2$	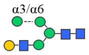 α-anomer
719.7630(2+)	26.03	1438.5182	1438.5206	-2	$(GlcNAc)Man_5(GlcNAc)_2$	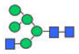 α-anomer

732.2788(2+)	25.30	1463.5498	1463.5523	-2	(GlcNAc)$_2$Man$_3$(GlcNAc)$_2$Fuc	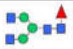 β-anomer
732.2786(2+)	28.00	1463.5494	1463.5523	-2	(GlcNAc)$_2$Man$_3$(GlcNAc)$_2$Fuc	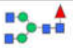 α-anomer
813.3054(2+)	26.37	1625.6030	1625.6051	-1	Gal(GlcNAc)$_2$Man$_3$(GlcNAc)$_2$Fuc	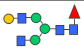 β-anomer
813.3053(2+)	26.55	1625.6028	1625.6051	-1	Gal(GlcNAc)$_2$Man$_3$(GlcNAc)$_2$Fuc	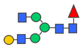 β-anomer
813.3053(2+)	29.13	1625.6028	1625.6051	-1	Gal(GlcNAc)$_2$Man$_3$(GlcNAc)$_2$Fuc	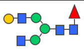 α-anomer
813.3054(2+)	29.23	1625.6030	1625.6051	-1	Gal(GlcNAc)$_2$Man$_3$(GlcNAc)$_2$Fuc	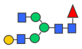 α-anomer
873.8181(2+)	27.05	1746.6284	1746.6313	-2	Gal(GlcNAc)Man$_5$(GlcNAc)$_2$Fuc	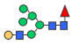 β-anomer
873.8182(2+)	27.64	1746.6286	1746.6313	-2	Gal$_2$(GlcNAc)Man$_4$(GlcNAc)$_2$Fuc	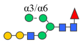 β-anomer
873.8180(2+)	29.84	1746.6282	1746.6313	-2	Gal(GlcNAc)Man$_5$(GlcNAc)$_2$Fuc	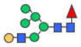 α-anomer
873.8182(2+)	30.37	1746.6286	1746.6313	-2	Gal$_2$(GlcNAc)Man$_4$(GlcNAc)$_2$Fuc	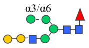 α-anomer
894.3314(2+)	27.59	1787.6550	1787.6579	-2	Gal$_2$(GlcNAc)$_2$Man$_3$(GlcNAc)$_{24}$Fuc	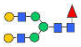 β-anomer
894.3317(2+)	28.59	1787.6556	1787.6579	-1	Gal$_2$(GlcNAc)$_2$Man$_3$(GlcNAc)$_2$Fuc	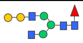 β-anomer
894.3316(2+)	29.13	1787.6554	1787.6579	-1	Gal$_2$(GlcNAc)$_2$Man$_3$(GlcNAc)$_2$Fuc	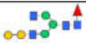 α-anomer
894.3311(2+)	30.31	1787.6544	1787.6579	-2	Gal$_2$(GlcNAc)$_2$Man$_3$(GlcNAc)$_2$Fuc	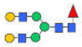 α-anomer
894.3315(2+)	31.18	1787.6552	1787.6579	-2	Gal$_2$(GlcNAc)$_2$Man$_3$(GlcNAc)$_2$Fuc	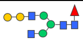 α-anomer
894.3314(2+)	31.83	1787.6550	1787.6579	-2	Gal$_2$(GlcNAc)$_2$Man$_3$(GlcNAc)$_2$Fuc	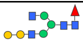 β-anomer
1056.3837(2+)	31.83	2111.7596	2111.7636	-2	Gal$_4$(GlcNAc)$_2$Man$_3$(GlcNAc)$_2$Fuc	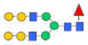 β-anomer
1056.3839(2+)	34.37	2111.7600	2111.7636	-2	Gal$_4$(GlcNAc)$_2$Man$_3$(GlcNAc)$_2$Fuc	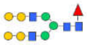 α-anomer
954.8448(2+)	29.47	1908.6818	1908.6842	-1	Gal$_2$(GlcNAc)Man$_5$(GlcNAc)$_2$Fuc	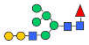 β-anomer
954.8447(2+)	32.31	1908.6816	1908.6842	-1	Gal$_2$(GlcNAc)Man$_5$(GlcNAc)$_2$Fuc	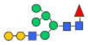 α-anomer
975.3576(2+)	29.80	1949.7074	1949.7107	-2	Gal$_3$(GlcNAc)$_2$Man$_3$(GlcNAc)$_2$Fuc	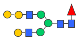 β-anomer
975.3570(2+)	29.93	1949.7062	1949.7107	-2	Gal$_3$(GlcNAc)$_2$Man$_3$(GlcNAc)$_2$Fuc	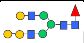 β-anomer
975.3578(2+)	32.27	1949.7078	1949.7107	-2	Gal$_3$(GlcNAc)$_2$Man$_3$(GlcNAc)$_2$Fuc	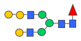 α-anomer
975.3575(2+)	32.70	1949.7072	1949.7107	-2	Gal$_3$(GlcNAc)$_2$Man$_3$(GlcNAc)$_2$Fuc	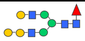 α-anomer
1048.3865(2+)	31.05	2095.7652	2095.7686	-2	Gal$_3$(GlcNAc)$_2$Man$_3$(GlcNAc)$_2$Fuc$_2$	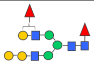 β-anomer
1048.3865(2+)	31.83	2095.7652	2095.7686	-2	Gal$_3$(GlcNAc)$_2$Man$_3$(GlcNAc)$_2$Fuc$_2$	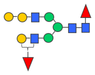 β-anomer
1048.3871(2+)	33.29	2095.7664	2095.7686	-1	Gal$_3$(GlcNAc)$_2$Man$_3$(GlcNAc)$_2$Fuc$_2$	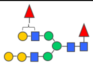 α-anomer

1048.3870(2+)	33.98	2095.7662	2095.7686	-1	$Gal_3(GlcNAc)_2Man_3(GlcNAc)_2Fuc_2$	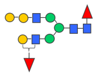 α-anomer
711.7654(2+)	30.12	1422.5230	1422.5257	0	$Gal(GlcNAc)Man_3(GlcNAc)_2Fuc$	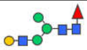 β-anomer
711.7656(2+)	35.59	1422.5234	1422.5257	-2	$Gal(GlcNAc)Man_3(GlcNAc)_2Fuc$	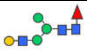 α-anomer
1319.9780(2+)	35.09	2638.9482	2638.9486	0	$Gal_6(GlcNAc)_3Man_3(GlcNAc)_2Fuc$	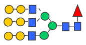 β-anomer
1319.9773(2+)	37.64	2638.9468	2638.9486	-1	$Gal_6(GlcNAc)_3Man_3(GlcNAc)_2Fuc$	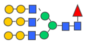 α-anomer
1238.9519(2+)	33.85	2476.8960	2476.8958	0	$Gal_5(GlcNAc)_3Man_3(GlcNAc)_2Fuc$	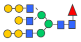 β-anomer
1238.9500(2+)	36.51	2476.8922	2476.8958	-2	$Gal_5(GlcNAc)_3Man_3(GlcNAc)_2Fuc$	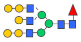 α-anomer
1238.9506(2+)	38.82	2476.8934	2476.8958	-1	$Gal_5(GlcNAc)_3Man_3(GlcNAc)_2Fuc$	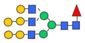 α-anomer
1157.9243(2+)	32.70	2314.8408	2314.8429	-1	$Gal_4(GlcNAc)_3Man_3(GlcNAc)_2Fuc$	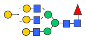 β-anomer
1157.9240(2+)	33.76	2314.8402	2314.8429	-1	$Gal_4(GlcNAc)_3Man_3(GlcNAc)_2Fuc$	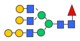 β-anomer
1157.9237(2+)	35.04	2314.8396	2314.8429	-2	$Gal_4(GlcNAc)_3Man_3(GlcNAc)_2Fuc$	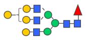 α-anomer
1157.9238(2+)	36.32	2314.8384	2314.8429	-2	$Gal_4(GlcNAc)_3Man_3(GlcNAc)_2Fuc$	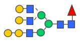 α-anomer
792.7915(2+)	25.17	1584.5752	1584.5785	-2	$Gal(GlcNAc)Man_4(GlcNAc)_2Fuc$	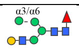 β-anomer
792.7921(2+)	26.20	1584.5764	1584.5785	-1	$(GlcNAc)Man_5(GlcNAc)_2Fuc$	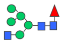 β-anomer
792.7917(2+)	27.74	1584.5756	1584.5785	-2	$Gal(GlcNAc)Man_4(GlcNAc)_2Fuc$	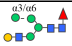 α-anomer
792.7918(2+)	28.90	1584.5758	1584.5785	-2	$(GlcNAc)Man_5(GlcNAc)_2Fuc$	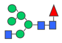 α-anomer
792.7918(2+)	32.65	1584.5758	1584.5785	-2	$Gal_2(GlcNAc)Man_3(GlcNAc)_2Fuc$	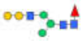 α-anomer
792.7917(2+)	37.29	1584.5756	1584.5785	-2	$Gal_2(GlcNAc)Man_3(GlcNAc)_2Fuc$	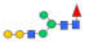 α-anomer
732.2789(2+)	25.30	1463.5500	1463.5523	-2	$(GlcNAc)_2Man_3(GlcNAc)_2Fuc$	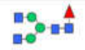 β-anomer
732.2789(2+)	28.00	1463.5500	1463.5523	-2	$(GlcNAc)_2Man_3(GlcNAc)_2Fuc$	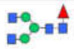 α-anomer
813.3050(2+)	26.41	1625.6022	1625.6051	-2	$Gal(GlcNAc)_2Man_3(GlcNAc)_2Fuc$	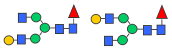 β-anomer
813.3049(2+)	29.13	1625.6020	1625.6051	-2	$Gal(GlcNAc)_2Man_3(GlcNAc)_2Fuc$	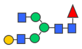 α-anomer
813.3054(2+)	29.32	1625.6030	1625.6051	-1	$Gal(GlcNAc)_2Man_3(GlcNAc)_2Fuc$	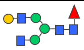 α-anomer
966.8499(2+)	38.78	1932.6920	1932.6954	-2	$(Neu5Gc)Gal(GlcNAc)_2Man_3(GlcNAc)_2Fuc$	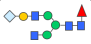 β-anomer
966.8502(2+)	42.05	1932.6926	1932.6954	-1	$(Neu5Gc)Gal(GlcNAc)_2Man_3(GlcNAc)_2Fuc$	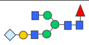 α-anomer
966.8503(2+)	42.47	1932.6928	1932.6954	-1	$(Neu5Gc)Gal(GlcNAc)_2Man_3(GlcNAc)_2Fuc$	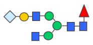 α-anomer
1047.8765(2+)	39.83	2094.7452	2094.7482	-2	$(Neu5Gc)Gal_2(GlcNAc)_2Man_3(GlcNAc)_2Fuc$	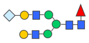 β-anomer
1047.8768(2+)	43.35	2094.7458	2094.7482	-1	$(Neu5Gc)Gal_2(GlcNAc)_2Man_3(GlcNAc)_2Fuc$	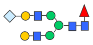 α-anomer

894.3315(2+)	27.59	1787.6552	1787.6579	-2	Gal$_2$(GlcNAc)$_2$Man$_3$(GlcNAc)$_{24}$Fuc	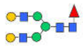 β-anomer
894.3317(2+)	28.59	1787.6556	1787.6579	-1	Gal$_2$(GlcNAc)$_2$Man$_3$(GlcNAc)$_2$Fuc	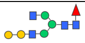 β-anomer
894.3311(2+)	30.31	1787.6544	1787.6579	-2	Gal$_2$(GlcNAc)$_2$Man$_3$(GlcNAc)$_2$Fuc	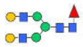 α-anomer
894.3313(2+)	31.18	1787.6548	1787.6579	-2	Gal$_2$(GlcNAc)$_2$Man$_3$(GlcNAc)$_2$Fuc	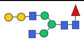 α-anomer
894.3317(2+)	31.83	1787.6556	1787.6579	-1	Gal$_2$(GlcNAc)$_2$Man$_3$(GlcNAc)$_2$Fuc	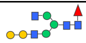 α-anomer
946.3365(2+)	38.02	1891.6652	1891.6689	-2	(Neu5Gc)Gal(GlcNAc)Man$_4$(GlcNAc)$_2$Fuc	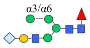 β-anomer
946.3372(2+)	41.31	1891.6666	1891.6689	-1	(Neu5Gc)Gal(GlcNAc)Man$_4$(GlcNAc)$_2$ Fuc	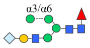 α-anomer
1027.3631(2+)	39.70	2053.7184	2053.7217	-2	(Neu5Gc)Gal(GlcNAc)Man$_5$(GlcNAc)$_2$Fuc	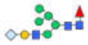 β-anomer
1027.3633(2+)	43.10	2053.7188	2053.7217	-1	(Neu5Gc)Gal(GlcNAc)Man$_5$(GlcNAc)$_2$Fuc	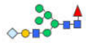 α-anomer
1120.9048(2+)	42.21	2240.8018	2240.8061	-2	(Neu5Ac)Gal$_3$(GlcNAc)$_2$Man$_3$(GlcNAc)$_2$Fuc	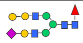 β-anomer
1120.9054(2+)	44.33	2240.8030	2240.8061	-2	(Neu5Gc)Gal$_2$(GlcNAc)$_2$Man$_3$(GlcNAc)$_2$Fuc$_2$	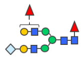 α-anomer
1120.9053(2+)	45.33	2240.8028	2240.8061	-2	(Neu5Ac)Gal$_3$(GlcNAc)$_2$Man$_3$(GlcNAc)$_2$Fuc<br>(Neu5Gc)Gal$_2$(GlcNAc)$_2$Man$_3$(GlcNAc)$_2$Fuc$_2$	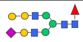 α-anomer<br>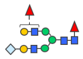 α-anomer
1128.9019(2+)	41.88	2256.7960	2256.8011	-2	(Neu5Gc)Gal$_3$(GlcNAc)$_2$Man$_3$(GlcNAc)$_2$Fuc	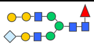 β-anomer
1128.9027(2+)	44.95	2256.7976	2256.8011	-2	(Neu5Gc)Gal$_3$(GlcNAc)$_2$Man$_3$(GlcNAc)$_2$Fuc	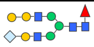 α-anomer
1128.9025(2+)	48.28	2256.7972	2256.8011	-2	(Neu5Gc)Gal$_3$(GlcNAc)$_2$Man$_3$(GlcNAc)$_2$Fuc	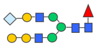 β-anomer
1128.9026(2+)	50.52	2256.7974	2256.8011	-2	(Neu5Gc)Gal$_3$(GlcNAc)$_2$Man$_3$(GlcNAc)$_2$Fuc	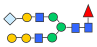 α-anomer

* Structural isomers of glycans are identified by MS/MS analyses, and the LC retention times are listed according to the chromatographic peaks of the extracted ion chromatograms (EICs).

Table S9. Predicted masses of glycopeptides and released glycans containing Neu5Ac, Neu5Gc and α-Gal

Glycopeptide *m/z* (charge)	Peptide	Peptide $[MH]^+$	Human-compatible Glycan	Non-human Glycan	Glycan (Delta)	Glycan m/z (charge)
913.3575(3+)<br>1369.5323(2+)	EEQYNSTYR	1189.5120	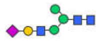<br>(Neu5Ac)Gal(GlcNAc)Man$_3$(GlcNAc)$_2$		1548.5448	523.1930(3+)<br>784.2855(2+)
967.3751(3+)<br>1450.5587(2+)	EEQYNSTYR	1189.5120	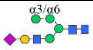<br>(Neu5Ac)Gal(GlcNAc)Man$_4$(GlcNAc)$_2$	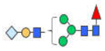<br>(Neu5Gc)Gal(GlcNAc)Man$_3$(GlcNAc)$_2$Fuc	1710.5976	577.2106(3+)<br>865.3119(2+)
1021.3927(3+)<br>1531.5852(2+)	EEQYNSTYR	1189.5120	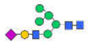<br>Neu5AcHex$_6$(HexNAc)$_3$	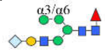<br>(Neu5Gc)Gal(GlcNAc)Man$_4$(GlcNAc)$_2$Fuc	1872.6505	631.2282(3+)<br>946.3384(2+)
981.0506(3+)<br>1471.0720(2+)	EEQYNSTYR	1189.5120	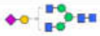<br>(Neu5Ac)Gal(GlcNAc)$_2$Man$_3$(GlcNAc)$_2$		1751.6242	590.8860(3+)<br>885.8252(2+)
1035.0670(3+)<br>1552.0984(2+)	EEQYNSTYR	1189.5120	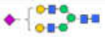<br>(Neu5Ac)Gal(GlcNAc)$_2$Man$_3$(GlcNAc)$_2$	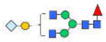<br>(Neu5Gc)Gal(GlcNAc)$_2$Man$_3$(GlcNAc)$_2$Fuc	1913.6770	644.9037(3+)<br>966.8516(2+)
1132.1000(3+)<br>1697.6461(2+)	EEQYNSTYR	1189.5120	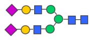<br>(Neu5Ac)$_2$Hex$_5$(HexNAc)$_4$		2204.7724	741.9355(3+)<br>1112.3993(2+)
962.0434(3+)<br>1442.5613(2+)	EEQYNSTYR	1189.5120	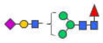<br>(Neu5Ac)Gal(GlcNAc)Man$_3$(GlcNAc)$_2$Fuc		1694.6027	571.8789(3+)<br>857.3145(2+)
1016.0611(3+)<br>1523.5877(2+)	EEQYNSTYR	1189.5120	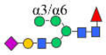<br>(Neu5Ac)Gal(GlcNAc)Man$_4$(GlcNAc)$_2$Fuc		1856.6556	625.8965(3+)<br>938.3409(2+)
1070.0787(3+)<br>1604.6141(2+)	EEQYNSTYR	1189.5120	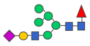<br>(Neu5Ac)Gal(GlcNAc)Man$_5$(GlcNAc)$_2$Fuc		2018.7084	679.9141(3+)<br>1019.3673(2+)
1029.7366(3+)<br>1544.1010(2+)	EEQYNSTYR	1189.5120	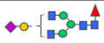<br>(Neu5Ac)Gal(GlcNAc)$_2$Man$_3$(GlcNAc)$_2$Fuc		1897.6821	639.5721(3+)<br>958.8542(2+)
1083.7542(3+)<br>1625.1274(2+)	EEQYNSTYR	1189.5120	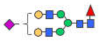<br>(Neu5Ac)Gal$_2$(GlcNAc)$_2$Man$_3$(GlcNAc)$_2$Fuc		2059.7349	693.5897(3+)<br>1039.8806(2+)
1180.7772(3+)<br>1770.6619(2+)	EEQYNSTYR	1189.5120	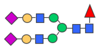<br>(Neu5Ac)$_2$Gal$_2$(GlcNAc)$_2$Man$_3$(GlcNAc)$_2$Fuc		2350.8304	790.6215(3+)<br>1185.4283(2+)
918.6891(3+)<br>1377.5298(2+)	EEQYNSTYR	1189.5120		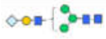<br>(Neu5Gc)Gal(GlcNAc)Man$_3$(GlcNAc)$_2$	1564.5397	528.5246(3+)<br>792.2830(2+)
972.7068(3+)<br>1458.5562(2+)	EEQYNSTYR	1189.5120		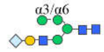<br>(Neu5Gc)Gal(GlcNAc)Man$_4$(GlcNAc)$_2$	1726.5926	582.5422(3+)<br>873.3094(2+)
1026.7244(3+)<br>1539.5826(2+)	EEQYNSTYR	1189.5120		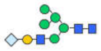<br>(Neu5Gc)Gal(GlcNAc)Man$_5$(GlcNAc)$_2$	1888.6454	636.5598(3+)<br>954.3358(2+)
986.382(3+)<br>1479.0695(2+)	EEQYNSTYR	1189.5120		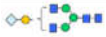<br>(Neu5Gc)Gal(GlcNAc)$_2$Man$_3$(GlcNAc)$_2$	1767.6191	596.2177(3+)<br>893.8227(2+)
1040.3999(3+)<br>1560.0959(2+)	EEQYNSTYR	1189.5120		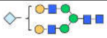<br>(Neu5Gc)Gal$_2$(GlcNAc)$_2$Man$_3$(GlcNAc)$_2$	1929.6719	650.2353(3+)<br>974.8491(2+)
1142.7633(3+)<br>1713.6411(2+)	EEQYNSTYR	1189.5120		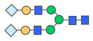<br>(Neu5Gc)$_2$Gal$_2$(GlcNAc)$_2$Man$_3$(GlcNAc)$_2$	2236.7623	752.5988(3+)<br>1128.3943(2+)
967.3751(3+)<br>1450.5588(2+)	EEQYNSTYR	1189.5120	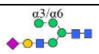<br>(Neu5Ac)Gal(GlcNAc)Man$_4$(GlcNAc)$_2$	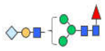<br>(Neu5Gc)Gal(GlcNAc)Man$_3$(GlcNAc)$_2$Fuc	1710.5977	577.2106(3+)<br>865.3119(2+)
1021.3927(3+)<br>1531.5852(2+)	EEQYNSTYR	1189.5120		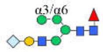<br>(Neu5Gc)Gal(GlcNAc)Man$_4$(GlcNAc)$_2$Fuc	1872.6505	631.2282(3+)<br>946.3384(2+)
1075.4103(3+)<br>1612.6116(2+)	EEQYNSTYR	1189.5120		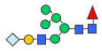<br>(Neu5Gc)Gal(GlcNAc)Man$_5$(GlcNAc)$_2$Fuc	2034.7033	685.2458(3+)<br>1027.3648(2+)
1035.0682(3+)<br>1552.0984(2+)	EEQYNSTYR	1189.5120	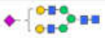<br>(Neu5Ac)Gal(GlcNAc)$_2$Man$_3$(GlcNAc)$_2$	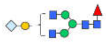<br>(Neu5Gc)Gal(GlcNAc)$_2$Man$_3$(GlcNAc)$_2$Fuc	1913.6770	644.9037(3+)<br>966.8516(2+)
1089.0858(3+)<br>1633.1248(2+)	EEQYNSTYR	1189.5120		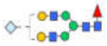<br>(Neu5Gc)Gal$_2$(GlcNAc)$_2$Man$_3$(GlcNAc)$_2$Fuc	2075.7298	698.9213(3+)<br>1047.8780(2+)

				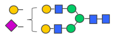<br>(Neu5Ac)Gal$_3$(GlcNAc)$_2$Man$_3$(GlcNAc)$_2$		
1191.4496(3+)<br>1786.6700(2+)	EEQYNSTYR	1189.5120		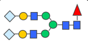<br>(Neu5Gc)$_2$Gal$_2$(GlcNAc)$_2$Man$_3$(GlcAc)$_2$Fuc	2382.8202	801.2848(3+)<br>1201.4232(2+)
919.0293(3+)<br>1378.0400(2+)	EEQYNSTYR	1189.5120	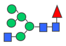<br>(GlcNAc)$_2$Man$_5$(GlcNAc)$_2$Fuc	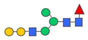<br>Gal$_2$(GlcNAc)Man$_3$(GlcNAc)$_2$Fuc	1565.5601	792.7932(2+)<br>528.8647(3+)
986.7224(3+)<br>1479.5796(2+)	EEQYNSTYR	1189.5120	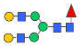<br>Gal$_2$(GlcNAc)$_2$Man$_3$(GlcNAc)$_2$Fuc	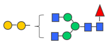<br>Gal$_2$(GlcNAc)$_2$Man$_3$(GlcNAc)$_2$Fuc	1768.6395	596.5579(3+)<br>894.3329(2+)
1040.7400(3+)<br>1560.6061(2+)	EEQYNSTYR	1189.5120		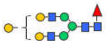<br>Gal$_3$(GlcNAc)$_2$Man$_3$(GlcNAc)$_2$Fuc	1930.6923	650.5755(3+)<br>975.3593(2+)
1094.7576(3+)<br>1641.6325(2+)	EEQYNSTYR	1189.5120		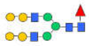<br>Gal$_4$(GlcNAc)$_2$Man$_3$(GlcNAc)$_2$Fuc	2092.7452	704.5931(3+)<br>1056.3857(2+)
1089.4260(3+)<br>1633.6350(2+)	EEQYNSTYR	1189.5120		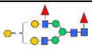<br>Gal$_3$(GlcNAc)$_2$FucMan$_3$(GlcNAc)$_2$Fuc	2076.7502	699.2614(3+)<br>1048.3882(2+)
1137.7718(3+)<br>1706.1538(2+)	EEQYNSTYR	1189.5120		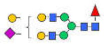<br>(Neu5Ac)Gal$_3$(GlcNAc)$_2$Man$_3$(GlcNAc)$_2$Fuc<br>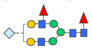<br>(Neu5Gc)Gal$_2$(GlcNAc)$_2$Man$_3$(GlcNAc)$_2$Fuc$_2$	2221.7877	747.6073(3+)<br>1120.9070(2+)
1143.1035(3+)<br>1714.1513(2+)	EEQYNSTYR	1189.5120		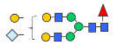<br>(Neu5Gc)Gal$_3$(GlcNAc)$_2$Man$_3$(GlcNAc)$_2$Fuc<br>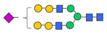<br>(Neu5Ac)Gal$_4$(GlcNAc)$_2$Man$_3$(GlcNAc)$_2$	2237.7827	752.9389(3+)<br>1128.9045(2+)
1162.4507(3+)<br>1743.1721(2+)	EEQYNSTYR	1189.5120		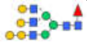<br>Gal$_4$(GlcNAc)$_3$Man$_3$(GlcNAc)$_2$Fuc	2295.8245	772.2862(3+)<br>1157.9254(2+)
1216.4684(3+)<br>1824.1986(2+)	EEQYNSTYR	1189.5120		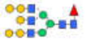<br>Gal$_5$(GlcNAc)$_3$Man$_3$(GlcNAc)$_2$Fuc	2457.8774	826.3038(3+)<br>1238.9512(2+)
1270.4860(3+)<br>1905.2250(2+)	EEQYNSTYR	1189.5120		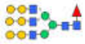<br>Gal$_6$(GlcNAc)$_3$Man$_3$(GlcNAc)$_2$Fuc	2619.9302	880.3214(3+)<br>1319.9782(2+)
839.5939(4+)<br>1120.1226(3+)	TKPREEQYN<br>STYR	1671.8085	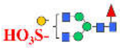<br><br>Gal(GlcNAc)$_2$Man$_3$(GlcNAc)$_2$FucSO$_3$H		1686.5435	569.1925(3+)<br>853.2849(2+)
880.1071(4+)<br>1174.1402(3+)	TKPREEQYN<br>STYR	1671.8085	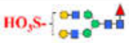<br><br>Gal$_2$(GlcNAc)$_2$Man$_3$(GlcNAc)$_2$FucSO$_3$H	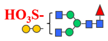<br><br>Gal$_2$(GlcNAc)$_2$Man$_3$(GlcNAc)$_2$FucSO$_3$H	1848.5963	623.2101(3+)<br>934.3113(2+)
959.3895(4+)<br>1279.8501(3+)	MNSLQSNDT<br>AIYYCAR	1906.8422		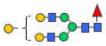<br>Gal$_3$(GlcNAc)$_2$Man$_3$(GlcNAc)$_2$Fuc	1930.6923	650.5755(3+)<br>975.3593(2+)
999.9027 (4+)<br>1333.8677(3+)	MNSLQSNDT<br>AIYYCAR	1906.8422		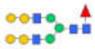<br>Gal$_4$(GlcNAc)$_2$Man$_3$(GlcNAc)$_2$Fuc	2092.7452	704.5931(3+)<br>1056.3857(2+)
995.9040(4+)<br>1328.5360(3+)	MNSLQSNDT<br>AIYYCAR	1906.8422		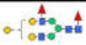<br>Gal$_3$(GlcNAc)$_2$FucMan$_3$(GlcNAc)$_2$Fuc	2076.7502	699.2614(3+)<br>1048.3882(2+)
1032.1634(4+)<br>1376.8819(3+)	MNSLQSNDT<br>AIYYCAR	1906.8422		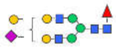<br>(Neu5Ac)Gal$_3$(GlcNAc)$_2$Man$_3$(GlcNAc)$_2$Fuc<br>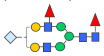<br>(Neu5Gc)Gal$_2$(GlcNAc)$_2$Man$_3$(GlcNAc)$_2$Fuc$_2$	2221.7877	747.6073(3+)<br>1120.9070(2+)
1036.1621(4+)<br>1382.2135(3+)	MNSLQS[88]ND<br>TAIYYCAR	1906.8422		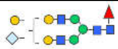<br>(Neu5Gc)Gal$_3$(GlcNAc)$_2$Man$_3$(GlcNAc)$_2$Fuc	2237.7827	752.9389(3+)<br>1128.9045(2+)
1050.6725(4+)<br>1401.5608(3+)	MNSLQSNDT<br>AIYYCAR	1906.8422		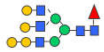<br>Gal$_4$(GlcNAc)$_3$Man$_3$(GlcNAc)$_2$Fuc	2295.8245	772.2862(3+)<br>1157.9254(2+)

1091.1858(4+)<br>1455.5784(3+)	MNSLQS**N**DT<br>AIYYCAR	1906.8422		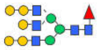<br>$Gal_5(GlcNAc)_3Man_3(GlcNAc)_2Fuc$	2457.8774	826.3038(3+)<br>1238.9512(2+)
1131.6990(4+)<br>1509.5960(3+)	MNSLQS**N**DT<br>AIYYCAR	1906.8422		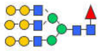<br>$Gal_6(GlcNAc)_3Man_3(GlcNAc)_2Fuc$	2619.9302	880.3214(3+)<br>1319.9782(2+)

Table S10 Distribution of isomeric non-human glycans of the mAb drugs derived from murine myeloma cells

m/z (2+)	Glycan	Infliximab		Palivizumab		Golimumab		Cetuximab	
		RT	Area	RT	Area	RT	Area	RT	Area
861.3038	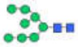 β-anomer	-	-	22.76	119629	22.83	210160	-	-
	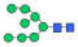 α-anomer	24.92	80134	24.88	273350	24.86	425634	-	-
780.2773	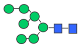 β-anomer	-	-	22.71	49342	22.87	84833	-	-
	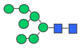 α-anomer	-	-	24.75	162808	24.90	222929	24.92	118104
699.2510	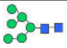 β-anomer	-	-	-	-	22.92	53570	23.79	170093
	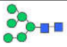 α-anomer	-	-	-	-	24.82	71893	24.92	64348
618.2245	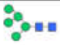 β-anomer	27.36	2602545	27.34	6932377	27.27	1207695	27.30	7548742
	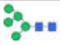 α-anomer	30.99	8206678	31.11	18341482	29.16	20983500	31.10	22703572
719.7642	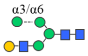 β-anomer	22.66	549423	22.66	577457	22.68	226966	22.61	266954
	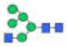 β-anomer	23.39	2883861	23.35	21823	23.37	416479	23.39	441290
	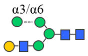 α-anomer	25.09	2005637	25.05	1326435	25.02	523180	25.22	565606
	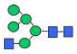 α-anomer	25.96	6884633	25.89	72655	25.99	882563	26.03	937930
800.7906	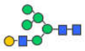 β-anomer	24.51	2083552	24.52	2132682	24.44	852946	24.47	1819127
	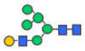 α-anomer	27.16	4699651	27.05	4735253	27.06	1744933	27.05	3825138
557.7114	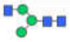 β-anomer	22.27	4007998	4654717	5647575	22.21	418790	22.16	780156
	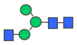 β-anomer	22.50	1026364	22.42	992858	22.48	1440538	22.39	367671
	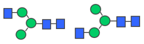 α-anomer	24.88	10632888	24.84	13446841	24.86	4968905	24.88	3911567
659.2511	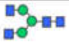 β-anomer	22.52	1519161	22.47	1061175	22.48	3592314	22.39	927700
	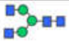 α-anomer	25.05	5924205	25.01	2171056	24.90	13839554	24.92	1465789
740.2775	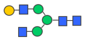 β-anomer	23.57	581302	23.53	1119564	23.55	4222742	23.44	591338
	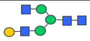 α-anomer	23.88	299856	23.91	452009	23.82	2032436	23.79	291513
	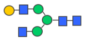 β-anomer	26.13	1498720	26.10	1525922	26.10	9378042	26.03	1057353
	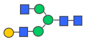 α-anomer	26.39	1113591	26.44	4699196	26.40	8161715	26.41	1424601
732.2800	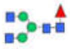 β-anomer	25.42	75184867	25.40	189250822	25.26	175616990	25.30	64499612
	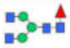 α-anomer	28.16	264854942	28.21	611669492	28.10	527839821	28.00	237926433
813.3064	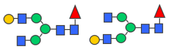 β-anomer	26.52	45180893	26.46	330125407	26.40	244875545	26.41	82762918
	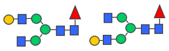 α-anomer	29.20	161584577	29.20	1042618779	29.12	796779576	29.13	266557558
792.2830	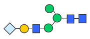 β-anomer	36.44	454031	36.48	68109	36.81	422799	-	-
	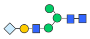 α-anomer	40.68	1991344	40.66	226944	41.07	1420149	-	-

	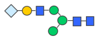 α-anomer	44.58	110884	44.48	50768	44.70	25544	-	-
873.3094	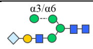 β-anomer	34.61	367030	-	-	34.69	443368	-	-
	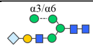 α-anomer	37.81	1585676	37.74	156582	38.22	2125184	-	-
954.3358	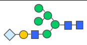 β-anomer	36.11	1397448	36.24	263105	36.52	1530567	36.86	44573
	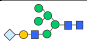 α-anomer	39.67	4718600	39.59	638974	40.03	4634669	40.54	334816
893.8227	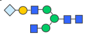 β-anomer	-	-	-	-	35.56	281623	-	-
	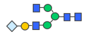 β-anomer	-	-	-	-	38.71	634856	-	-
	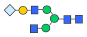 α-anomer	-	-	-	-	39.40	647471	-	-
	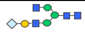 α-anomer	-	-	-	-	42.18	749008		
792.7932	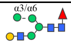 β-anomer	25.25	190261	25.23	398483	-	-	25.17	1198764
	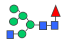 β-anomer	26.26	1024185	-	-	-	-	26.20	414044
	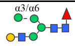 α-anomer	27.87	1444542	27.90	2683363	27.86	503224	27.74	5101737
	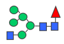 α-anomer	28.92	2845472	-	-	28.92	647398	28.90	1899684
	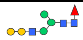 β-anomer	29.56	537647	31.34	1053999	-	-	29.93	228547
	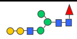 α-anomer	32.64	1762615	32.72	24586735	32.68	1313607	32.65	1177320
	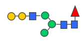 α-anomer	-	-	-	-	37.44	254475	37.29	1955001
873.8182	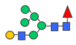 β-anomer	27.20	540755	27.18	1097719	27.15	149986	27.05	2255549
	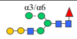 β-anomer	27.74	175936	27.77	452883	27.82	188494	27.64	1902974
	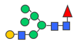 α-anomer	29.92	2536580	29.93	3682561	29.98	919678	29.84	8916967
	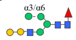 α-anomer	30.45	619826	30.43	2631711	30.50	668814	30.37	5804493
954.8448	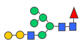 β-anomer	-	-	-	-	-	-	29.47	1221471
	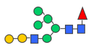 α-anomer	32.31	552144	32.39	1890540	32.40	777531	32.31	5797513
865.3119	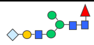 β-anomer	39.75	4021908	39.73	1871167	40.12	1085456	-	-
	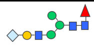 α-anomer	44.53	19703568	44.44	13126432	44.69	7542293	-	-
946.3384	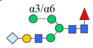 β-anomer	36.93	790341	37.04	256535	37.52	1354161	37.80	74911
	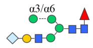 α-anomer	40.56	3348079	40.62	1330276	40.92	5513349	41.31	1065559
1027.3648	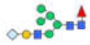 β-anomer	38.85	669815	38.84	244147	39.31	707683	39.70	185902
	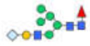 α-anomer	42.54	2885730	42.43	1070008	42.73	3117093	43.10	1113774
966.8516	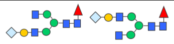 β-anomer	38.26	2386055	38.19	1363198	39.01	26414424	38.78	522291
	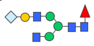 α-anomer	41.44	3453417	41.51	2617461	41.70	54192261	42.05	585441
	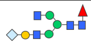 α-anomer	41.93	5631183	42.06	3218184	42.18	51983632	42.47	3080985
1047.8780	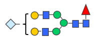 β-anomer	39.14	1329043	39.25	2719662	39.63	27824198	39.83	4410094
	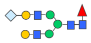 α-anomer	42.14	526651	41.98	1138531	42.36	10320203	42.65	822150
	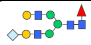 α-anomer	42.94	5153822	42.94	8460284	43.06	87198005	43.35	15207934

894.3329	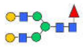 β-anomer	27.70	5493171	27.68	119301828	27.60	69014676	27.59	15320133
	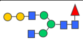 β-anomer	28.68	1574117	28.79	30063449	28.70	17162223	28.59	7152210
	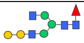 β-anomer	29.17	575691	29.23	17880141	29.16	2768753	29.13	1960081
	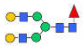 α-anomer	30.41	19373678	30.39	354206228	30.34	208273741	30.31	49593045
	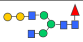 α-anomer	31.19	4243786	31.29	41949367	31.32	43354602	31.18	21090408
	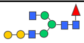 α-anomer	32.02	822301	31.99	8693165	32.04	8288642	31.83	5751910
975.3592	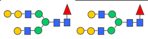 β-anomer	29.96	544882	29.93	19998237	29.84	17006751	29.93	9198175
	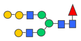 α-anomer	32.40	2155090	32.44	54212446	32.44	39485911	32.27	12397690
	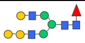 α-anomer	32.77	8655260	32.87	17658461	32.85	17246964	32.70	14197064
1056.3857	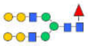 β-anomer	31.98	124584	31.89	10882246	31.95	8559300	31.83	51085347
	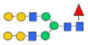 α-anomer	34.61	575879	34.70	36038759	34.39	31391600	34.37	158161153
1048.3871	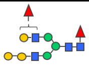 β-anomer	-	-	31.20	256597	31.17	220655	31.05	2366312
	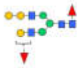 β-anomer	-	-	31.89	196138	31.87	437970	31.83	1430340
	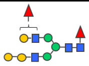 α-anomer	-	-	33.58	1330578	33.35	1518460	33.29	9367536
	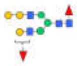 α-anomer	-	-	34.16	492899	33.98	467070	33.98	2730291
1120.9070	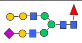 β-anomer	-	-	41.88	64258	-	-	42.21	695693
	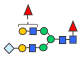 β-anomer	-	-	44.05	34567	44.08	173168	44.33	655488
	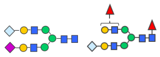 α-anomer	45.13	22229	45.14	377832	45.47	1042383	45.33	3171098
1128.9045	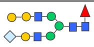 β-anomer	41.32	624622	41.46	2068310	41.70	13365273	41.88	22560351
	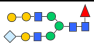 α-anomer	44.76	2115987	44.78	6554891	44.94	51209759	44.95	74906193
	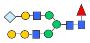 α-anomer	-	-	-	-	-	-	50.52	34948820
1157.9254	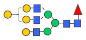 β-anomer	-	-	32.77	296373	-	-	32.70	524600
	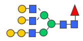 β-anomer	-	-	-	-	-	-	33.76	225092
	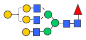 α-anomer	-	-	35.39	1778813	35.15	177541	35.04	2285175
	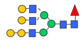 α-anomer	-	-	-	-	-	-	36.32	1260616
1238.9518	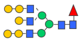 β-anomer	-	-	34.16	92359	34.14	10088	33.85	1849354
	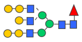 α-anomer	-	-	36.56	719350	36.57	62691	36.51	5818915
	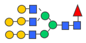 α-anomer	-	-	-	-	-	-	38.82	515992
1319.9782	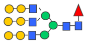 β-anomer	-	-	35.30	210319	-	-	35.09	4217033
	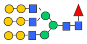 α-anomer	-	-	37.49	666443	37.85	110077	37.64	14845094

The peak areas are determined by PGC LC MS/MS and the extracted ion chromatograms using Thermo Xcalibur 4.1.
"-" donates an undetected peak.
